# The unusual cell wall of the Lyme disease spirochaete *Borrelia burgdorferi* is shaped by a tick sugar

**DOI:** 10.1038/s41564-021-01003-w

**Published:** 2021-11-24

**Authors:** Tanner G. DeHart, Mara R. Kushelman, Sherry B. Hildreth, Richard F. Helm, Brandon L. Jutras

**Affiliations:** 1 Department of Biochemistry, Virginia Tech, Blacksburg, VA USA; 2 Fralin Life Sciences Institute, Virginia Tech, Blacksburg, VA USA; 3 Molecular and Cellular Biology, Virginia Tech, Blacksburg, VA USA; 4 Translational Biology, Medicine, and Health, Virginia Tech, Blacksburg, VA USA; 5 Center for Emerging, Zoonotic and Arthropod-borne Pathogens, Virginia Tech, Blacksburg, VA USA

**Keywords:** Bacteriology, Cellular microbiology, Pathogens

## Abstract

Peptidoglycan—a mesh sac of glycans that are linked by peptides—is the main component of bacterial cell walls. Peptidoglycan provides structural strength, protects cells from osmotic pressure and contributes to shape. All bacterial glycans are repeating disaccharides of *N-*acetylglucosamine (Glc*N*Ac) β-(1–4)-linked to *N*-acetylmuramic acid (Mur*N*Ac). *Borrelia burgdorferi*, the tick-borne Lyme disease pathogen, produces glycan chains in which Mur*N*Ac is occasionally replaced with an unknown sugar. Nuclear magnetic resonance, liquid chromatography–mass spectroscopy and genetic analyses show that *B. burgdorferi* produces glycans that contain Glc*N*Ac–Glc*N*Ac. This unusual disaccharide is chitobiose, a component of its chitinous tick vector. Mutant bacteria that are auxotrophic for chitobiose have altered morphology, reduced motility and cell envelope defects that probably result from producing peptidoglycan that is stiffer than that in wild-type bacteria. We propose that the peptidoglycan of *B. burgdorferi* probably evolved by adaptation to obligate parasitization of a tick vector, resulting in a biophysical cell-wall alteration to withstand the atypical torque associated with twisting motility.

## Abstract (web summary)

Tick chitobiose is co-opted to build the cell wall of Lyme disease pathogen *Borrelia burgdorferi*.

## Main

The peptidoglycan sacculus protects the cytoplasmic contents of virtually all bacterial cells. Peptidoglycan architecture (rigid glycan strands, cross-linked by flexible peptides) is universal across bacterial taxa. Peptidoglycan glycans comprise a disaccharide repeat unit of N-acetylglucosamine (Glc*N*Ac) and *N*-acetylmuramic acid (Mur*N*Ac). Mur*N*Ac provides a C3 lactyl moiety that anchors peptide assembly. Glycan chain lengths of six to hundreds of disaccharide repeats are terminated at the reducing-end anomeric position by a 1,6-anhydro-*N*-acetylmuramic acid (anhMur*N*Ac) residue^1^. Although alterations in peptidoglycan peptide chemistry occur across the bacterial domain, deviations from the β-(1–4)-linked Glc*N*Ac–Mur*N*Ac disaccharide have not previously been reported.

The pathogenic spirochaete *B. burgdorferi* is estimated to cause more than 450,000 cases of Lyme disease each year, in the USA alone^2^. On transmission via the bite of an infected *Ixodes scapularis* tick, *B. burgdorferi*, which is an obligate parasitic bacterium, causes a biphasic infection. An acute stage characterized by ‘flu-like’ symptoms is followed by a severe late stage that can involve multiple organ systems^3,4^. Despite the public health burden posed by this ascending vector-borne disease, very little is known about what causes clinical symptoms.

*B. burgdorferi* lacks many of the classic virulence factors typically associated with invasive pathogens. One well-known feature, critical to *B. burgdorferi* pathogenesis, is the corkscrew-like motility that it uses to both escape immune cells and invade host tissues^5^. Endoflagella at each pole form a ribbon that wraps around the peptidoglycan sacculus. Motor rotation causes the flagella to torque the peptidoglycan, creating a backward wave that propels the bacterium forwards^6^. *B. burgdorferi* peptidoglycan, which has also been implicated in potentiating Lyme disease pathogenesis^7,8^, is thought to require unique feature(s) to counterbalance the immense flagellar stress. Previous reports describe the presence of ornithine (Orn) in the peptidoglycan stem peptide^7,9^, as well as several unidentifiable components, including an unknown *N*-acetylated hexose (Hex*N*Ac) linked to the Glc*N*Ac–Mur*N*Ac disaccharide in glycan strands^7^. The culprit responsible for this atypical alteration has remained unknown.

## Results

### *B. burgdorferi* glycan architecture

**Fig. 1:**
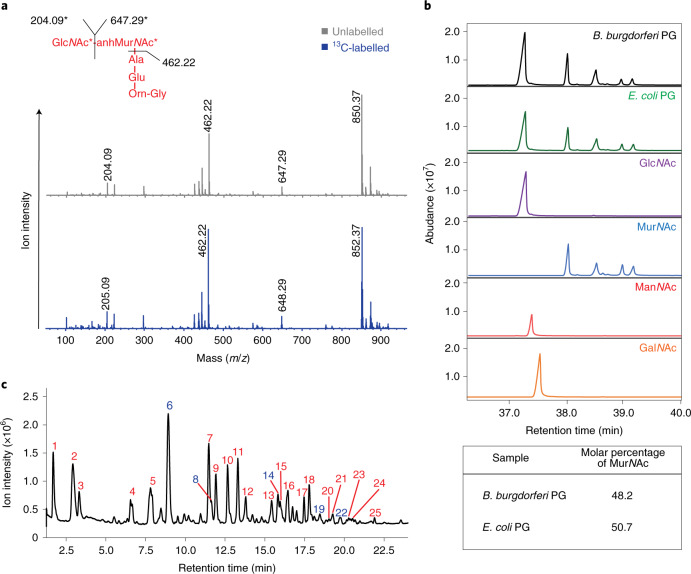
Elucidating the peptidoglycan glycan strand composition of *B. burgdorferi*. **a**, MS/MS of the Glc*N*Ac–Mur*N*Ac–AlaGluOrnGly muropeptide from *B. burgdorferi* 5A11 cultured in unlabelled (grey) and [1-¹³C]Man*N*Ac (blue), respectively. Fragmentation data confirm the location of the labelled carbon resides in the glycan component and not the stem peptide. **b**, Monosaccharide analysis of purified peptidoglycan isolated from *B. burgdorferi* 5A11 and *E. coli* K-12. Results were compared with reference standards Glc*N*Ac, Mur*N*Ac, Man*N*Ac and Gal*N*Ac (below). The inset table highlights the molar percentage of Mur*N*Ac present in each bacterial sample. **c**, LC–MS chromatogram of *B. burgdorferi* 5A11 peptidoglycan. *B. burgdorferi* peptidoglycan was purified, digested with mutanolysin and analysed by LC–MS. Each peak corresponds to one or more muropeptides of interest; peaks are labelled as red (Glc*N*Ac–Mur*N*Ac muropeptides) or blue (Hex*N*Ac–Glc*N*Ac–Mur*N*Ac muropeptides). Co-eluting peaks can be found in Supplementary Table 2.

**Extended Data Fig. 1:**
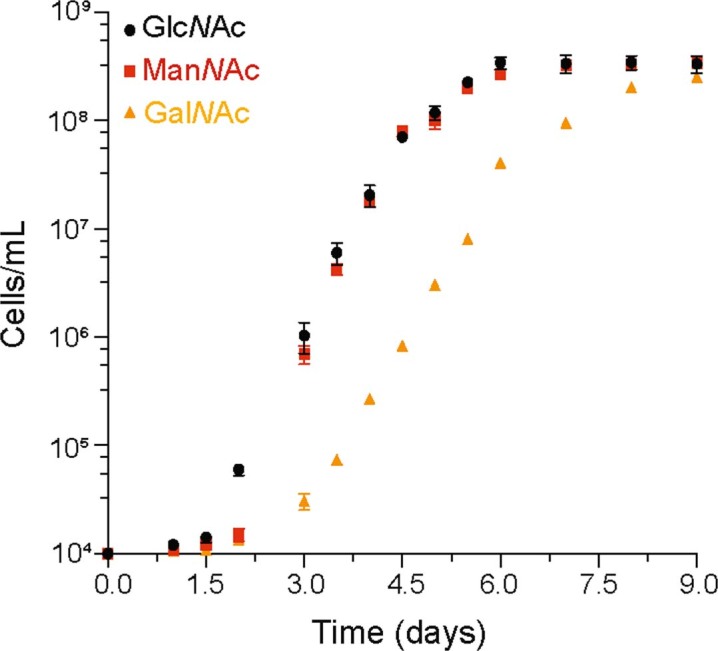
Growth of 5A11 *B. burgdorferi* in BSK-II complete media supplemented with Glc*N*Ac, Man*N*Ac, or Gal*N*Ac. Growth of 5A11 *B. burgdorferi* in BSK-II complete media supplemented with Glc*N*Ac, Man*N*Ac, or Gal*N*Ac. Values represent the mean and standard deviation of three independent cultures.

Similar to most parasitic bacteria*, B. burgdorferi* lacks many biosynthetic pathways and scavenges environmental molecules, including the peptidoglycan cell-wall precursor Glc*N*Ac^10^. Optimal in vitro growth thus requires that *B. burgdorferi* culture medium be supplemented with Glc*N*Ac^11^. By taking advantage of this auxotrophy, we reasoned that we would be able to substitute Glc*N*Ac with other *N*-acetylated sugars and identify the unknown hexose. Two candidates emerged for their ability to support growth in the absence of Glc*N*Ac: *N*-acetylmannosamine (Man*N*Ac) and *N*-acetylgalactosamine (Gal*N*Ac) (Extended Data Fig. 1 and refs. ^12–15^). As bacteria cultured with Man*N*Ac replicated at a similar rate and reached a comparable final density to Glc*N*Ac (Extended Data Fig. 1), we proceeded with metabolic labelling studies. We propagated *B. burgdorferi* 5A11 in culture medium containing [1-^13^C]Man*N*Ac and analysed the resulting muropeptide pool, obtained from purified and digested peptidoglycan, by liquid chromatography–mass spectroscopy (LC–MS) (Fig. 1a). Compared with muropeptide samples prepared from bacteria cultured with unlabelled Glc*N*Ac (Fig. 1a), [1-^13^C]Man*N*Ac-labelled muropeptides were identical and contained the expected mass shift, equally distributed across both Glc*N*Ac and Mur*N*Ac (Fig. 1a). This strongly suggested not only that are there pathway(s) capable of converting Man*N*Ac, and probably Gal*N*Ac, to Glc*N*Ac, but also that Man*N*Ac was an unlikely candidate. Next, we took a more holistic approach and performed monosaccharide analysis of purified peptidoglycan isolated from *B. burgdorferi* 5A11 and compared our results with various *N-*acetylated reference standards (Fig. 1b). Surprisingly, we detected only Glc*N*Ac and Mur*N*Ac, and the *B. burgdorferi* peptidoglycan sugar profile was identical to that of *Escherichia coli*. Collectively, our metabolic labelling studies and monosaccharide analysis suggested that the unknown Hex*N*Ac might be Glc*N*Ac.

### Muropeptide analysis of *B. burgdorferi* peptidoglycan

**Fig. 2:**
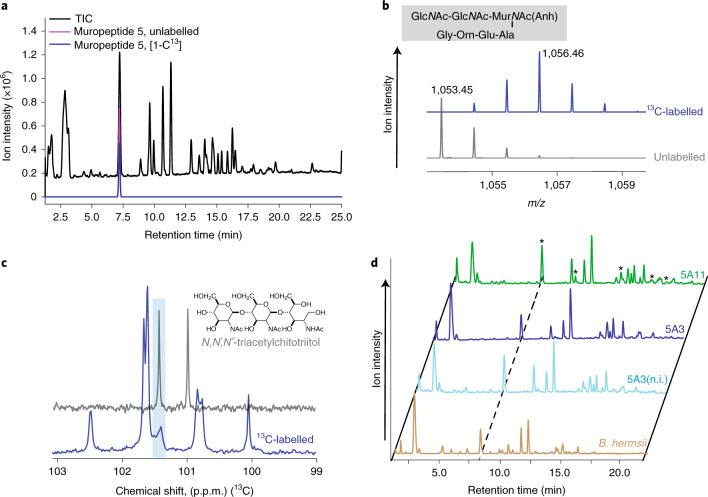
*B. burgdorferi* peptidoglycan glycan strands contain the trisaccharide G-G-anhM. **a**, LC–MS chromatogram of unlabelled *B. burgdorferi* 5A11 peptidoglycan. Total ion chromatogram is shown in black with an unlabelled and [1-¹³C]G-G-anhM muropeptide overlaid in pink and blue, respectively. **b**, LC–MS chromatogram of [1-¹³C]Glc*N*Ac metabolically labelled *B. burgdorferi* 5A11 peptidoglycan producing a mass shift corresponding to a G-G-anhM muropeptide. *B. burgdorferi* was cultured with unlabelled Glc*N*Ac or [1-¹³C]Glc*N*Ac before peptidoglycan purification and LC–MS analysis. The proposed G-G-anhM species 1,053 *m*/*z* in unlabelled peptidoglycan and the shifted mass to the predicted 1,056 *m*/*z* when labelled with [1-¹³C]Glc*N*Ac are shown. **c**, The ¹³C-labelled NMR of the anomeric region of 1-¹³C-labelled *B. burgdorferi* peptidoglycan and an *N*,*N*′,*N*′′-triacetylchitotriitol reference standard with the highlighted region (light blue) indicating a putative chemical shift for the non-reducing-end anomeric carbon. **d**, A comparative muropeptide analysis of peptidoglycan isolated from three clonal derivatives of *B. burgdorferi* and one strain of *B. hermsii*. Three laboratory strains of *B. burgdorferi*, two fully infectious clones of the B31-type strain (5A11, green; 5A3, purple) and non-infectious (n.i.) derivative of 5A3 (blue), as well as *B. hermsii* (yellow) were cultured to mid-log, peptidoglycan was purified, digested and muropeptide profiles compared by LC. All samples contained similar levels of G-G-anhM muropeptides (*).

**Extended Data Fig. 2:**
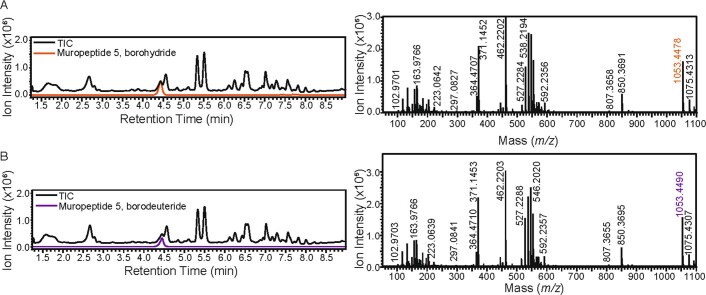
LCMS chromatogram of borohydride reduced 5A11 *B. burgdorferi* peptidoglycan. Extended Data Fig. 2: **a**) LCMS chromatogram of borohydride reduced 5A11 *B. burgdorferi* peptidoglycan (left) and corresponding MS/MS spectra of borohydride reduced muropeptide 5 (right). The TIC is shown in black and the abundance of boro-hydride reduced muropeptide 5 is shown in orange (1053.4277-1053.4699 m/z scanned). The precursor ion selected for MS/MS is shown in orange. **b**) LCMS chromatogram of borodeuteride reduced 5A11 *B. burgdorferi* peptidoglycan (left) and corresponding MS/MS spectra of muropeptide 5 (right). The TIC is shown in black and the abundance of borodeuteride reduced muropeptide 5 is shown in purple (1053.4289-1053.4711 m/z scanned). The precursor ion selected for MS/MS is shown in purple.

Previous analyses of the *B. burgdorferi* peptidoglycan cell wall separated muropeptides that were then analysed using targeted MS. This method captured the identity of ~45% of the *B. burgdorferi* muropeptides^7^. We reasoned that a more robust, untargeted approach to muropeptide analysis may provide further insights into composition of *B. burgdorferi* peptidoglycan. We created a new, high-resolution, LC–tandem MS (LC–MS/MS) method, which determined the identity of ~80% of the muropeptide pool in a fraction of the time (Fig. 1c). The LC step separated 25 discrete peaks, which contained 17 unique muropeptides (Fig. 1c and Supplementary Fig. 1), 5 of which contained the Hex*N*Ac–Glc*N*Ac–Mur*N*Ac moiety (Supplementary Tables 1–3 and Supplementary Figs. 7, 10, 19, 25 and 28). We coupled LC–MS from NaBH_4_-reduced muropeptides with data obtained from isotopically (NaBD_4_) labelled reduction products to provide mass markers and increased resolution for MS^2^ spectra in instances when more than one muropeptide eluted in the same fraction (Supplementary Table 2 and Extended Data Fig. 2). The latter confirmed that the unknown Hex*N*Ac was always adjacent to a Glc*N*Ac–anhMur*N*Ac residue, indicating that the new structure was at the terminus of glycan chains (Extended Data Fig. 2). Regardless of whether *B. burgdorferi* was cultured in medium containing labelled ([1-^13^C]Glc*N*Ac) or unlabelled Glc*N*Ac, the resulting LC–MS traces of each sample were identical, with mass shifts confirming that the label was distributed between the Glc*N*Ac and Mur*N*Ac residues (Fig. 2a,b). These data further implicated Glc*N*Ac as the unknown Hex*N*Ac because each sugar in the putative Glc*N*Ac–Glc*N*Ac–anhMur*N*Ac (G-G-anhM) trisaccharide was equally labelled (Fig. 2b).

Next, we carried out a series of proton nuclear magnetic resonance (H-NMR) experiments using *N*,*N*′,*N*′′-triacetylchitotriitol as a reference due to its structural similarity to G-G-anhM at the non-reducing end. As there are limitations associated with both purifying *B. burgdorferi* peptidoglycan and the detection limits for H-NMR, comparisons to the standard were in relation to the total muropeptide pool obtained from the [1-^13^C]Glc*N*Ac experiment and not to an individual muropeptide. Anomeric ^1^H chemical shifts (>5 p.p.m.) and coupling constants (~8 Hz) combined with ^13^C chemical shifts at ~100 p.p.m. firmly established all linkages as β-glycopyranosidic bonds between Glc*N*Ac residues (Fig. 2c). The only available hydroxyls for glycosidic bond formation are at positions 3, 4 and 6, with all known muropeptide linkages being (1–4)^1^. Although we cannot exclude the existence of non-canonical (1–3) and glycosidic (1–6) bonds, the data that we obtained (Fig. 2c) match that of a β-(1–4) linkage most closely. These findings establish that *B. burgdorferi* glycan chains terminate with G-G-anhM.

### Peptidoglycan composition is conserved among *Borrelia* strains and species

Laboratory strains of *B. burgdorferi* are known to lose extrachromosomal DNA during prolonged in vitro propagation^16^. This results in clonal heterogeneity, a reduction in biosynthetic capacity and avirulence^16–18^. To assess whether the peptidoglycan phenotype of *B. burgdorferi* 5A11 was due to a loss of extrachromosomal DNA, or an artefact of prolonged in vitro cultivation, we used whole-genome sequencing (WGS) to analyse the three commonly studied strains of B31. We sequenced strain 5A11, which is a fully infectious clone of the B31-type strain^10^, with all genetic elements that we used for all our peptidoglycan work thus far, strain 5A3, a fully infectious clonal derivative^16,18^ of 5A11 that is often used in the Lyme disease research field, and a high-passage variant of B31 that lacks many plasmids and is avirulent (Supplementary Table 4 and see data availability for repository links). WGS results were consistent with the expected nucleic acid content of each strain—5A11 and 5A3 were highly similar and carried a full repertoire of plasmids, whereas our high-passage strain lacked genetic elements associated with infectivity (Supplementary Table 4). Upon strain validation we isolated peptidoglycan from each and compared muropeptide profiles for the presence of G-G-anhM moieties. Each strain was almost identical and contained G-G-anhM (Fig. 2d).

Many different *Borrelia* genospecies cause Lyme disease. Our analysis, thus far, has been limited to derivatives of the B31-type strain. Instead of testing various Lyme disease-causing *Borrelia spp*., we analysed muropeptides from the relapsing fever pathogen *B. hermsii*, which is transmitted by *Ornithodoros* ticks^19^. Comparative analysis of muropeptide profiles, once again, clearly indicated the presence of G-G-anhM, despite differences in the abundance of other peptidoglycan fragments (Fig. 2d). Collectively, our studies demonstrate the first modification to the disaccharide repeat arrangement in bacterial glycans—a core biological feature of *Borrelia* peptidoglycan that is conserved, regardless of genome content or phylogenetic relatedness.

### Acquisition of Glc*N*Ac–Glc*N*Ac

**Fig. 3:**
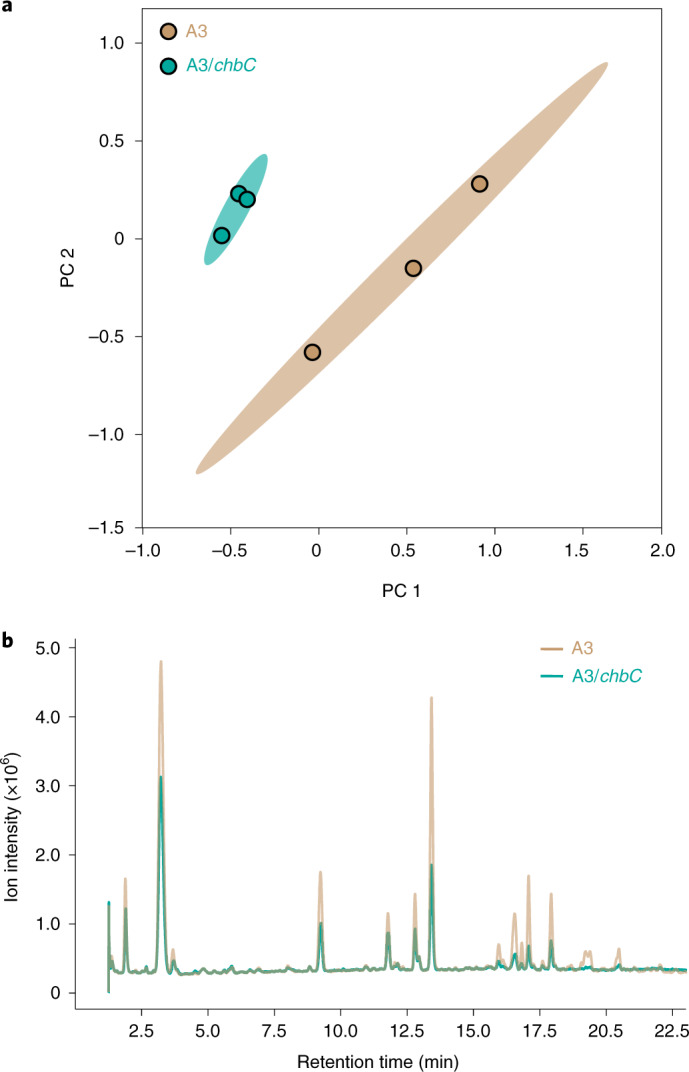
Comparative analysis of muropeptide profiles obtained from *B. burgdorferi* A3 and chitobiose transport mutant A3/*chbC*. **a**, Principal component (PC) analysis of 37 distinct muropeptide features collected from LC–MS data of three biological replicates, from WT A3 strain (tan) and A3/*chbC* (teal) peptidoglycan. **b**, Representative LC spectra from our comparative muropeptide analysis (in **a**) in which the amount of purified and injected peptidoglycan was normalized by the total number of cells present in each culture. Source data

**Extended Data Fig. 3:**
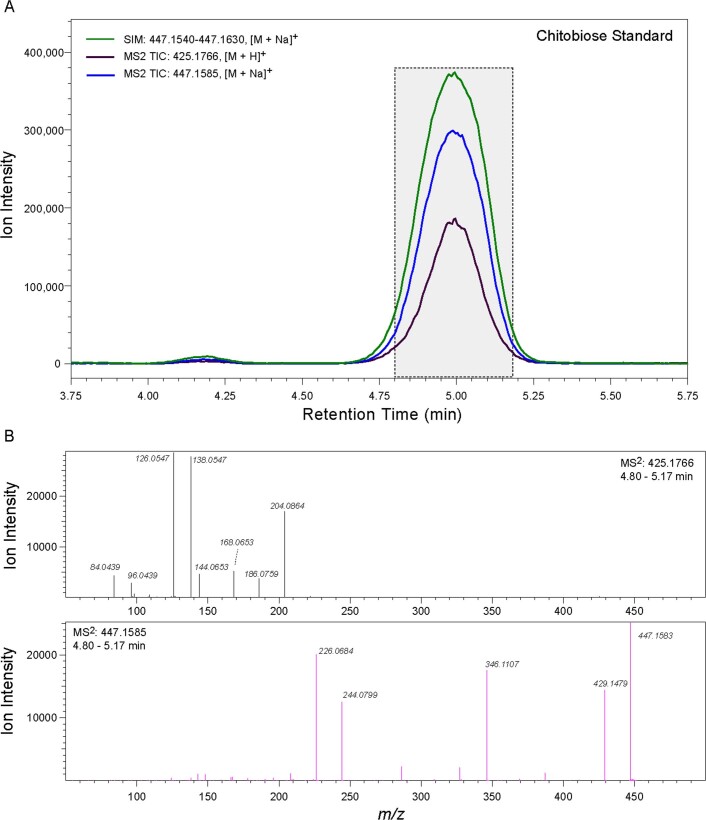
LC-MS Analysis of a Chitobiose Standard. LC-MS Analysis of a Chitobiose Standard. **a**) LC-MS traces using Selected Ion Monitoring (SIM) of the sodiated ion, and the Total Ion Chromatograms (TIC) for MS2 analysis of the protonated and sodiated species. **b**) MS2 spectra for the protonated (top) and sodiated (bottom) forms of chitobiose. Spectra summed over the time window indicated in the grey box (4.80-5.17 min).

**Extended Data Fig. 4:**
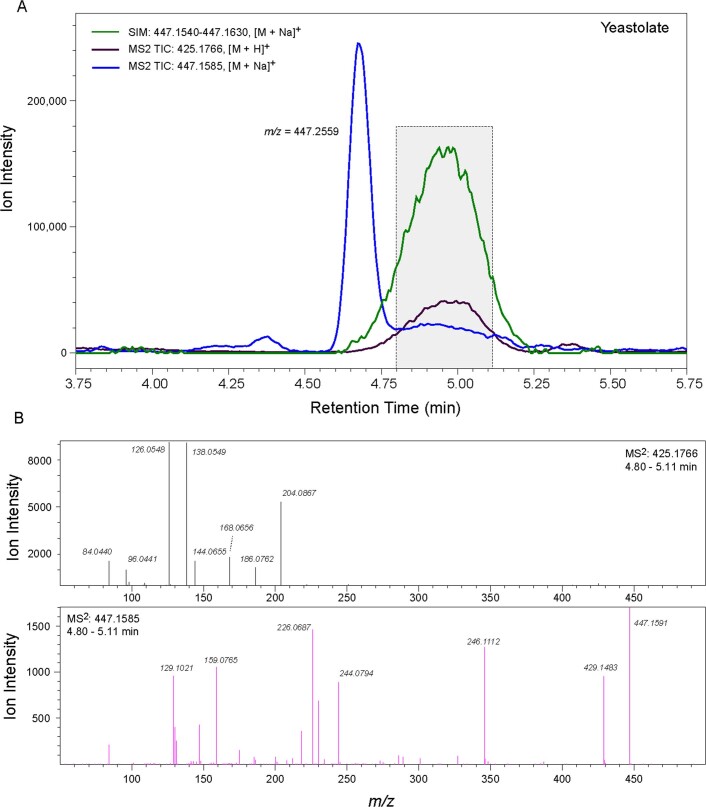
LC-MS Analysis of Commercial Autohydrolyzed Yeast (Yeastolate). Supplemental Fig. 10: LC-MS Analysis of Commercial Autohydrolyzed Yeast (Yeastolate). A) LC-MS traces using Selected Ion Monitoring (SIM) of the m/z values for chitobiose as the sodiated ion, and the Total Ion Chromatograms (TIC) for MS2 analysis of the protonated and sodiated species. B) MS2 spectra for the m/z values of the protonated (top) and sodiated (bottom) forms of chitobiose. Spectra summed over the time window indicated in the grey box (4.80-5.11 min).

*B. burgdorferi* can survive in the *I. scapularis* tick midgut for months between feeding cycles, so nutrient-rich blood is not a consistent carbon source. A plausible carbon source other than a blood meal is chitin, the primary component of the tick peritrophic membrane^20^. *N*,*N*′-Diacetylchitobiose (chitobiose) is the repeat unit of chitin, a disaccharide of Glc*N*Ac with a β-(1–4) glycosidic linkage, which is also present in BSK-II culture medium, routinely used to grow *Borrelia* spp. (Extended Data Figs. 3 and 4). The G-G-anhM sequence is essentially chitotriose with a 3-*O*-lactyl moiety. To assess the possibility that chitobiose is involved in *B. burgdorferi* peptidoglycan biosynthesis, we used a mutant bacterium (A3/*chbC)* that is incapable of importing Glc*N*Ac–Glc*N*Ac into the cytoplasm, as determined by isotopically labelled uptake experiments^21^. First, we used WGS to confirm that the parental A3 strain (analysed earlier) and the A3/*chbC* mutant strain were clonal and, with the exception of the hypervariable *vlsE* locus^22^ and the targeted deletion of *chbC* gene, the strains were genetically identical (Supplementary Table 5). Principal component analysis of the muropeptide profiles from three biological replicates—six different batches of culture—of the wild-type (WT) A3 and A3/*chbC* bacteria indicated homogeneity between replicates, but distinct features were apparent, suggesting that chitobiose transport impacts peptidoglycan composition (Fig. 3a). Comparative analysis of muropeptide identity and absolute abundance revealed that the parental strain contained more peptidoglycan per cell (Fig. 3b). Our interpretation of these findings is that breakdown products of chitobiose are used to build the *B. burgdorferi* peptidoglycan cell wall^13,15,21^ and a lack of chitobiose reduces the amounts of peptidoglycan. Importantly, even after we normalized for decrease in peptidoglycan (Online methods), we found that bacteria that were unable to import chitobiose from their environment had ~37% less G-G-anhM (Supplementary Table 6). These data suggest that one source of G-G-anhM is chitobiose, and we would note that in a tick the only source of chitobiose would be the tick itself.

### Peptidoglycan defects in the absence of chitobiose

**Fig. 4:**
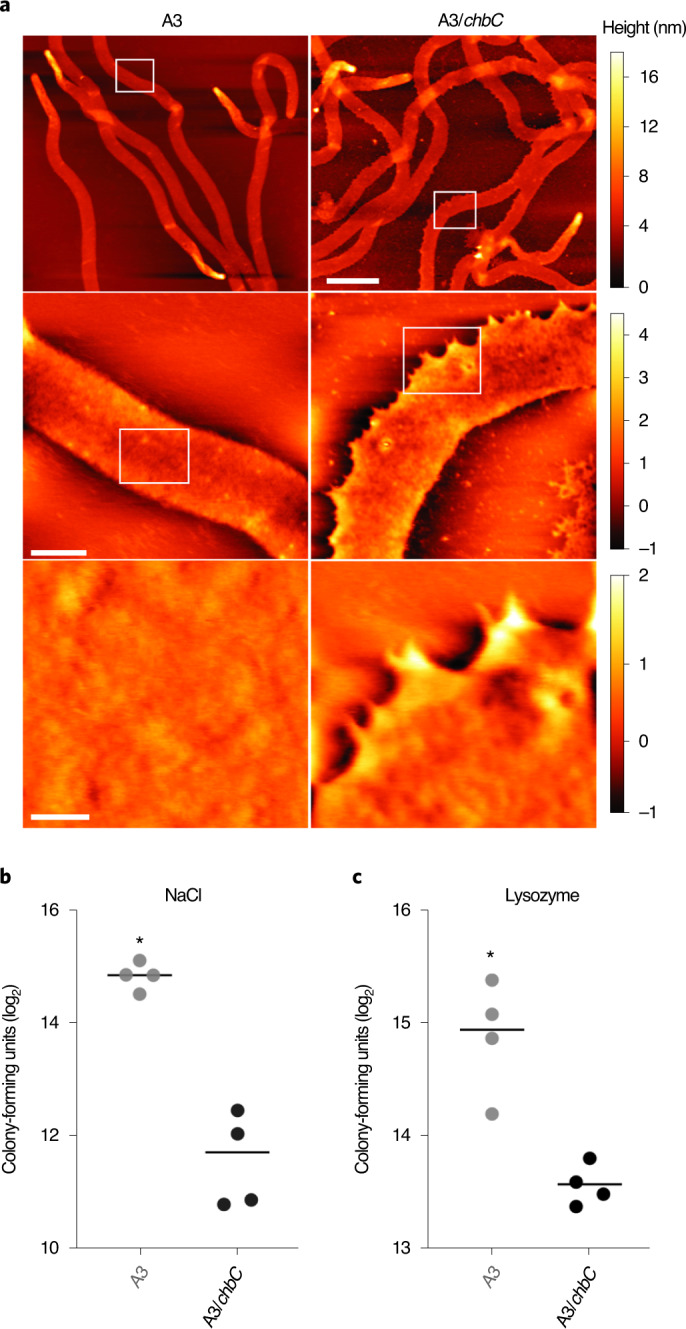
Impact of chitobiose (Glc*N*Ac–Glc*N*Ac) transport on peptidoglycan and cell-wall stress. **a**, Comparative AFM analysis of purified peptidoglycan. Peptidoglycan from both the WT A3 strain and the A3/*chbC* chitobiose mutant transporter strain was deposited on mica and topological features imaged in AM–FM mode. Height features for each image are shown as colour maps (right) in nanometres. Scale bars, 5 µm (upper panel), 500 nm (middle panel) and 50 nm (lower panel). **b**,**c**, Recovery after cell-wall stress. WT A3 and A3/*chbC* strains were exposed to 0.25 M NaCl (544 mosmol) (**b**) or 1 mg ml^−1^ of lysozyme (**c**) for 24 h. After removing exogenous stress, cultures were plated in quadruplicate and colony-forming units were determined 9 d later. Statistical significance (^*^) was determined by two-tailed, unpaired Student’s *t*-test (NaCl: *P* = 1.37 × 10^−5^; lysozyme: *P* = 0.009). Source data

Bacteria rely on peptidoglycan as an osmoprotectant and a load-bearing structure. We hypothesized that severe phenotypes would result from reduced peptidoglycan and/or G-G-anhM. We used atomic force microscopy (AFM) to analyse purified peptidoglycan sacculi and found that A3/*chbC* peptidoglycan was jagged and frayed, compared with smooth, WT, peptidoglycan sacculi (Fig. 4a). The gross structural defects that we observed in purified peptidoglycan sacculi from A3/*chbC* led us to ascertain the phenotypes of live cells. We exposed parental WT A3 and A3/*chbC* strains to either osmotic (NaCl; Fig. 4b) or peptidoglycan-specific (lysozyme; Fig. 4c) stressors for 24 h, diluted each into medium lacking stress and plated. The parental A3 control strain produced significantly more colonies, indicating that it was able to withstand osmotic and enzymatic degradation better than the mutant (Fig. 4b,c).

### Motility and physical properties of peptidoglycan with fewer Glc*N*Ac–Glc*N*Ac disaccharides

**Fig. 5:**
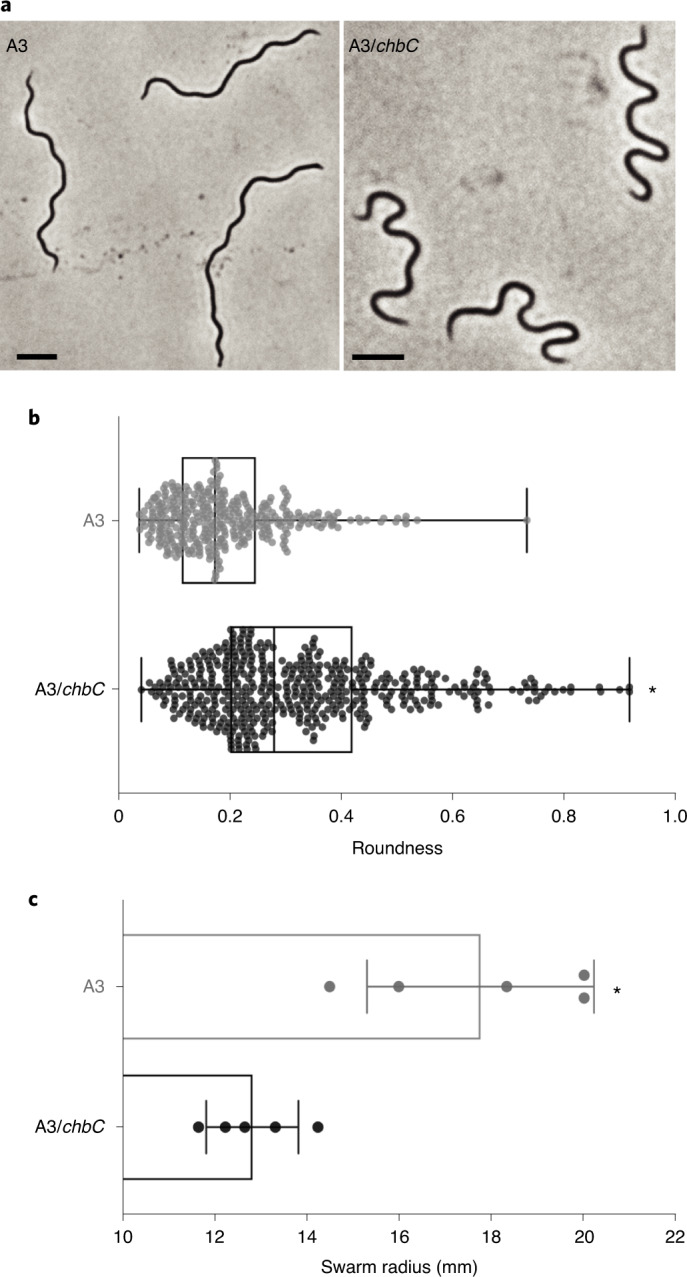
Morphological and motility defects in A3/*chbC* mutant bacteria. **a**, Comparative, quantitative, population-level morphological analysis of A3 and A3/*chbC* strains. Both strains were cultured to mid-log(exponential growth), fixed with paraformaldehyde to preserve cellular helicity and imaged on agarose pads by phase-contrast microscopy. Scale bar, 5 µm. **b**, Morphometric, population-level analysis of differences in helical pitch between strains estimated by the object analysis feature Roundness. Box plots from values attained from *n* = 360 (A3) and *n* = 481 (A3/*chbC*) strains are shown. Each dot indicates the values attained from an individual cell. Statistical significance was determined using the two-tailed, unpaired Student’s *t*-test (*P* = 6.67 × 10^−9^). **c**, Swarm plate assay to measure differences in bacterial motility. Liquid A3 and A3/*chbC* cultures were enumerated and equal amounts used to inoculate the same semisoft agar plate, equidistant from each other. After 5 d, swarming distance was measured from five replicate plates. Statistical significance (^*^) was determined using the two-tailed, unpaired Student’s *t*-test (*P* = 0.0031). Source data

One distinguishing feature of *Borrelia* spp. is periplasmic flagella. An individual flagellum wraps around the cell cylinder and peptidoglycan layer to impart a ‘flat-wave’ morphology^23^. Each flagellum is inserted into 7–11 motors^24^, which are positioned adjacent to each cell pole. Motor rotation of the flagella produces huge torsional stress, which creates backward moving waves that propel the organism forwards. Theoretically, contorting the cell cylinder with torque of this magnitude would necessitate strong and flexible peptidoglycan to counteract the deforming forces produced by the flagella^25,26^. We speculated that defects in peptidoglycan caused by a reduction in G-G-anhM might alter the response to flagellar ribbon tension, thereby resulting in altered morphology. Phase-contrast micrographs of individual cells show a clear discrepancy in the pitch (or trough) of the wave between WT A3 and A3/*chbC* strains (Fig. 5a). Morphometric, single-cell analysis between each population was determined by measuring the Roundness^27^ or the collective area required to enclose an object in an ellipse, corrected by aspect ratio^28,29^. Roundness provides a normalized assessment of deviations from the typical flat-wave morphology by estimating collective differences in helical trough depth. Population-level analysis of individual cells confirmed that there was a significant amount of variability in the helical pitch of the chitobiose mutant strain (Fig. 5b). Morphological changes in helicity suggest an imbalance in the elastic force homoeostasis between the peptidoglycan and the motility machinery.

An imbalance in counteracting forces may impact spirochaete motility. We evaluated this possibility by a swarm assay, in which two equidistant sites on a single semisoft agar plate were inoculated with each strain and, after 5 d of incubation, the radial distance was measured. Although A3/*chbC* retains the ability to move in this assay, the A3 WT strain translated significantly greater distances, confirming that cell wall:motility balance was disrupted (Fig. 5c).

**Fig. 6:**
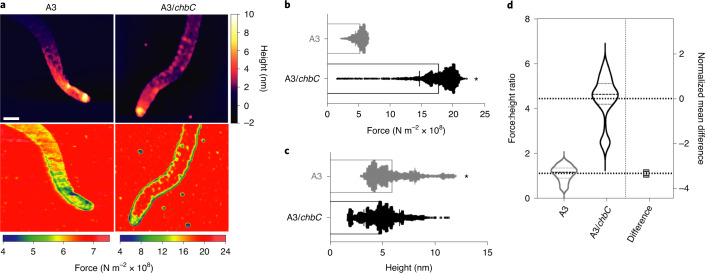
Biophysical properties of *B. burgdorferi* peptidoglycan with reduced levels of G-G-anhM. **a**, AM–FM topological mapping (upper) and elasticity measurements (lower) using the Hertz contact model on purified peptidoglycan sacculi from each strain. Note that measurements collected for each sample had dramatically different force ranges, which is reflected in colour maps (below). These images represent data collected from eight independent sacculi per sample. Scale bar, 400 nm. **b**, Line-scan analysis of force measurements collected from each pixel in seven independent sacculi per sample. Statistical significance (^*^) was assessed using the two-tailed, unpaired Student’s *t-*test (*P* = 5.3 × 10^−5^). **c**, The same line scans in **b** were used to measure pixel-level height differences in each sample. Statistical significance (^*^) was assessed using the two-tailed, unpaired Student’s *t-*test (*P* = 0.0028). **d**, Fold-change of the elasticity of A3/*chbC* peptidoglycan, relative to A3, normalized by peptidoglycan height.

These data lend support to a model in which *B. burgdorferi* peptidoglycan homoeostasis is tuned to the torsional stress created by periplasmic flagella^30^. Our analyses provide evidence that the *B. burgdorferi* cell-wall composition is required to withstand the torsional forces produced by periplasmic endoflagella. The phenotypic differences (Figs. 4 and 5) we observed in bacteria unable to import chitobiose might result solely from reduced levels of cellular peptidoglycan (Fig. 3b), or it is possible that reduced levels of G-G-anhM could alter the biophysical properties of the *B. burgdorferi* peptidoglycan sacculus. To evaluate whether the incorporation of Glc*N*Ac–Glc*N*Ac into *B. burgdorferi* peptidoglycan increases the distance between muropeptides, adjacent to glycan termini (Fig. 2 and Extended Data Fig. 2), and renders peptidoglycan more flexible, we carried out elasticity-based mechanical measurements using AFM on purified sacculi. Comparative analysis of peptidoglycan elasticity between individual sacculi appeared similar (Fig. 6a) but, to capture the full range of measurements, the A3/*chbC* sample required a colour map that extended >3× the maximum force of WT sacculi (Fig. 6a). Tandem height and force-map measurements revealed that peptidoglycan samples with reduced G-G-anhM were, on average, 3.3× stiffer than those with WT levels of G-G-anhM (Fig. 6b). We note, however, that topological height mapping also showed differences in peptidoglycan thickness between samples (Fig. 6c), potentially due to less total peptidoglycan in A3/*chbC* (Fig. 3b). To exclude the possibility that differences in thickness contribute to elastic modulus differences, we normalized readouts to peptidoglycan thickness, and performed a relative comparison on the same sacculi. Even after accounting for thickness differences, G-G-anhM content correlated with elasticity (Fig. 6d).

## Discussion

Peptidoglycan is important in bacterial physiology, morphology, cell biology, host interactions, and as a target for antibiotics^1,31,32^. Peptidoglycan cell-wall chemistry is intimately linked to each process, but typically by way of the variability in muropeptide(s) and/or their linkages. Peptidoglycan glycan stoichiometry, on the other hand, was thought to be invariable. In the present study, we report that peptidoglycan from multiple strains and species of *Borrelia* terminates glycans with G-G-anhM (Fig. 2d).

Peptidoglycan determines the shape of most bacterial cells through its flexibility and structure^33,34^. However, in *B. burgdorferi*, the periplasmic flagellar ribbon is the main cell-shape determinant^23,24^. Modelling has indicated that peptidoglycan resists both the natural curvature of the flagellar filaments and the stress created by locomotion^25,35^. We provide evidence for this model. Specifically, we demonstrate that bacteria unable to import chitobiose have reduced amounts of peptidoglycan (Fig. 3) and altered peptidoglycan composition (Fig. 4), which results in abnormalities in cell morphology (Fig. 5a,b).

Disrupting the balance between flagellar motion and peptidoglycan structure impairs motility (Fig. 5c). The elastic properties of peptidoglycan are a function of the degree and type of peptide cross-linking, in addition to the thickness and glycan orientation relative to the long axis^33,36–38^. Our AFM analyses suggest that a *B. burgdorferi* peptidoglycan structure might endow cells with elasticity by terminating glycans with G-G-anhM (Figs. 1 and 2), which enables larger distances between adjacent peptides and increases peptidoglycan flexibility (Fig. 6). The latter is remarkable given that, on average, *B. burgdorferi* glycan length is 30 disaccharides^7^ and, thus, G-G-anhM can constitute only ~3.3% of all muropeptides (Supplementary Tables 1–3). Although it is difficult to make direct comparisons of elastic modulus results due to the variability in probe standardization and data acquisition, our Young’s modulus measurements of WT *B. burgdorferi* peptidoglycan (~5.2 × 10^8^ N m^−2^; Fig. 6b) are very much in line with those published for *E. coli* (~3.5 × 10^8^ N m^−2^)^36^, which were obtained using similar sample processing and data acquisition conditions. The elastic properties of peptidoglycan isolated from bacteria unable to utilize chitobiose—leading to a reduction in G-G-anhM—were reduced, resulting in stiffer peptidoglycan (Fig. 6). Our data support the hypothesis that the flexibility and molecular organization of the *B. burgdorferi* cell wall are fine-tuned to the shape-determining properties of periplasmic flagella to enable optimal motility^38^.

The Lyme disease spirochaete lives in two distinct environments: vertebrates and ticks^39^. The *chbC* transcript is expressed during all phases of growth^40^, and is upregulated in the tick vector^41^ and under conditions similar to the tick midgut^13^, when spirochaete replication rate is slow^42^ and sugar metabolism is at a premium. The tick-associated, *B. burgdorferi* response regulator Rrp1 is involved in *chbC* upregulation in the vector, probably via RpoS^15^. Chitobiose is thought to be important both in cell-wall biosynthesis and as a carbon/nitrogen source in the nutrient-poor tick midgut, but through the utilization and isomerization of GlcNAc monomers^13,15,23^, not the direct use of the disaccharide chitobiose in peptidoglycan biosynthesis (Fig. 3). It is surprising that chitobiose transport is not required to successfully complete the tick–vertebrate enzootic life cycle of *B. burgdorferi*^21^. Chitobiose transport accounts for only ~37% of peptidoglycan G-G-anhM (Supplementary Table 6), which means that *B. burgdorferi* must possess additional, yet to be determined, means by which G-G-anhM is synthesized.

*B. burgdorferi* encounters transient changes in osmotic stress during migration from the tick midgut to the salivary glands during feeding and subsequently in a vertebrate host^43^. Bacteria with reduced *chbC* synthesis cannot survive in medium with >500 mosmol^43^, which is in line with our findings (Fig. 4b). Curiously, early stages of *B. burgdorferi* migration in the tick are reported to coincide with changes in spirochaete morphology and mode of motility^44^. It is possible that, similar to other pathogens that alter their peptide cross-linking to withstand changes in environmental and host-derived insults^45^, *B. burgdorferi* alters the amount of G-G-anhM in its cell wall during different stages of the enzootic cycle.

Bacterial growth requires peptidoglycan turnover. Fragments are excised from the existing sacculus and replaced with large multimers, resulting in elongation. Instead of re-purposing released muropeptides, like many diderms *B. burgdorferi* sheds them into their environment^7^. The hallmark of muropeptide turnover is the release of anhMur*N*Ac-containing peptidoglycan fragments^46^. It is tempting to speculate that G-G-anhM may be key in peptidoglycan-associated Lyme disease pathologies^7^. Not only may G-G-anhM-containing muropeptides produce unusual innate immune-mediated responses, but also they may be responsible for creating specificity in certain surveillance system(s)^47^. The unusual sugar organization may also be more resistant to degradation (Fig. 4c) by host-derived lysozyme and could be key in extending the half-life of *B. burgdorferi* peptidoglycan in the synovial fluid of patients with Lyme disease arthritis^7^.

The evolutionary landscape of arthropods, and their resident microbial symbionts, is beginning to come into focus. Co-evolutionary adaptive mechanisms have been fine-tuned for tens of millions of years^48^. For instance, *I. scapularis* has co-opted a peptidoglycan hydrolase of bacterial origins to limit *B. burgdorferi* expansion^49^, while protecting itself from pathogen acquisition. Microbial communities act in concert to alter tick midgut physiology, impacting the frequency and transmissibility of its residents^50,51^. *B. burgdorferi* has foregone the need for seemingly essential vitamins like thiamine, which are probably not present in tick midguts^52^. Our findings provide another example of how an endoparasitic bacterium has evolved to hijack arthropod components for use as a basic cell-wall building block.

## Methods

### *B. burgdorferi* strains, genome analysis, growth conditions and analysis

All *B. burgdorferi* strains used in the present study are transformable derivatives of the type strain^10^. *B. burgdorferi* B31-5A11, B31-5A3 and a non-infectious clone of B31-5A3 (ref. ^53^) were provided by F. Gheradini (National Institutes of Health (NIH)), J. Coburn (Medical College of Wisconsin) and U. Pal (University of Maryland), respectively. The B31-5A3/*chbC1* strain was provided by P. Rosa (Rocky Mountain Labs, NIH) and has been characterized elsewhere^21^. *B. hermsii* strain HS1 was purchased from American Type Culture Collection (ATCC).

All *Borrelia* strains were grown in Barbour–Stoenner–Kelly II (BSK-II) medium supplemented with 6% heat-inactivated rabbit serum (Gibco Laboratories), hereafter referred to as BSK-II complete culture medium^54^. We note that BSK-II complete culture medium contains yeast autolysate, which is a source of chitobiose (Extended Data Figs. 3 and 4). Metabolic labelling studies simply replaced unlabelled Glc*N*Ac (Sigma-Aldrich, 0.33 g l^−1^) with [1-^13^C]Glc*N*Ac or [1-^13^C]Man*N*Ac (Omicron Biochemicals). Glc*N*Ac-free BSK-II was supplemented with varying amounts of Gal*NA*c or Man*N*Ac (Sigma-Aldrich), as described in the text. Regardless of medium manipulations, all cultures were incubated at 37 °C with 5% CO_2_. Bacteria were enumerated using Incyto C-Chip disposable haemocytometers (SKC Inc.). All measurements were performed in triplicate and the average was reported or used to normalize material for downstream analysis.

The entire genome of each *B. burgdorferi* strain was sequenced to confirm: (1) plasmid content; (2) clean deletion of A3/*chbC*; (3) A3/*chbC* free of polar mutations; and (4) clonality (Supplementary Tables 4 and 5). We note that cells collected for DNA analysis were from a small fraction of a larger batch of culture that was used for peptidoglycan analysis and, thus, were the same passage. For instance, 450 ml of batch culture was split into 40 ml and 410 ml before harvesting cells by centrifugation. After washing each 3× with phosphate-buffered saline (PBS), genomic DNA was purified using quick-DNA miniprep plus kit (Zymo Research) following the manufacturer’s recommended procedures for the 40-ml culture, whereas the rest was used to attain a highly pure preparation of peptidoglycan (below). Purified DNA was sequenced and assembled by the Microbial Genome Sequencing Center. Reads were analysed using breseq^55^ (freely available online at http://barricklab.org/breseq) to align Illumina reads with reference genome. We ran breseq separately for each of three strains to identify base-pair substitutions and plasmid profiles relative to the reference genome B. *burgdorferi* B31 clonal isolate 5A3 (RefSeq GCF_000008685.2). Outputs were analysed manually and summarized in Supplementary Tables 4 and 5.

For peptidoglycan purification, cells were harvested when cultures reached a density of ~5 × 10^7^ cells ml^−1^ by centrifugation at 3,500*g* for 15 min at 4 °C. The resulting pellet was gently washed 3× with PBS before being centrifuged at 3,000*g* for 15 min at 4 °C. Whole-cell lysate pellets were stored at −20 °C for later use. For direct comparative purposes (A3 versus A3/*chbC*), cells were enumerated and peptidoglycan was extracted (below) from equivalent cell counts (5 × 10^7^ cells ml^−1^).

### Peptidoglycan isolation: intact peptidoglycan sacculi

Peptidoglycan was isolated and purified from 0.25–2 l of mid-log phase cultures; volumes depended on application. Regardless of culture volume, all peptidoglycan was prepared following previously published procedures^7,56^. The final pellet, containing intact peptidoglycan sacculi, was resuspended in 495 µl of ultra-pure H_2_O. Intact peptidoglycan sacculi were stored at 4 °C for AFM analysis or used to generate digested muropeptides as described in the following sections.

### Peptidoglycan processing for muropeptide analysis

Intact peptidoglycan sacculi, resuspended in NaHPO_4_/NaH_2_PO_4_ buffer (5 mM, pH 5.5) containing mutanolysin (7.8 µl, 4000 U ml^−1^; Sigma-Aldrich), were digested overnight at 37 °C with shaking. The following morning, an additional 7.8 µl of mutanolysin was spiked in and allowed to incubate, shaking, for 5 h at 37 °C. The mutanolysin digest was then heat inactivated at 100 °C for 10 min. After heat inactivation, the digest was cooled to room temperature and centrifuged at 22,000*g* for 30 min. The supernatant (containing digested peptidoglycan muropeptides) was carefully moved to a preweighed microfuge tube without disturbing the pellet (undigested peptidoglycan). The supernatant, containing digested muropeptides, was dried and the final weight determined.

Purified, dried muropeptides were fully dissolved in 150 µl of saturated sodium borate buffer, pH 9.25. Sodium borohydride or borodeuteride (50 mg) was dissolved in 500 µl of LC–MS-grade H_2_O, and an aliquot (50 µl) was added slowly to the muropeptide solution with mixing after the addition was complete. The reduction was quenched after 1 h by the addition of LC–MS-grade formic acid (~10 µl) to a pH of ~3 as evaluated by pH paper. Samples were then immediately snap-frozen and dried using a high vacuum line equipped with a liquid nitrogen solvent trap. Dried samples were stored desiccated until analysis, which involved reconstitution in H_2_O:MeCN (200 µl, 9:1, v:v) containing 0.1% formic acid. Reconstituted samples were sonicated in a water bath for 10 min and centrifuged at 4 °C (13,000*g*, 10 min). From the centrifuged sample, 180 µl was placed in a labelled LC–MS vial for analysis.

### LC–MS analysis

Analyses were performed on a Shimadzu LCMS9030 QToF instrument interfaced with a LC-40B X3 UPLC, a SIL-40C X3 autosampler (10 °C) and a CTO-40C column oven (40 °C). Gradient separations utilized a BEH C_18_ column (2.1 mm × 50 mm, 1.7-μm particle size; Waters) with solvent A (0.1% formic acid in water) and solvent B (0.1% formic acid in MeOH) at a constant flow rate of 0.4 ml min^−1^. Initial solvent conditions were 99:1 (A:B) which was held constant for 3 min, followed by a shallow linear gradient to 8% B at 12 min, then to 20% B at 24 min and finally to 95% B at 25 min, which was held for 4 min. The gradient was converted to starting conditions with a 1-min gradient to 1% B (29 min), followed by a 5-min hold. Sample injection volumes ranged from 0.5 μl to 20 μl. The first 1.25 min of the separation was diverted to waste to avoid reduction reaction product contamination of the mass spectrometer interface.

The mass spectrometer was operated in positive ion mode using electrospray ionization and external calibration (NaI). Interface voltage was 4.0 kV at 300 °C, with a desolvation temperature of 526 °C and a DL transfer line temperature of 250 °C. Gas flows (l min^−1^) were 2, 10 and 10 for nebulizing, heating and drying gases, respectively. Muropeptide data were collected between 1.25 and 24 min using several different MS and MS/MS programmes. For statistical comparisons of strains, data were collected in MS mode only, from 400 *m/z* to 2,000 *m/z* at 0.1 Hz. Fragmentation data were collected in data-dependent mode (top three) at low Q1 resolution, with three MS/MS spectra, before placement on the exclusion list (15 s of exclusion time). The precursor window as set to 400–2,000 *m/z* with fragmentation data collected between 50 and 2,000 *m/z*, using a ramped collision energy (25 ± 10 V). Total duty cycle was 0.4 s (0.1 s per event).

### LC–MS data analytics

Shimadzu.LCD files were converted to the .mzML file format using Shimadzu LabSolutions (v.5.99 SP2). The discovery of features and associated peak areas was performed using the xcms package (v.3.13) in the R programming environment (v.4.0.3)^57,58^. The R package RamClustR (v.1.1)^59^ was used to reduce spectral redundancy through the binning of the features into groups and this reduced dataset was used for further statistical analysis. Statistical analysis was performed using MetaboAnalyst 4.0 (ref. ^60^). Principal component analysis was performed on log(transformed) and pareto-scaled peak area values.

To determine the relative amount of G-G-anhM present in WT A3 and A3/*chbC* strains, we prepared 6 independent, 450-ml culture volumes of BSK-II complete medium. Each strain was propagated in three independent cultures and peptidoglycan was purified from the same number of cells, in all six samples. Relative abundances of muropeptides were quantified from all three independent peptidoglycan samples of A3 and A3/*chbC*. RamClustR was once again used as above; however, from the resulting binned dataset, data were manually curated to ensure that all adducts and redundancies were successfully filtered out. For each muropeptide, from each replicate, relative abundance was calculated as the amount of muropeptide compared with the sum of all muropeptides present. These values were averaged between all three replicates of A3/*chbC* and compared with the averaged replicates of A3 for each muropeptide.

### Confirmation of chitobiose in autohydrolysed yeast

The confirmation is based on matching retention times and high-resolution mass spectrometric analysis of both parent and fragment ions. Both a chitobiose standard (Neogen) and autohydrolysed yeast (Yeastolate, Difco, BD & Co.) were separated by porous graphitic carbon (PGC) LC, essentially as described previously^61^. Separations were performed on a Hypercarb PGC column (100 mm × 2.1 mm, 5-mm particle size; Thermo Fisher Scientific) using a binary gradient of water (solvent A) and acetonitrile (solvent B), both containing 10 mM ammonium hydroxide. The separation began at 95% solvent A (0–2 min), with a linear gradient to 75% A at 15 min and then to 5% A at 20 min. The system was held at 5% A for 4 min, with a 1-min linear ramp back to initial conditions, and held for 5 min. Total run time was 30 min at a flow rate of 0.4 ml min^−1^, with the column maintained at 50 °C. The LC unit comprised two LC-40B X3 pumps, a SIL-40C X3 autosampler (10 °C) and a CTO-40C column oven (Shimadzu Scientific). The first 1.25 min of the separation was sent to waste, with data collection from 1.25 min to 24 min.

The mass spectrometer (LCMS9030; Shimadzu) was operated as described for the muropeptide work using three events. Event 1 was a scan from 200 *m/z*, to 1,500 *m/z*, followed by two sequential MS/MS scans using *m/z* values matching those of the [M + H]^+^ and [M + Na]^+^ forms of *N,N*′-diacetylchitobiose (425.1766 and 447.1585). Each event time was 0.1 s. Collision energy for the MS/MS scans was ramped ±17 V centred on 35 V.

### Monosaccharide analysis

Peptidoglycan glycosyl composition analysis was performed by the Complex Carbohydrate Research Center (Athens, GA). Peptidoglycan was purified, as described above, from two independent *E. coli* K-12 and *B. burgdorferi* 5A11 cultures. Each sample was spiked with 20 µg of myoinositol (internal standard) and hydrolysed (200 μl 6 M HCl, 100 °C, 16 h). After solvent removal under a stream of nitrogen, glycosyl composition analysis was performed by combined gas chromatography (GC)–MS of the alditol acetates as described previously^62^. The samples were hydrolysed again in 2 M trifluoroacetic acid for 2 h in a sealed tube at 120 °C, reduced with NaBD_4_ and acetylated using acetic anhydride/pyridine. The resulting alditol acetates were analysed by GC–MS analysis on an Agilent 7890A GC interfaced to a 5975C MSD, electron impact ionization mode. Separation was performed on a 30-m Equity 1 capillary column. Alongside the samples, standards of Glc*N*Ac, Gal*N*Ac, Man*N*Ac and Mur*N*Ac were also analysed.

### NMR spectroscopy

Muropeptide samples and a chitotriose standard were reduced with NaBH_4_ as described above, followed by removal of reaction byproducts using gravity-fed size exclusion chromatography (1 cm × 20 cm column, 15 ml of Bio-Gel P-2 medium, fine-grade) using a 9:1 (v:v) mixture of water:95% ethanol (food grade/glass distilled) as the mobile phase. Muropeptide fractions (~0.5 ml) were collected manually and combined after assessment by ultraviolet absorption (DeNovix DS-11 FX+) and LC–MS. Combined fractions were snap-frozen, dried and freeze-dried once with 100% D_2_O before NMR. Samples (unlabelled muropeptides, ^13^C-labelled and chitotriitol) were dissolved in 100% D_2_O, placed in a standard NMR tube (unlabelled and chitotriitol) or a Shigemi tube (^13^C-labelled) and analysed on a Bruker Biospin600 MHz instrument. Standard pulse sequences were used for ^1^H, ^13^C, COSY, gH2BC, gHMBC and gHSOC. Data were processed using MestReNova (v.14.2, Mestrelab Research).

### Stress tests and plate recovery assay

WT 5A3 and 5A3/*chbC* strains were cultured to a final density of 5 × 10^7^ cells ml^−1^ and back-diluted to a concentration of 10^6^ cells ml^−1^ in 5 ml in BSK-II complete culture medium. NaCl (Affymetrix) and lysozyme (Sigma-Aldrich) were added to a final concentration of 0.1 M and 0.375 µg ml^−1^, respectively—one treatment per tube, per strain—and incubated for 24 h at 37 °C and 5% CO_2_. These conditions are identical to those used previously for a similar purpose^8^. We note that the addition of 0.1 M NaCl resulted in a final osmolality of 544 mosmol, as determined by Fiske Micro-Osmometer Model 210, following the manufacturer’s recommended procedure.

Four batches of 100-ml BSK plating medium were prepared as previously described^8^ and added to four 100-ml volumes of pre-equilibrated, 5% low-melt agarose solution (1:1 ratio). The resulting solution was then allowed to re-equilibrate to 48 °C in the water bath (referred to as the plating medium hereafter). The plating medium was poured—25 ml per plate, 4 plates per batch of plating medium, for 16 plates—and allowed to solidify at room temperature for 2 h. The medium poured constitutes the bottom layer. The top layer consisted of an equal amount of culture medium, which was inoculated in a serial dilution of each strain, for each treatment. After 9 d, the colony-forming units were determined using a magnified Petri dish light box, fitted with a grid.

The serial dilution replicates for each strain and treatment were normalized by cell inoculum concentration, to the highest concentration, and reported as total colony-forming units observed. Differences in total colony-forming units were compared using a two-tailed, unpaired Student’s *t*-test and graphed in GraphPad Prism 8.0.

### Swarm/motility assay

To evaluate the motility of 5A3 and 5A3/*chbC* strains, we prepared solid medium as described above, but with the following modifications: (1) the final concentration of low-melt agarose was adjusted to 0.5% (w:v) and (2) the entire volume-plating medium was added at once (that is, no layering) and allowed to solidify for 4 h at room temperature. After the plates solidified, we subsurface inoculated one side of each plate with 7.5 µl of the 10^9^ cells ml^−1^ of 5A3, and the other with equal amounts of 5A3/*chbC*. This was repeated for a total of five plates.

After 5 d we measured the radius, in millimetres, of the disseminated colony in four different directions. These values were averaged to obtain a single, average radius value, which was recorded for both strains on each plate. Differences in the five average radius values for 5A3 and 5A3/*chbC* were compared using a two-tailed, unpaired Student’s *t*-test and graphed in GraphPad Prism 8.0.

### Sample preparation, brightfield microscopy and image analysis

The morphological differences between 5A3 and 5A3/*chbC* were evaluated using phase-contrast microscopy on fixed cells. Briefly, both strains were cultured to a final density of 10^7^ cells ml^−1^ in BSK-II complete culture medium. Cells were fixed by adding 16% paraformaldehyde, from a fresh ampoule to a final concentration of 1.8% (v:v), as previously described^63^. The mixture was incubated with gentle agitation for 10 min at room temperature, followed by 20 min on ice. Fixed cells were harvested by centrifugation at 3,500*g* for 15 min at 4 °C, and washed 3× with, and resuspended in, PBS.

Fixed cells were spotted on 2% agarose (in PBS) pads, as previously described^56^. Phase-contrast micrographs were acquired on a Zeiss Axio Observer equipped with an oil-immersion phase-contrast objective Plan Apochromat 100×/1.45 numerical aperture (Nikon) using a Hamamatsu Orca-Flash 4.0 V3 Digital CMOS camera. Image acquisition occurred on the same day, using the same agarose pad, which was split in half. Cell preparation, image acquisition and analysis were repeated to ensure reproducibility. Results from independent experiments were almost identical and, thus, results from one experiment were reported.

We attempted to use the automated cell detection software Oufti^64^, as has been done in the past for *B. burgdorferi* phase-contrast micrographs^56^. However, the gross morphological changes (Fig. 5) in 5A3/*chbC* made cell detection challenging. We opted for an alternative approach whereby a threshold was applied to each phase-contrast micrograph, using Fiji. This resulted in clear cell outlines, with clean cell boundaries, for virtually all cells in a field of view (see Supplementary Fig. 32, for example). After semi-automated cell detection, we used the macro function Roundness to calculate differences in cell shape. In the present study, the cell area is fitted to an ellipse, normalized by the aspect ratio of the object—an established method to evaluate the differences in the area that a cell occupies^27^. Values were attained from ≥300 cells for each experiment and statistical significance was determined by an unpaired Student’s *t-*test.

### AFM

A suspension of purified peptidoglycan (above), isolated from 5A3 and 5A3/*chbC*, was created with ultra-pure water, diluted 1:5 (v:v), and 50 μl was deposited on to a freshly cleaved mica sheet (10 mm in diameter) attached to a metal AFM sample puck with epoxy. Samples were incubated for 5 min before being dried with nitrogen gas. All images were acquired using a Jupiter-XR AFM (Oxford Instruments Asylum Research) operating in amplitude-modulated–frequency-modulated (AM–FM) mode with an AC160TS-R3 (Olympus) cantilever. Cantilever oscillation was produced using photothermal excitation. The cantilevers first Eigen and second Eigen modes were tuned to free amplitudes of 2 and 0.025 V, respectively. The setpoints were established to achieve a phase angle <90° (repulsive regime) to permit stiffness image acquisition: typically, 1.5 and 0.018 V, respectively. Stiffness values were calculated using the Hertz contact model assuming that the radius of contact was 8 nm. Before image acquisition, the cantilever spring constant was calibrated using Asylum Research’s GetReal Calibration Software API. Raw data files were processed and analysed using Gwyddion. Height and stiffness measurements were compiled in Gwyddion and results graphed using GraphPad Prism 8.0.

### Reporting Summary

Further information on research design is available in the Nature Research Reporting Summary linked to this article.

## Supplementary information

Supplementary Information Supplementary Figs. 1–32, Supplementary Tables 1–6.

Reporting Summary

Supplementary Data 1 AFM height and force measurements associated with panels in Fig. 6.

Peer Review Information

## Data Availability

All data collected from our studies can be found in the main article, Supplementary information, Extended data and Source data. The raw WGS data can be found here: strain B31-5A11, accession no. SAMN21566060 (https://www.ncbi.nlm.nih.gov/biosample/SAMN21566060; strain B31-5A3, accession no. SAMN21566061 (https://www.ncbi.nlm.nih.gov/biosample/21566061); strain B31-5A3n.i, accession no. SAMN21566062 (https://www.ncbi.nlm.nih.gov/biosample/21566062); strain B31-5A3/*chbC*, accession no. SAMN21566063 (https://www.ncbi.nlm.nih.gov/biosample/21566063). Source data are provided with this paper.

## Source data

Source Data Fig. 3 RamClustR analysis of MS features in Fig. 3.
Source Data Fig. 4 Colony-forming units after NaCl and lysozyme stress in Fig. 4.
Source Data Fig. 5 Morphological and motility analysis in Fig. 5.

## Extended data

are available for this paper at 10.1038/s41564-021-01003-w.
