# Supplementary material for: The unusual cell wall of the Lyme disease spirochaete Borrelia burgdorferi is shaped by a tick sugar
Source: Nat Microbiol. 2021 Nov 24;6(12):1583–92. doi: 10.1038/s41564-021-01003-w (PMC8612929; doi:10.1038/s41564-021-01003-w)
Supplement: Supplementary file 1 — Supplementary Figs. 1–32, Supplementary Tables 1–6. [file 41564_2021_1003_MOESM1_ESM.pdf]

nature portfolio

https://doi.org/10.1038/s41564-021-01003-w

Supplementary information

The unusual cell wall of the Lyme disease spirochaete *Borrelia burgdorferi* is shaped by a tick sugar

In the format provided by the authors and unedited

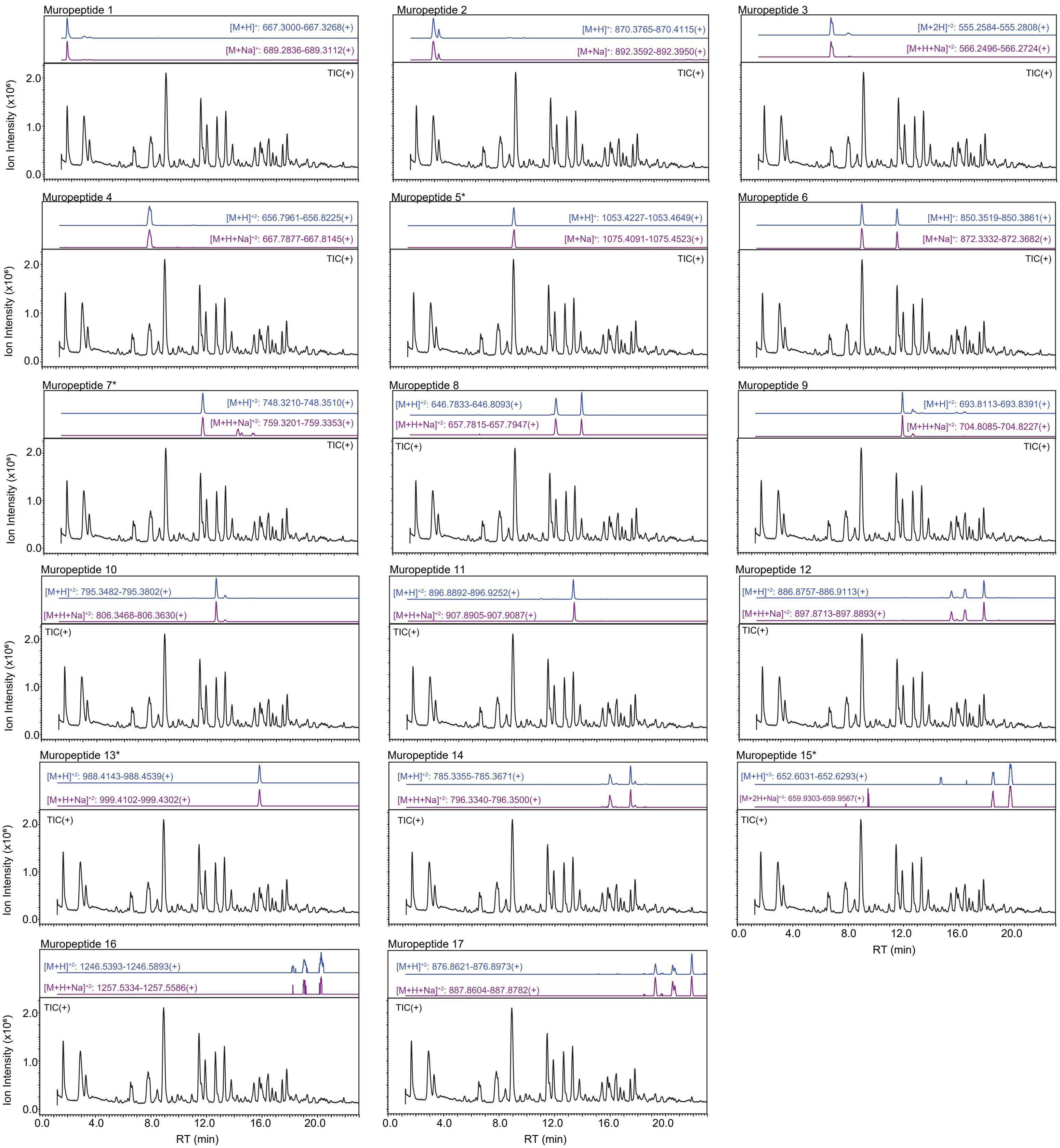

Supplemental Figure 1: LCMS chromatograms by muropeptide – Total ion counts (TICs) are shown in black with the [M +H]+ and [M+Na]+ for each muropeptide shown in blue and purple, respectively. *Muropeptides containing G-G-anhM are denoted by an asterick.

Supplemental Table 1: Analysis of muropeptides present in 5A11 *B. burgdorferi* peptidoglycan.

Muropeptide ID	Structure	Relative % of PG	Theoretical [M+H]$^+$	Previously Identified as$^{\gamma}$
1	Mur*N*Ac-Ala-Glu-Orn-Gly	16.9	667.3145	Peak 1
2	Glc*N*Ac-Mur*N*Ac-Ala-Glu-Orn-Gly	27.4	870.3938	Peak 2
3	Mur*N*Ac-Ala-Glu-Orn-[Gly]-Ala-Gly-Orn-Glu-Ala	1.7	1109.5321	NM
4	Glc*N*Ac-Mur*N*Ac-Ala-Glu-Orn-[Gly]-Ala-Gly-Orn-Glu-Ala	<0.5	1312.6114	NM
5*	Glc*N*Ac-Glc*N*Ac-Mur*N*AcAnh-Ala-Glu-Orn-Gly	0.6	1053.4470	Peak 3
6	Glc*N*Ac-Mur*N*AcAnh-Ala-Glu-Orn-Gly	5.4	850.3676	Peak 4 & 7
7*	Glc*N*Ac-Glc*N*Ac-Mur*N*AcAnh-Ala-Glu-Orn-[Gly]-Ala-Gly-Orn-Glu-Ala	<0.5	1495.6646	NM
8	Glc*N*Ac-Mur*N*AcAnh-Ala-Glu-Orn-[Gly]-Ala-Gly-Orn-Glu-Ala	<0.5	1292.5852	NM
9	Mur*N*Ac-Ala-Glu-Orn-[Gly]-Ala-Gly-Orn-Glu-Ala-Mur*N*Ac	4.7	1386.6482	Peak 6
10	Glc*N*Ac-Mur*N*Ac-Ala-Glu-Orn-[Gly]-Ala-Gly-Orn-Glu-Ala-Mur*N*Ac	3.0	1589.7276	Peak 8
11	Glc*N*Ac-Mur*N*Ac-Ala-Glu-Orn-[Gly]-Ala-Gly-Orn-Glu-Ala-Mur*N*Ac-Glc*N*Ac	11.7	1792.8070	Peak 9
12	Glc*N*Ac-Mur*N*Ac-Ala-Glu-Orn-[Gly]-Ala-Gly-Orn-Glu-Ala-Mur*N*AcAnh-Glc*N*Ac	6.0	1772.7808	Peak 10 & 11
13*	Glc*N*Ac-Glc*N*Ac-Mur*N*AcAnh-Ala-Glu-Orn-[Gly]-Ala-Gly-Orn-Glu-Ala-Mur*N*Ac-Glc*N*Ac	0.8	1975.8601	NM
14	Glc*N*Ac-Mur*N*AcAnh-Ala-Glu-Orn-[Gly]-Ala-Gly-Orn-Glu-Ala-Mur*N*Ac	1.0	1569.7014	NM
15*	Glc*N*Ac-Glc*N*Ac-Mur*N*AcAnh-Ala-Glu-Orn-Gly-Ala-Orn-[Gly]-Glu-Ala-Mur*N*AcAnh-Glc*N*Ac	<0.5	1955.8339	NM
16	Mur*N*Ac-(Ala-Glu-Orn-Gly-Ala)-Glc*N*Ac-Mur*N*Ac-(Ala-Glu-Orn-Gly-Ala)-Glc*N*Ac-Mur*N*AcAnh-(Ala-Glu-Orn-Gly)	<0.5	2492.1145	NM
17	Glc*N*Ac-Mur*N*AcAnh-Ala-Glu-Orn-[Gly]-Ala-Gly-Orn-Glu-Ala-Mur*N*AcAnh-Glc*N*Ac	<0.5	1752.7545	NM

γ – As previously identified in Jutras et. al., 2019, PNAS. NM denotes newly identified muropeptides.
* Muropeptide contains G-G-anhM.

Supplemental Table 2: Muropeptide-containing peaks from 5A11 *B. burgdorferi* peptidoglycan analyzed via LCMS.

Peak #	Muropeptide ID	$[M+H]^+$	Structure	RT (mins)
1	1	667.3145	Mur*N*Ac-Ala-Glu-Orn-Gly	1.688
2	2a	870.3939	Glc*N*Ac-Mur*N*Ac-Ala-Glu-Orn-Gly	2.935
3	2b	870.3939	Glc*N*Ac-Mur*N*Ac-Ala-Glu-Orn-Gly	3.342
4	3	1109.5321	Mur*N*Ac-Ala-Glu-Orn-[Gly]-Ala-Gly-Orn-Glu-Ala	6.548
5	4	1312.6115	Glc*N*Ac-Mur*N*Ac-Ala-Glu-Orn-[Gly]-Ala-Gly-Orn-Glu-Ala	7.842
6	5*	1053.4470	Glc*N*Ac-Glc*N*Ac-Mur*N*AcAnh-Ala-Glu-Orn-Gly	8.935
	6a	850.3676	Glc*N*Ac-Mur*N*AcAnh-Ala-Glu-Orn-Gly	
7	6b	850.3676	Glc*N*Ac-Mur*N*AcAnh-Ala-Glu-Orn-Gly	11.477
8	7*	1495.6646	Glc*N*Ac-Glc*N*Ac-Mur*N*AcAnh-Ala-Glu-Orn-[Gly]-Ala-Gly-Orn-Glu-Ala	11.627
9	8a	1292.5853	Glc*N*Ac-Mur*N*AcAnh-Ala-Glu-Orn-[Gly]-Ala-Gly-Orn-Glu-Ala	11.918
	9a	1386.6482	Mur*N*Ac-Ala-Glu-Orn-[Gly]-Ala-Gly-Orn-Glu-Ala-Mur*N*Ac	
10	9b	1386.6482	Mur*N*Ac-Ala-Glu-Orn-[Gly]-Ala-Gly-Orn-Glu-Ala-Mur*N*Ac	12.652
	10a	1589.7276	Glc*N*Ac-Mur*N*Ac-Ala-Glu-Orn-[Gly]-Ala-Gly-Orn-Glu-Ala-Mur*N*Ac	
11	10b	1589.7276	Glc*N*Ac-Mur*N*Ac-Ala-Glu-Orn-[Gly]-Ala-Gly-Orn-Glu-Ala-Mur*N*Ac	13.302
	11	1792.8070	Glc*N*Ac-Mur*N*Ac-Ala-Glu-Orn-[Gly]-Ala-Gly-Orn-Glu-Ala-Mur*N*Ac-Glc*N*Ac	
12	8b	1292.5853	Glc*N*Ac-Mur*N*AcAnh-Ala-Glu-Orn-[Gly]-Ala-Gly-Orn-Glu-Ala	13.802
13	12a	1772.7808	Glc*N*Ac-Mur*N*Ac-Ala-Glu-Orn-[Gly]-Ala-Gly-Orn-Glu-Ala-Mur*N*AcAnh-Glc*N*Ac	15.428
14	13*	1975.8601	Glc*N*Ac-Glc*N*Ac-Mur*N*AcAnh-Ala-Glu-Orn-[Gly]-Ala-Gly-Orn-Glu-Ala-Mur*N*Ac-Glc*N*Ac	15.828
15	14a	1569.7014	Glc*N*Ac-Mur*N*AcAnh-Ala-Glu-Orn-[Gly]-Ala-Gly-Orn-Glu-Ala-Mur*N*Ac	15.962
16	12b	1772.7808	Glc*N*Ac-Mur*N*Ac-Ala-Glu-Orn-[Gly]-Ala-Gly-Orn-Glu-Ala-Mur*N*AcAnh-Glc*N*Ac	16.433
17	14b	1569.7014	Glc*N*Ac-Mur*N*AcAnh-Ala-Glu-Orn-[Gly]-Ala-Gly-Orn-Glu-Ala-Mur*N*Ac	17.478
18	14c	1569.7014	Glc*N*Ac-Mur*N*AcAnh-Ala-Glu-Orn-[Gly]-Ala-Gly-Orn-Glu-Ala-Mur*N*Ac	17.803
	12c	1772.7808	Glc*N*Ac-Mur*N*Ac-Ala-Glu-Orn-[Gly]-Ala-Gly-Orn-Glu-Ala-Mur*N*AcAnh-Glc*N*Ac	
19	15a*	1955.8339	Glc*N*Ac-Glc*N*Ac-Mur*N*AcAnh-Ala-Glu-Orn-Gly-Ala-Orn-[Gly]-Glu-Ala-Mur*N*AcAnh-Glc*N*Ac	18.490
20	16a	2492.1145	Mur*N*Ac-(Ala-Glu-Orn-Gly-Ala)-Glc*N*Ac-Mur*N*Ac-(Ala-Glu-Orn-Gly-Ala)-Glc*N*Ac-Mur*N*AcAnh-(Ala-Glu-Orn-Gly)	19.043
21	17a	1752.7546	Glc*N*Ac-Mur*N*AcAnh-Ala-Glu-Orn-[Gly]-Ala-Gly-Orn-Glu-Ala-Mur*N*AcAnh-Glc*N*Ac	19.277
22	15b*	1955.8339	Glc*N*Ac-Glc*N*Ac-Mur*N*AcAnh-Ala-Glu-Orn-Gly-Ala-Orn-[Gly]-Glu-Ala-Mur*N*AcAnh-Glc*N*Ac	19.717
23	16b	2492.1145	Mur*N*Ac-(Ala-Glu-Orn-Gly-Ala)-Glc*N*Ac-Mur*N*Ac-(Ala-Glu-Orn-Gly-Ala)-Glc*N*Ac-Mur*N*AcAnh-(Ala-Glu-Orn-Gly)	20.265
24	17b	1752.7546	Glc*N*Ac-Mur*N*AcAnh-Ala-Glu-Orn-[Gly]-Ala-Gly-Orn-Glu-Ala-Mur*N*AcAnh-Glc*N*Ac	20.523
25	17c	1752.7546	Glc*N*Ac-Mur*N*AcAnh-Ala-Glu-Orn-[Gly]-Ala-Gly-Orn-Glu-Ala-Mur*N*AcAnh-Glc*N*Ac	21.905

* Muropeptide contains G-G-anhM

Supplemental Table 3: Theoretical vs. observed *m/z* for each muropeptide after mass correction using an internal standard.

**Muropeptide**	**Molecular formula**	**Observed Ion**	**Theoretical *m/z***	**Observed *m/z***	**Difference (Da)**
1	$C_{26}H_{46}N_6O_{14}$	$[M+H]^+$	667.3145	667.3115	0.0030
2	$C_{34}H_{59}N_7O_{19}$	$[M+H]^+$	870.3938	870.3939	-0.0001
3	$C_{44}H_{76}N_{12}O_{21}$	$[M+2H]^{+2}$	555.2697	555.2676	0.0021
4	$C_{52}H_{89}N_{13}O_{26}$	$[M+2H]^{+2}$	656.8094	656.8069	0.0025
5*	$C_{42}H_{68}N_8O_{23}$	$[M+H]^+$	1053.4470	1053.4425	0.0045
6	$C_{34}H_{55}N_7O_{18}$	$[M+H]^+$	850.3676	850.3639	0.0037
7*	$C_{60}H_{98}N_{14}O_{30}$	$[M+2H]^{+2}$	748.3359	748.3331	0.0028
8	$C_{52}H_{85}N_{13}O_{25}$	$[M+2H]^{+2}$	646.7963	646.7936	0.0027
9	$C_{55}H_{95}N_{13}O_{28}$	$[M+2H]^{+2}$	693.8278	693.8250	0.0028
10	$C_{63}H_{108}N_{14}O_{33}$	$[M+2H]^{+2}$	795.3674	795.3644	0.0030
11	$C_{71}H_{121}N_{15}O_{38}$	$[M+2H]^{+2}$	896.9071	896.9034	0.0037
12	$C_{71}H_{117}N_{15}O_{37}$	$[M+2H]^{+2}$	886.8940	886.8902	0.0038
13*	$C_{79}H_{130}N_{16}O_{42}$	$[M+2H]^{+2}$	988.4337	988.4299	0.0038
14	$C_{63}H_{104}N_{14}O_{32}$	$[M+2H]^{+2}$	785.3543	785.3512	0.0031
15*	$C_{79}H_{126}N_{16}O_{41}$	$[M+2H]^{+2}$	978.4206	978.4168	0.0038
16	$C_{100}H_{166}N_{22}O_{51}$	$[M+2H]^{+2}$	1246.5609	1246.5564	0.0045
17	$C_{71}H_{113}N_{15}O_{36}$	$[M+2H]^{+2}$	876.8809	876.8779	0.0030

* Muropeptide contains G-G-anhM.

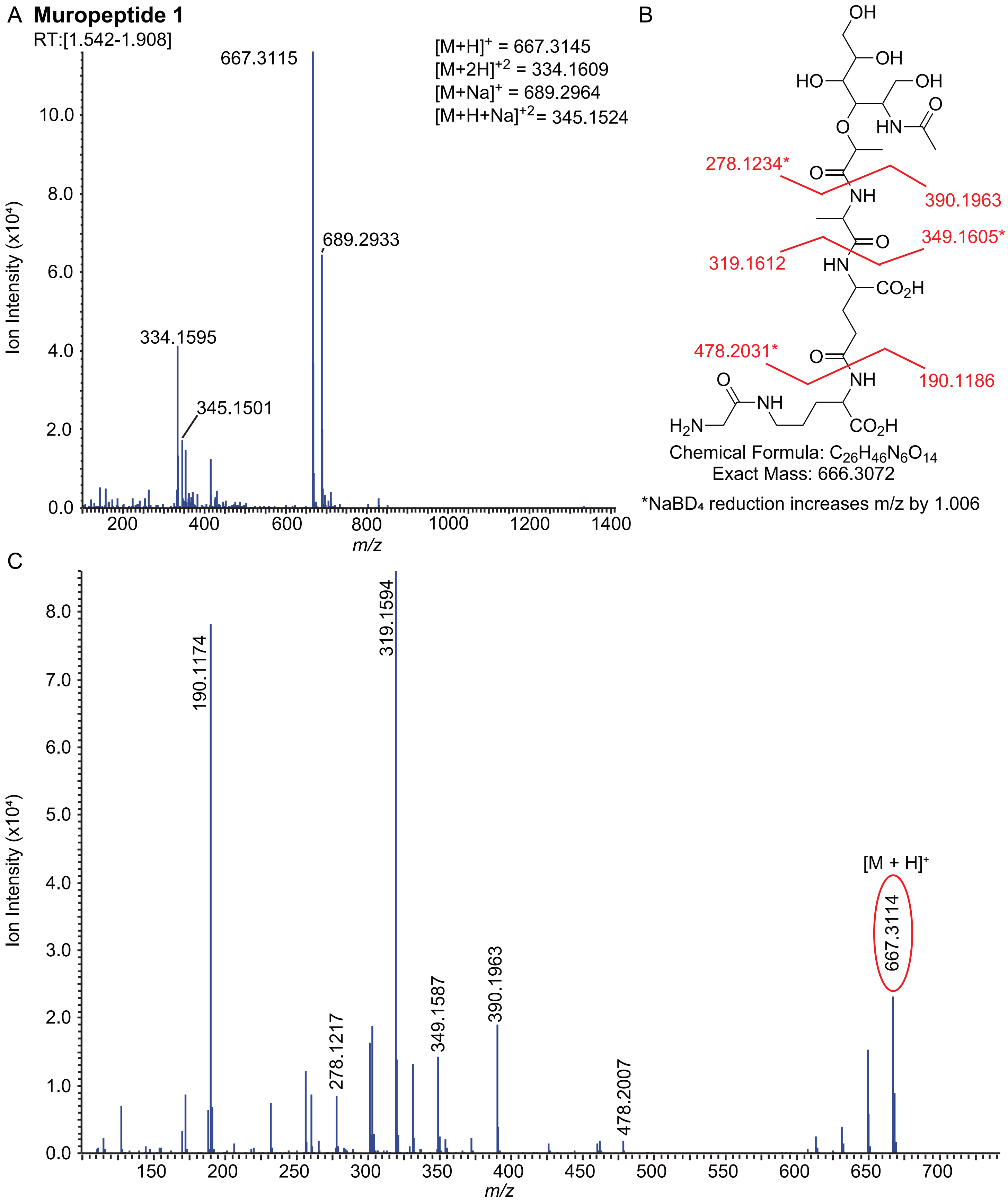

Supplemental Figure 2: (A) MS1 spectra generated from scanning retention times of 1.542-1.908 minutes. (B) The structure of muropeptide 1. Cleavages with resulting *m/z* fragments are shown in red. (C) MS2 obtained from targeting precursor ion 667.3145 $[M+H]^+$. Red fragments generated in B correspond to observed MS2 fragments in C.

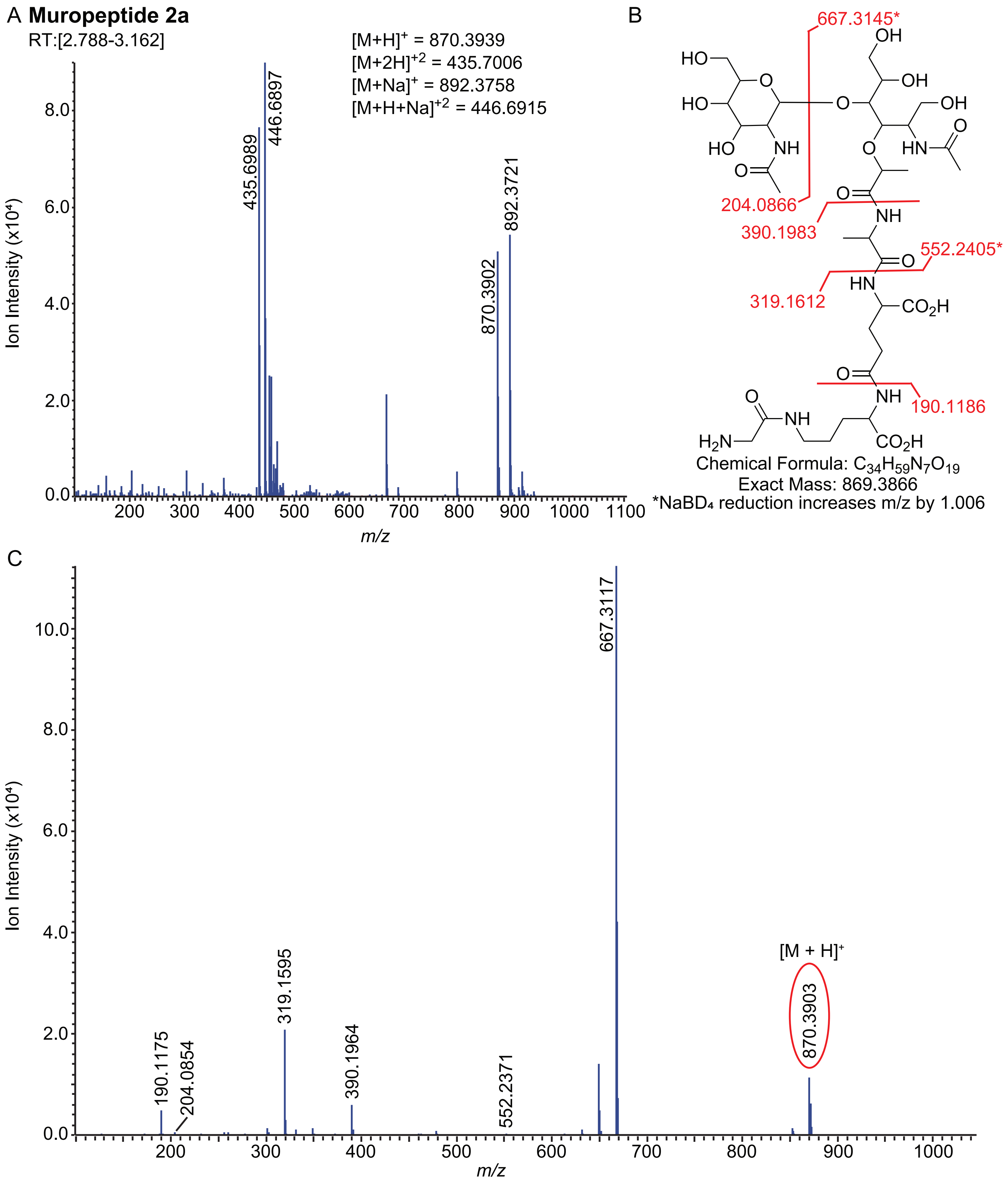

Supplemental Figure 3: (A) MS1 spectra generated from scanning retention times of 2.788-3.162 minutes. (B) The structure of muropeptide 2a. Cleavages with resulting *m/z* fragments are shown in red. (C) MS2 obtained from targeting precursor ion 870.3902 $[M+H]^+$. Red fragments generated in B correspond to observed MS2 fragments in C.

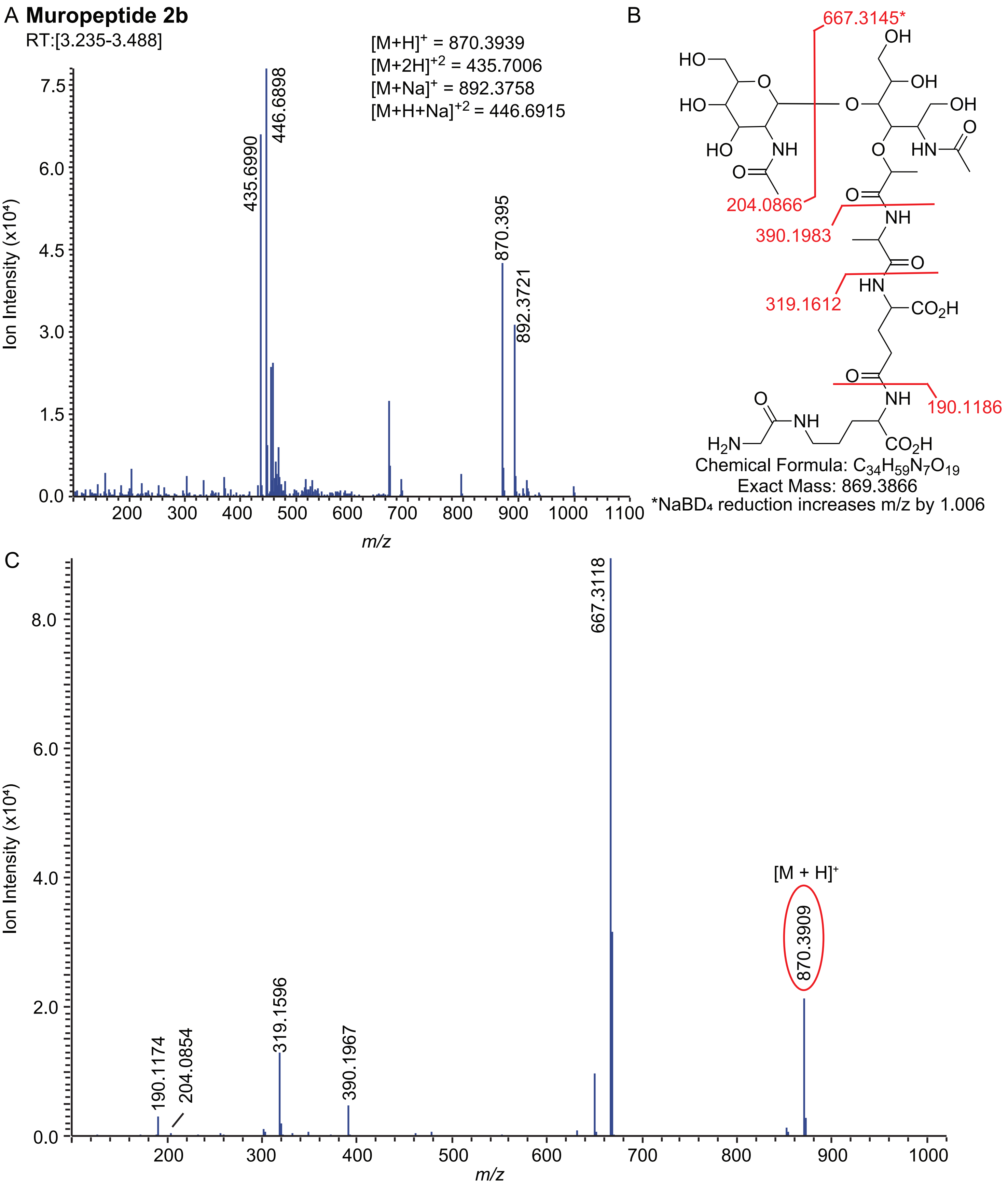

Supplemental Figure 4: (A) MS1 spectra generated from scanning retention times of 3.235-3.488 minutes. (B) The structure of muropeptide 2b. Cleavages with resulting *m/z* fragments are shown in red. (C) MS2 obtained from targeting precursor ion 870.3902 $[M+H]^+$. Red fragments generated in B correspond to observed MS2 fragments in C.

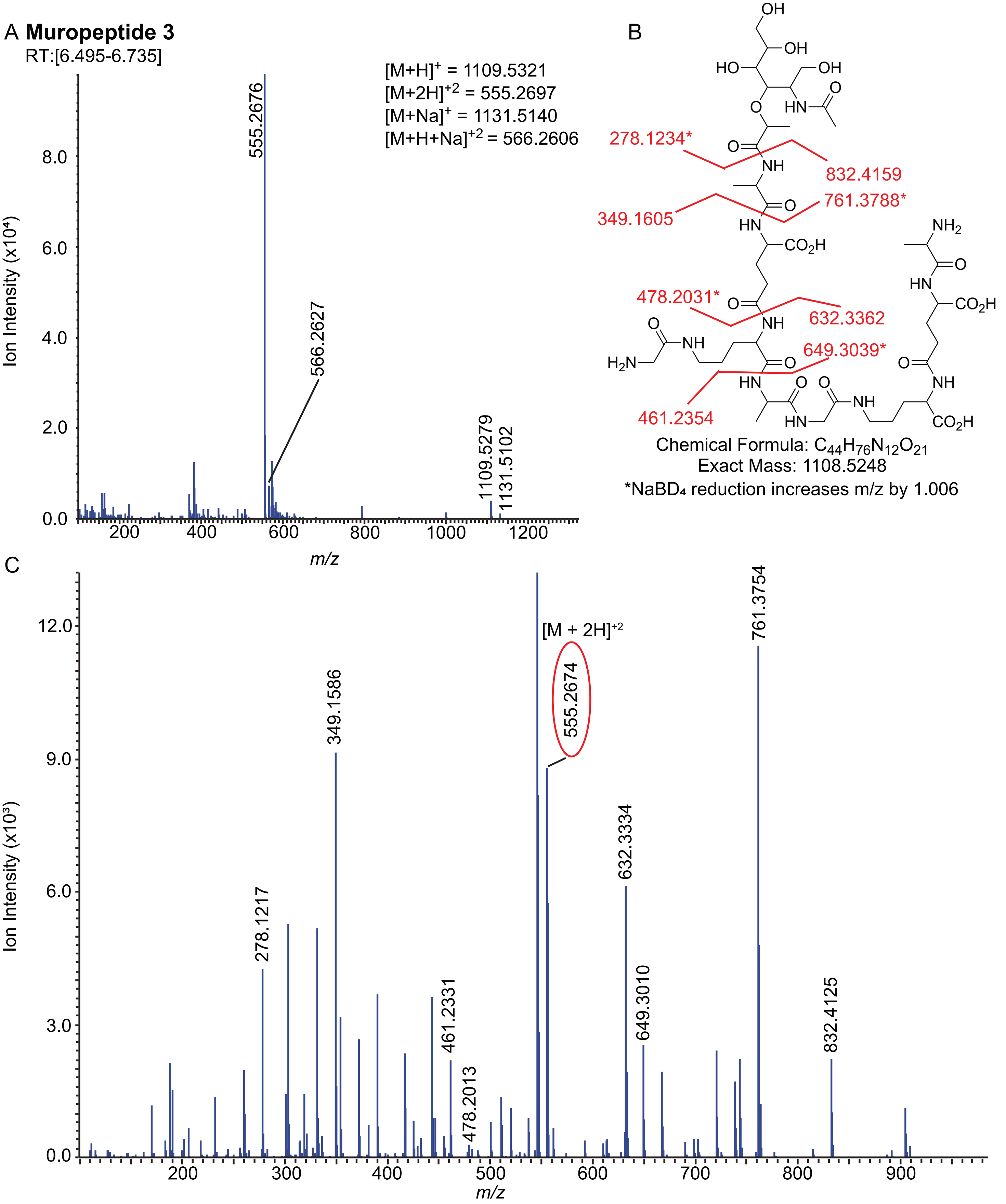

Supplemental Figure 5: (A) MS1 spectra generated from scanning retention times of 6.495-6.735 minutes. (B) The structure of muropeptide 3. Cleavages with resulting *m/z* fragments are shown in red. (C) MS2 obtained from targeting precursor ion 555.2676 $[M+2H]^{+2}$. Red fragments generated in B correspond to observed MS2 fragments in C.

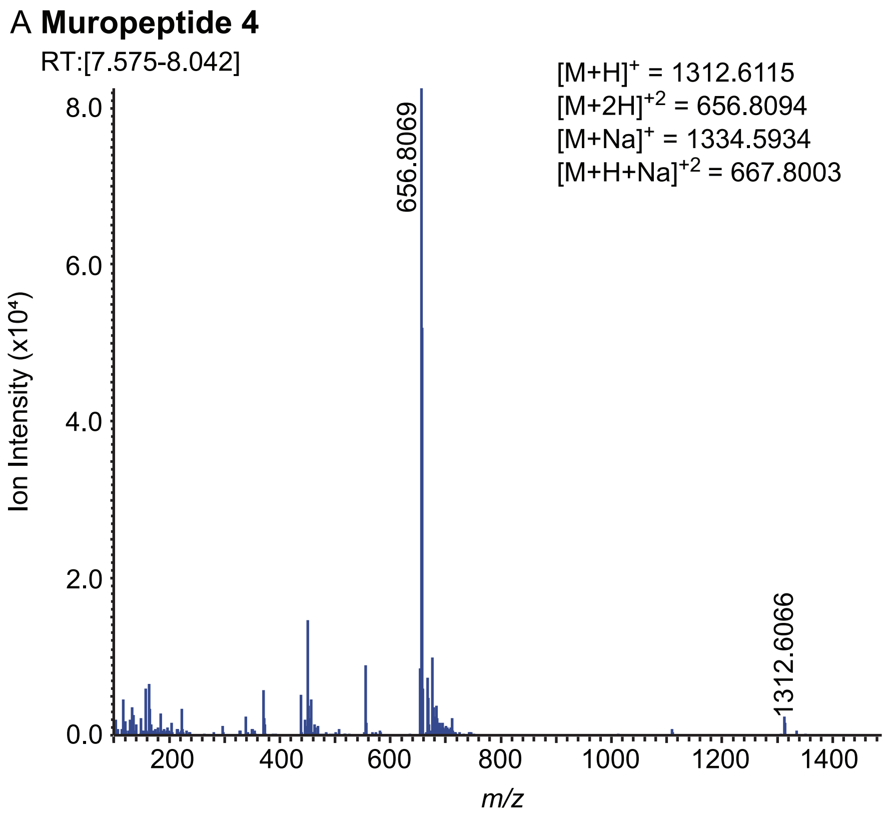

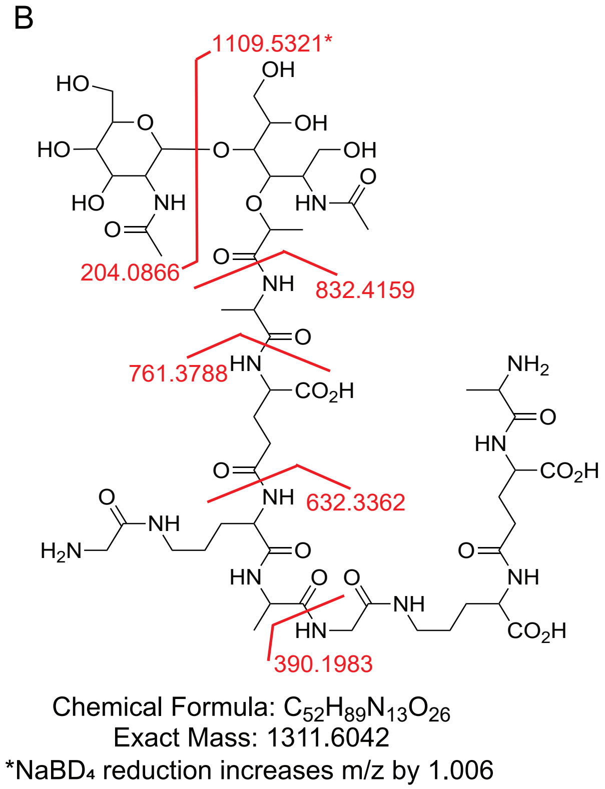

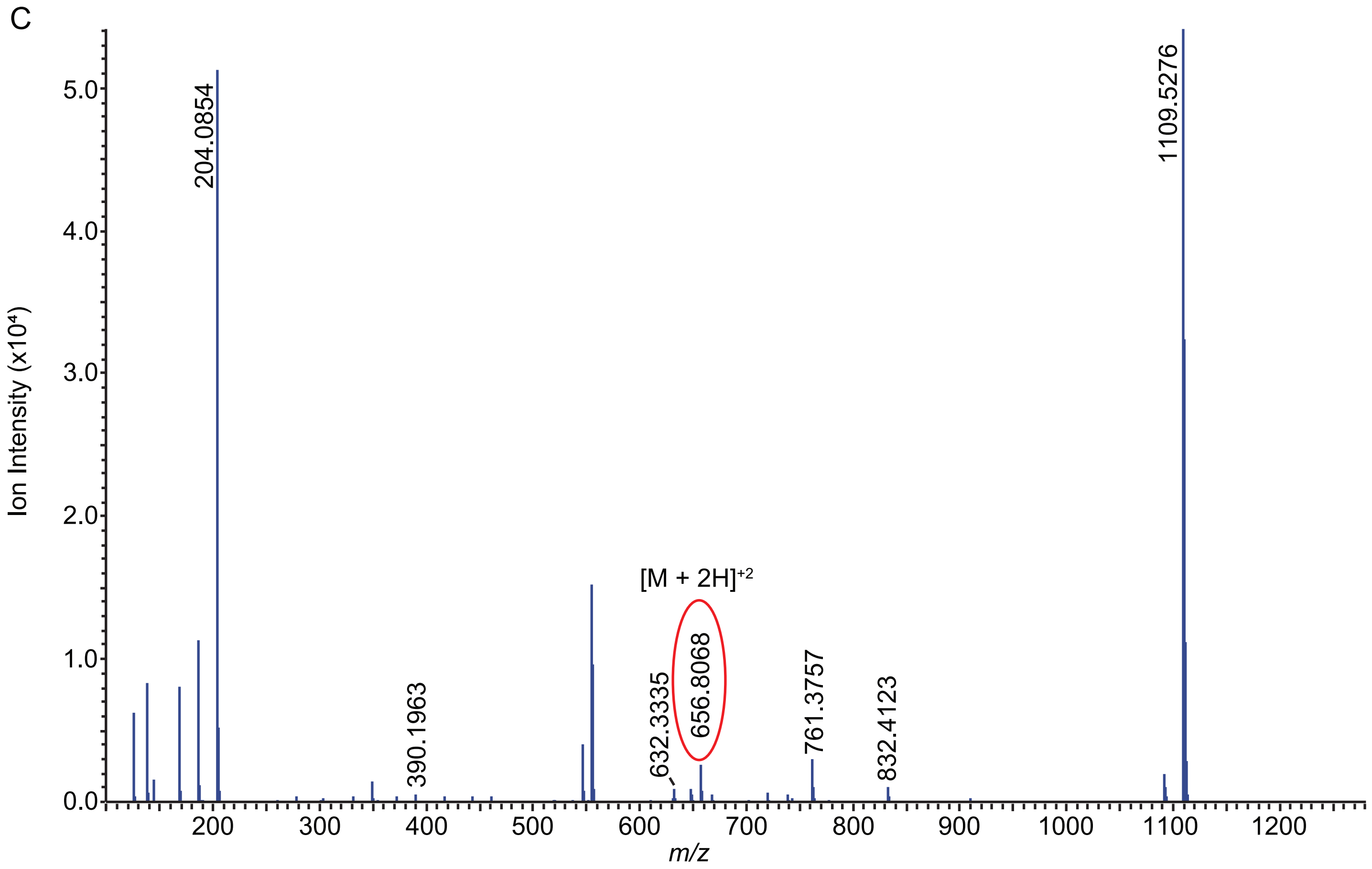

Supplemental Figure 6: (A) MS1 spectra generated from scanning retention times of 7.575-8.042 minutes. (B) The structure of muropeptide 4. Cleavages with resulting *m/z* fragments are shown in red. (C) MS2 obtained from targeting precursor ion 656.8094 $[M+2H]^{+2}$. Red fragments generated in B correspond to observed MS2 fragments in C.

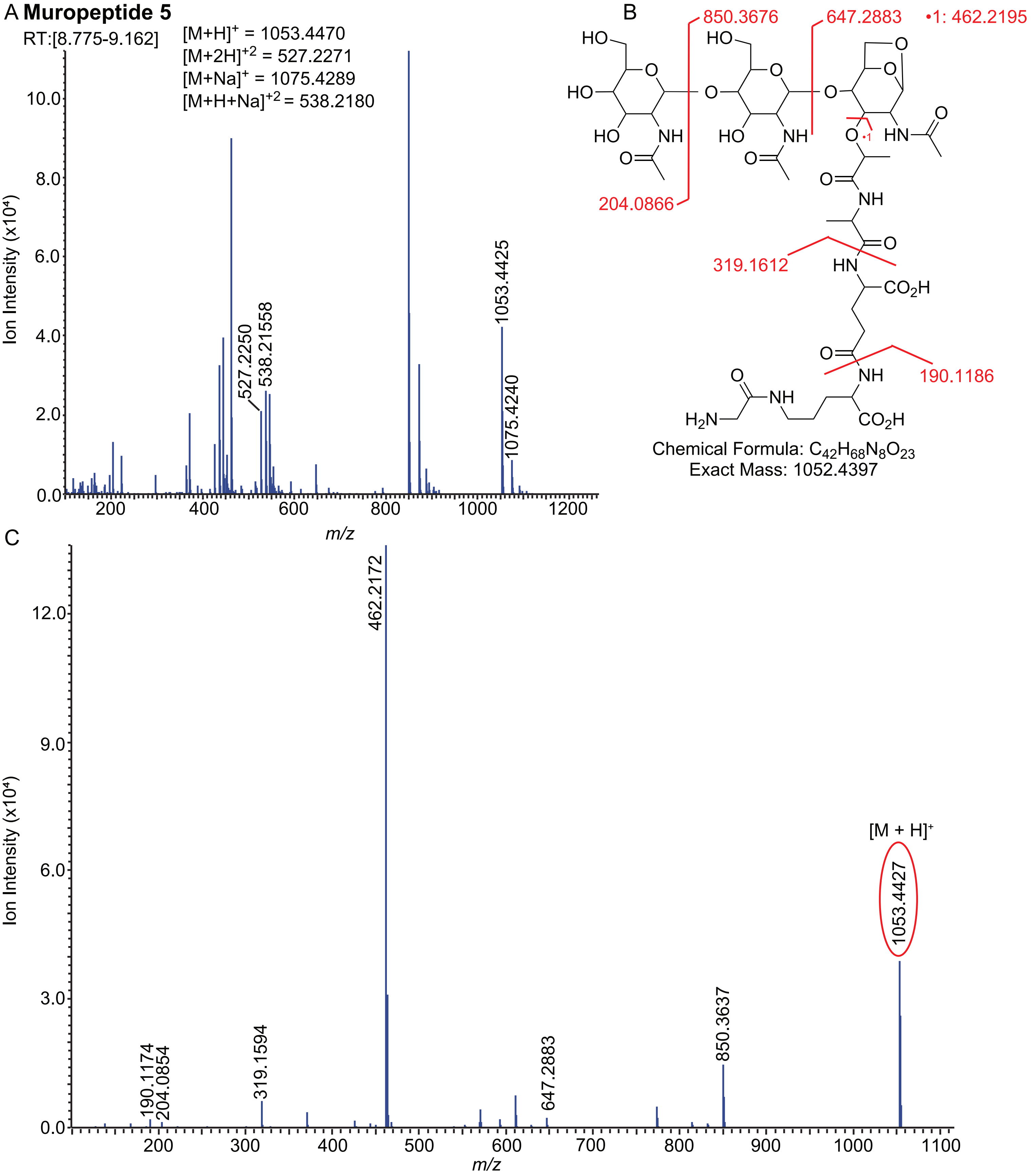

Supplemental Figure 7: (A) MS1 spectra generated from scanning retention times of 8.775-9.162 minutes. (B) The structure of muropeptide 5. Cleavages with resulting *m/z* fragments are shown in red. (C) MS2 obtained from targeting precursor ion 1053.4426 $[M+H]^+$. Red fragments generated in B correspond to observed MS2 fragments in C.

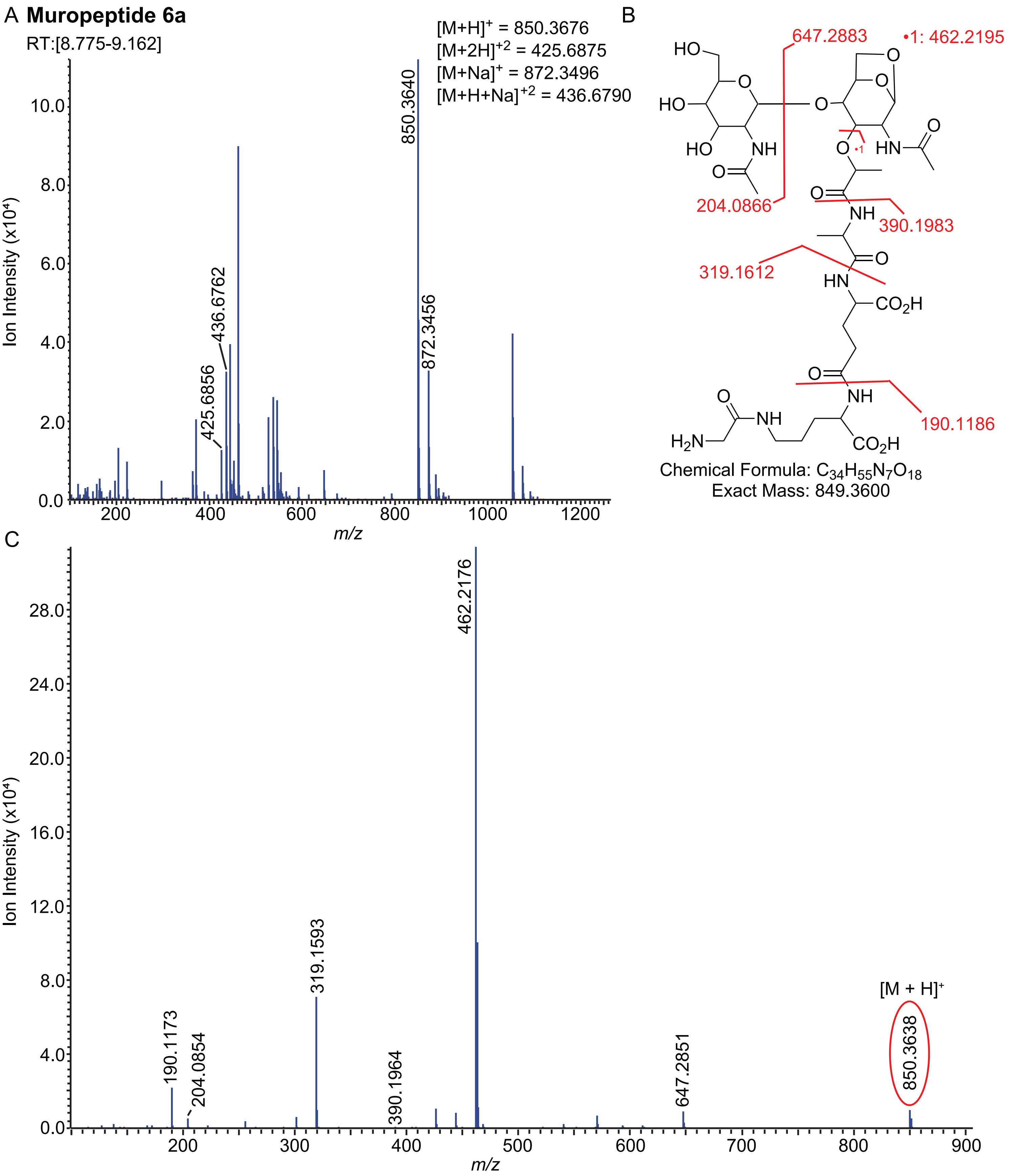

Supplemental Figure 8: (A) MS1 spectra generated from scanning retention times of 8.775-9.162 minutes. (B) The structure of muropeptide 6a. Cleavages with resulting *m/z* fragments are shown in red. (C) MS2 obtained from targeting precursor ion 850.3676 $[M+H]^+$. Red fragments generated in B correspond to observed MS2 fragments in C.

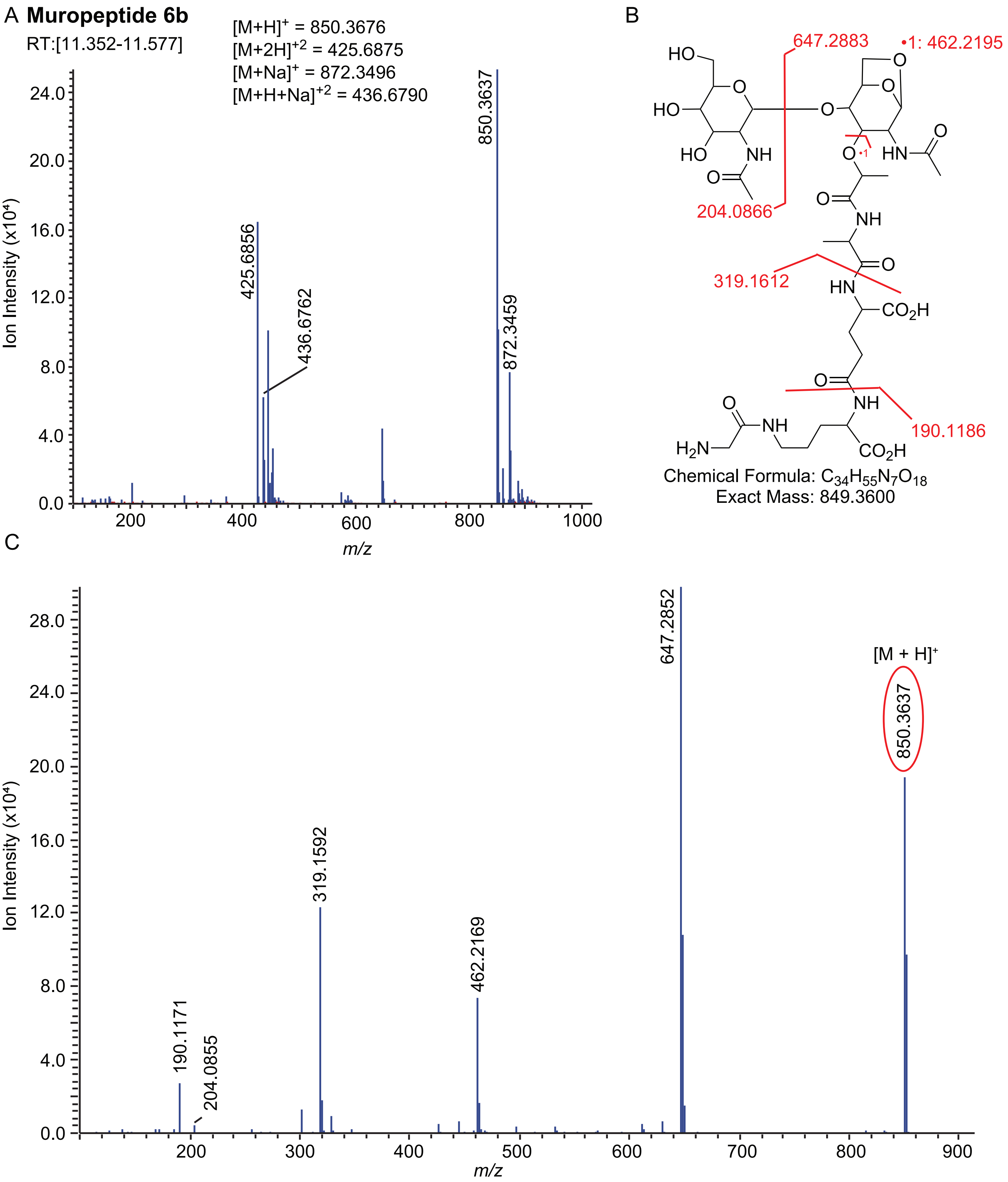

Supplemental Figure 9: (A) MS1 spectra generated from scanning retention times of 11.352-11.577 minutes. (B) The structure of muropeptide 6b. Cleavages with resulting *m/z* fragments are shown in red. (C) MS2 obtained from targeting precursor ion 850.3676 $[M+H]^+$. Red fragments generated in B correspond to observed MS2 fragments in C.

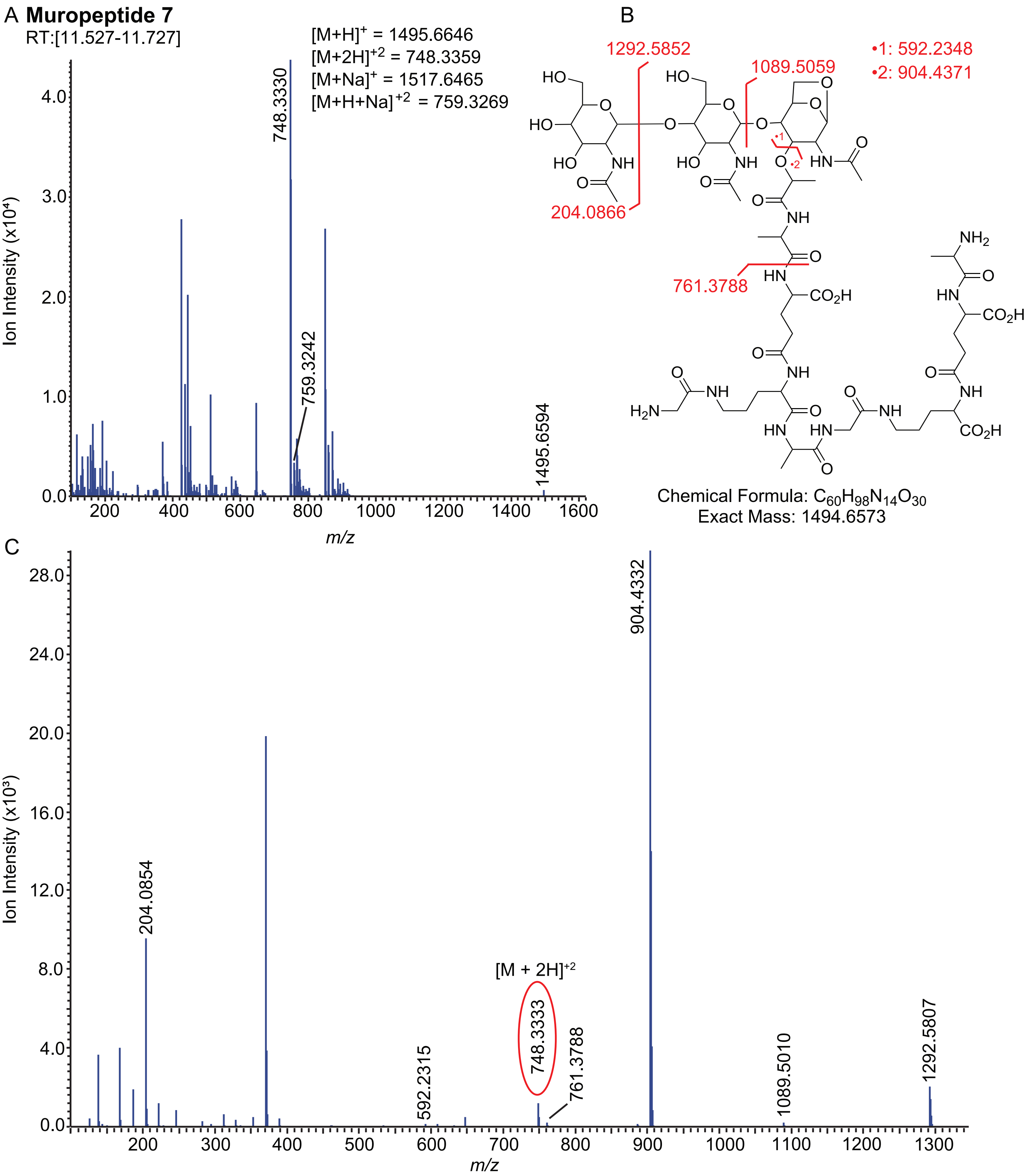

Supplemental Figure 10: (A) MS1 spectra generated from scanning retention times of 11.527-11.727 minutes. (B) The structure of muropeptide 7. Cleavages with resulting *m/z* fragments are shown in red. (C) MS2 obtained from targeting precursor ion 748.3359 $[M+2H]^{+2}$. Red fragments generated in B correspond to observed MS2 fragments in C.

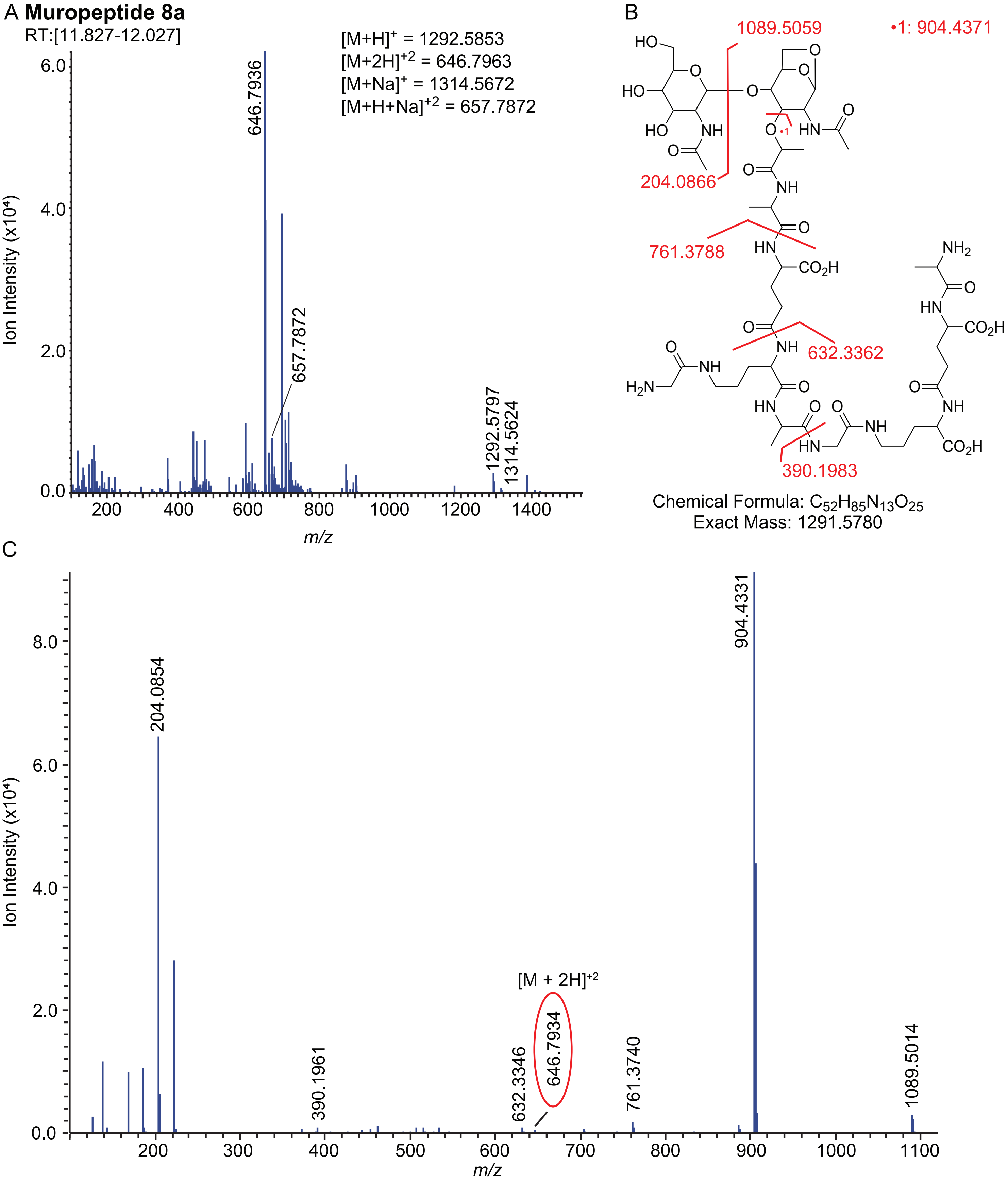

Supplemental Figure 11: (A) MS1 spectra generated from scanning retention times of 11.827-12.027 minutes. (B) The structure of muropeptide 8a. Cleavages with resulting *m/z* fragments are shown in red. (C) MS2 obtained from targeting precursor ion 646.7963 $[M+2H]^{+2}$. Red fragments generated in B correspond to observed MS2 fragments in C.

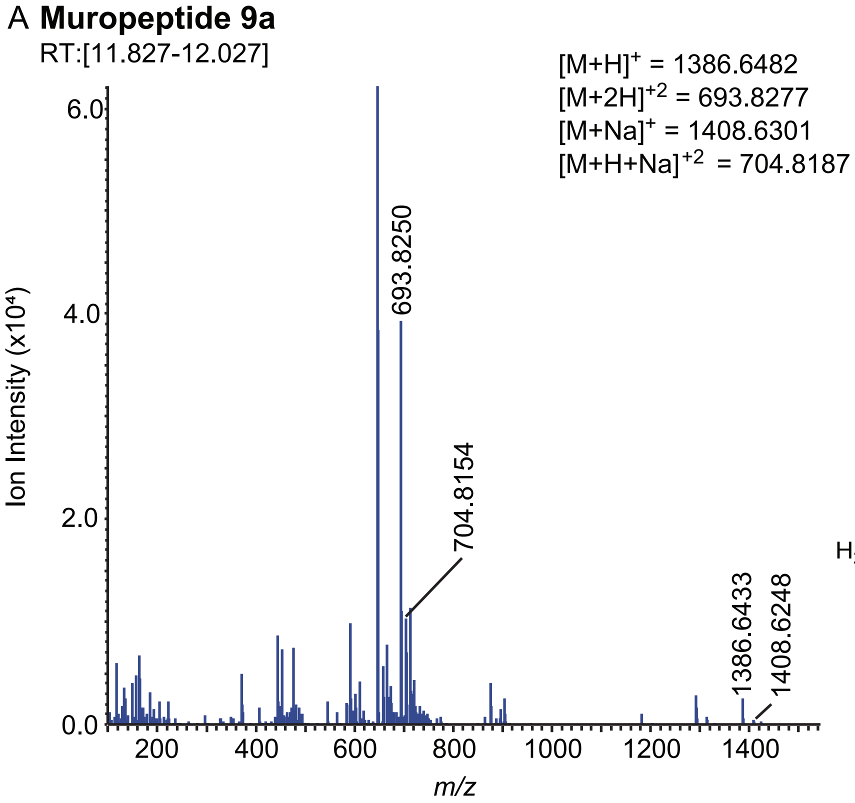

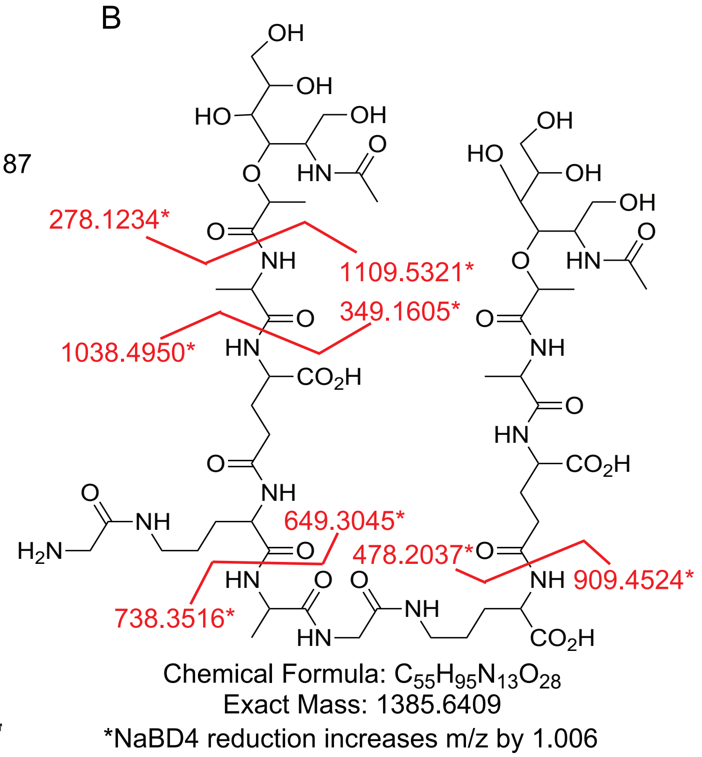

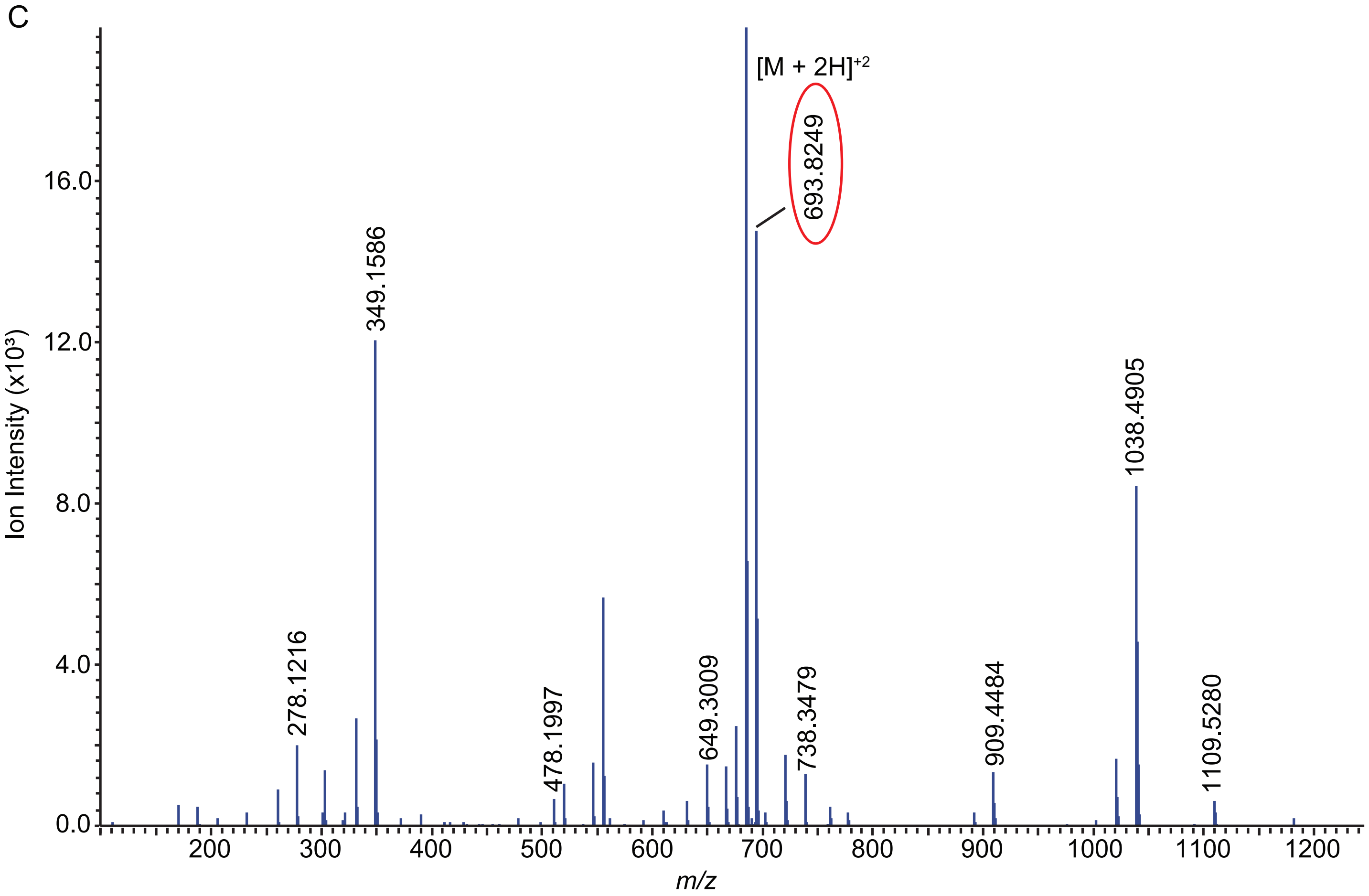

Supplemental Figure 12: (A) MS1 spectra generated from scanning retention times of 11.827-12.027 minutes. (B) The structure of muropeptide 9a. Cleavages with resulting *m/z* fragments are shown in red. (C) MS2 obtained from targeting precursor ion 693.8277 $[M+2H]^{+2}$. Red fragments generated in B correspond to observed MS2 fragments in C.

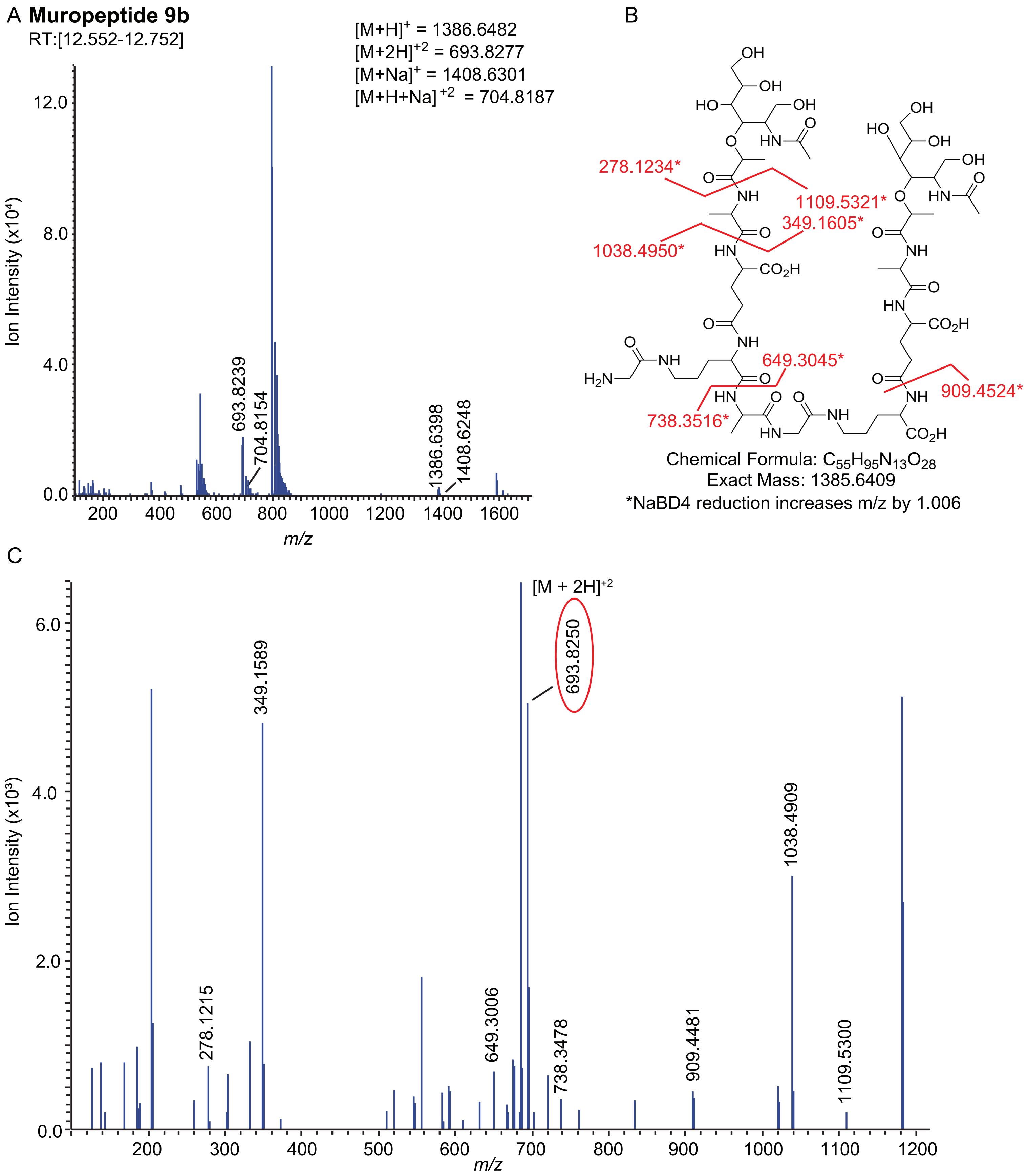

Supplemental Figure 13: (A) MS1 spectra generated from scanning retention times of 12.552-12.752 minutes. (B) The structure of muropeptide 9b. Cleavages with resulting *m/z* fragments are shown in red. (C) MS2 obtained from targeting precursor ion 693.8277 $[M+2H]^{+2}$. Red fragments generated in B correspond to observed MS2 fragments in C.

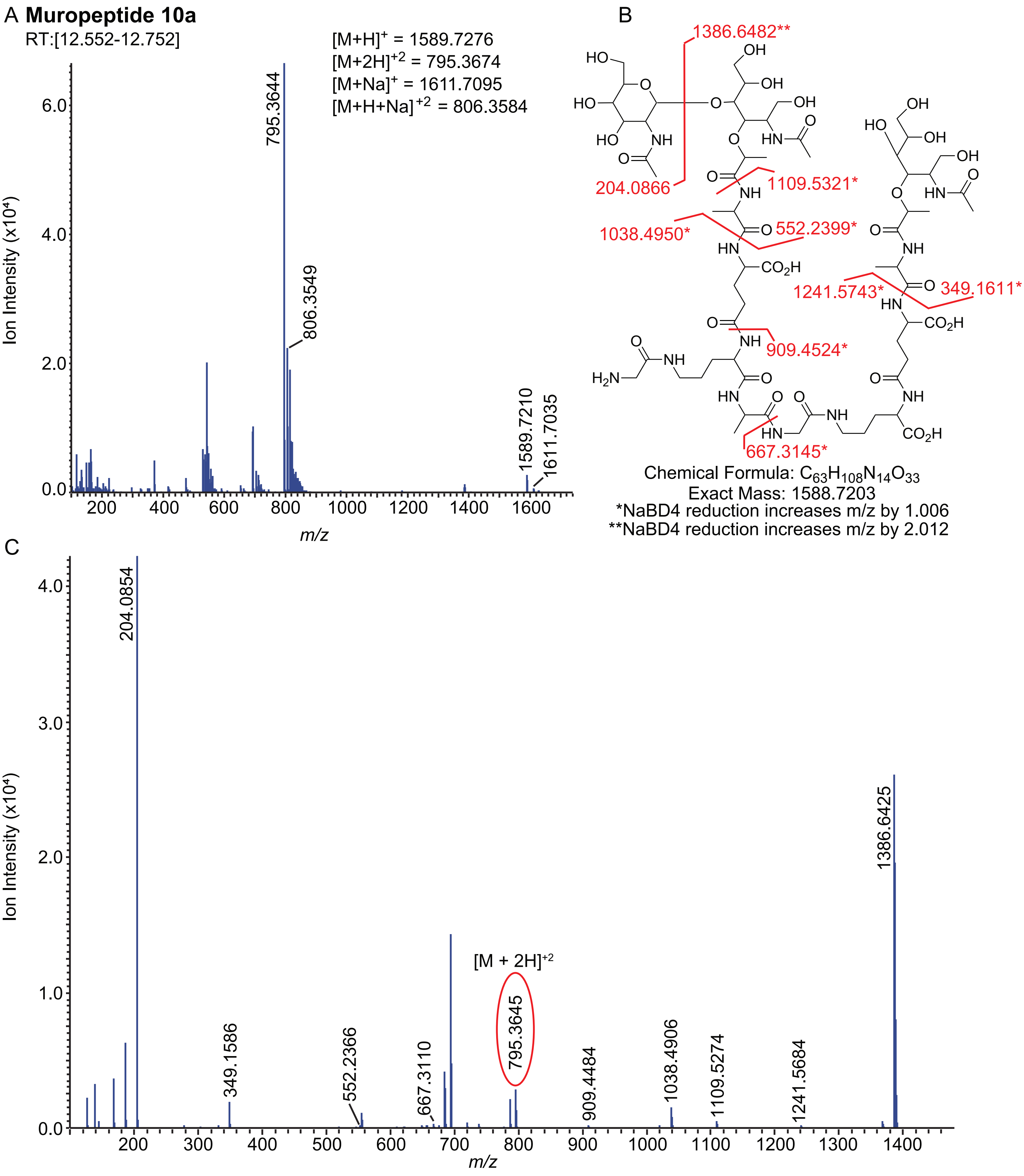

Supplemental Figure 14: (A) MS1 spectra generated from scanning retention times of 12.552-12.752 minutes. (B) The structure of muropeptide 10a. Cleavages with resulting *m/z* fragments are shown in red. (C) MS2 obtained from targeting precursor ion 795.3674 $[M+2H]^{+2}$. Red fragments generated in B correspond to observed MS2 fragments in C.

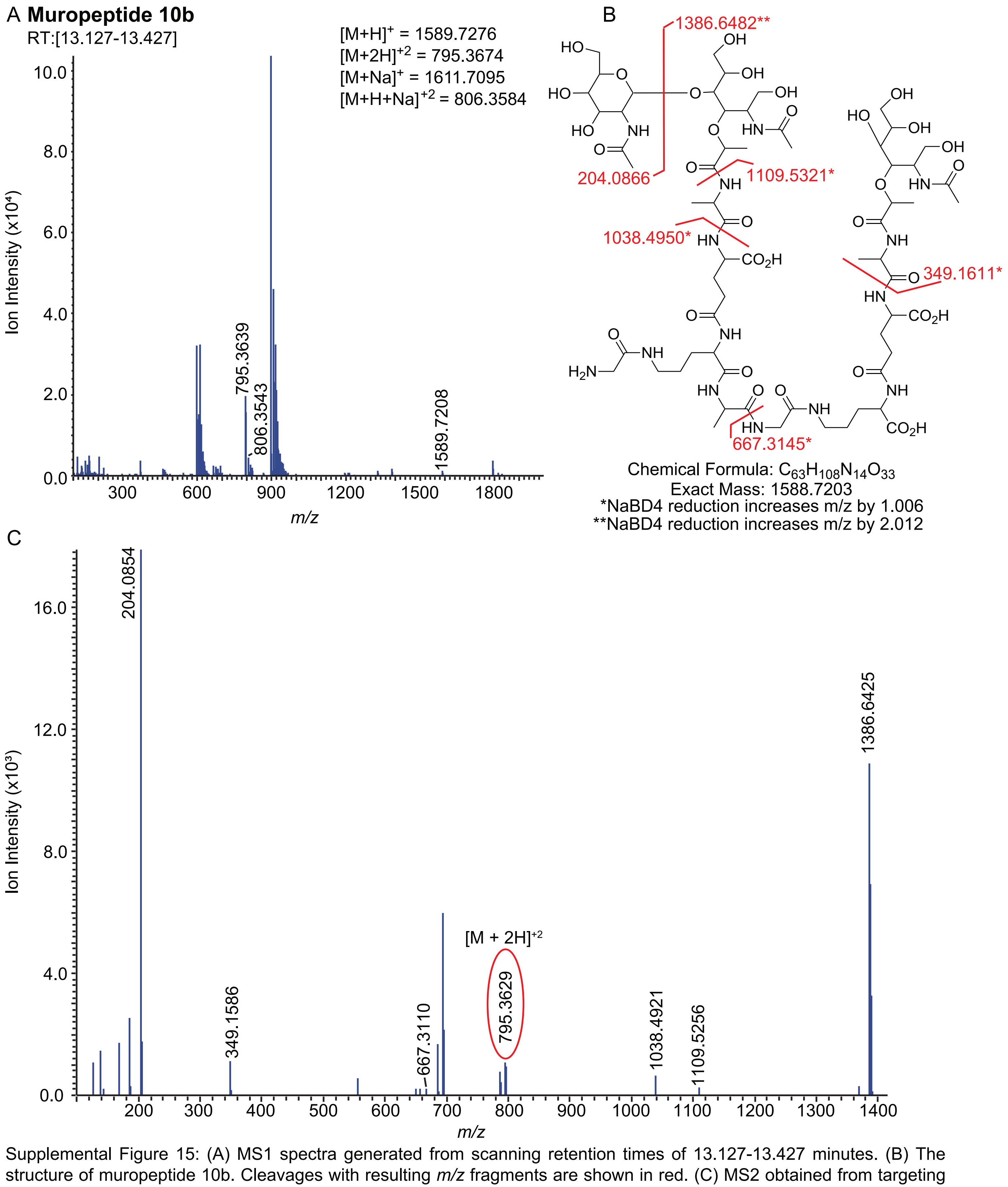

Supplemental Figure 15: (A) MS1 spectra generated from scanning retention times of 13.127-13.427 minutes. (B) The structure of muropeptide 10b. Cleavages with resulting *m/z* fragments are shown in red. (C) MS2 obtained from targeting precursor ion 795.3674 $[M+2H]^{+2}$. Red fragments generated in B correspond to observed MS2 fragments in C.

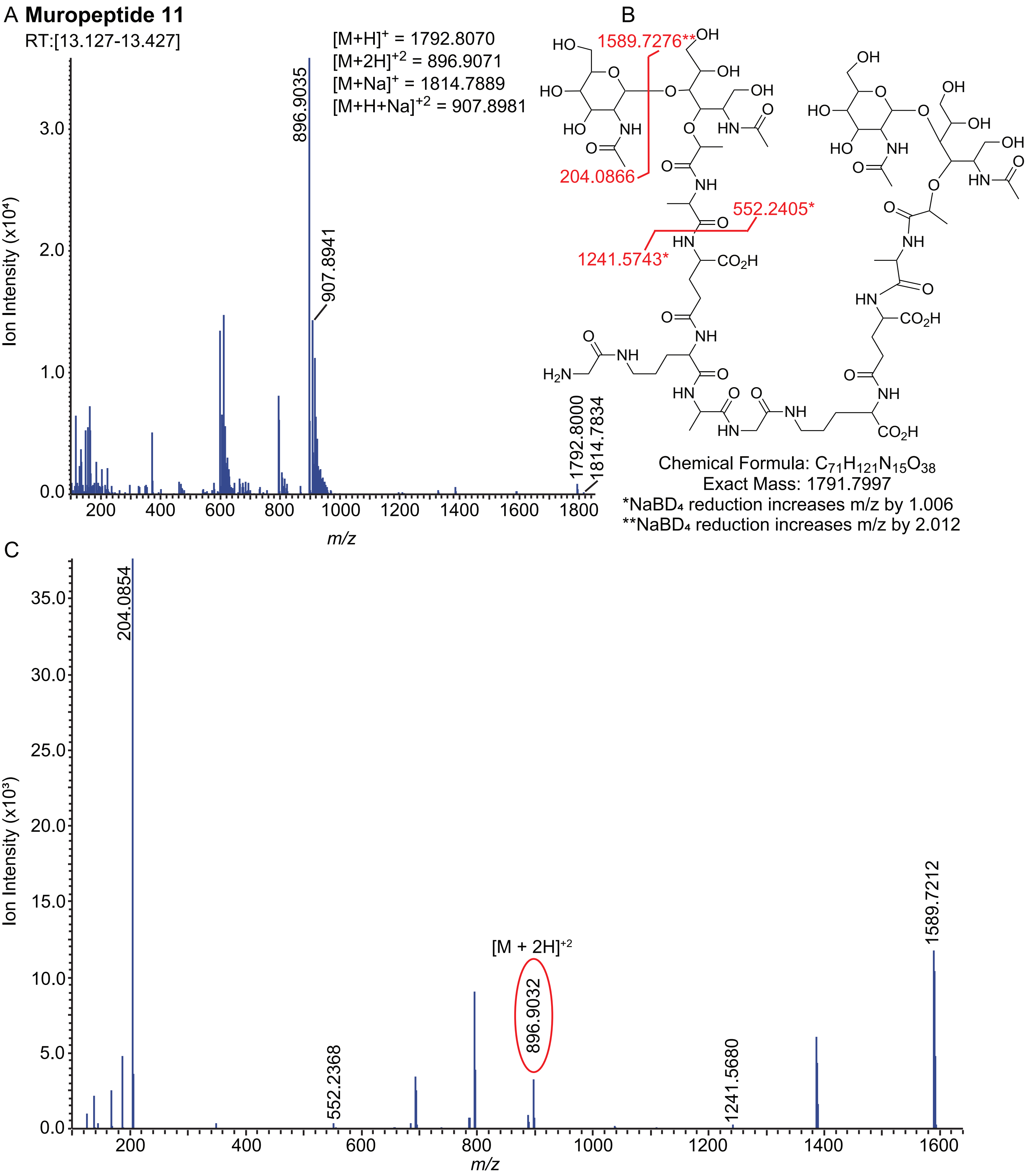

Supplemental Figure 16: (A) MS1 spectra generated from scanning retention times of 13.127-13.427 minutes. (B) The structure of muropeptide 11. Cleavages with resulting *m/z* fragments are shown in red. (C) MS2 obtained from targeting precursor ion 896.9035 $[M+H]^{+2}$. Red fragments generated in B correspond to observed MS2 fragments in C.

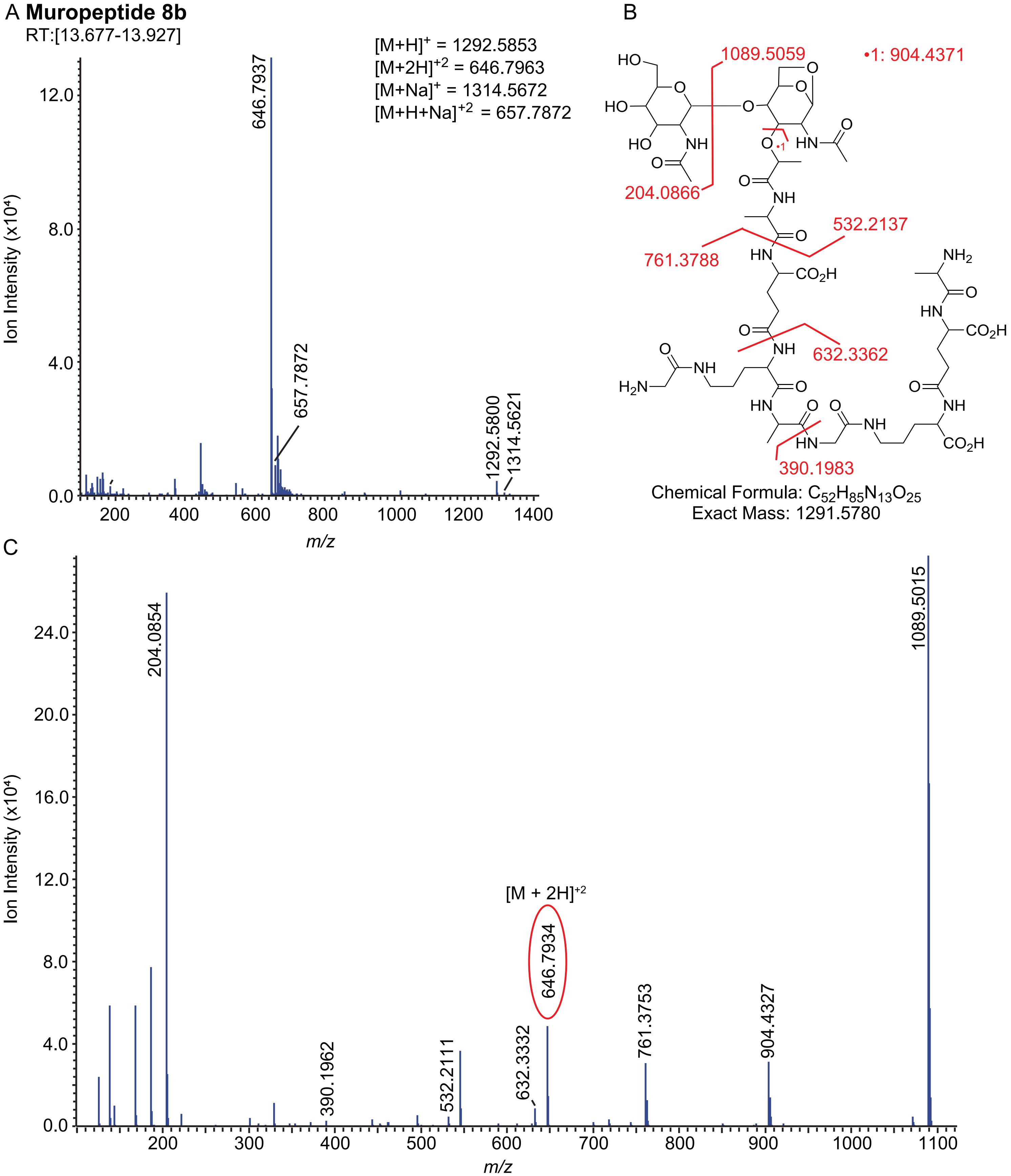

Supplemental Figure 17: (A) MS1 spectra generated from scanning retention times of 13.667-13.927 minutes. (B) The structure of muropeptide 8b. Cleavages with resulting *m/z* fragments are shown in red. (C) MS2 obtained from targeting precursor ion 646.7963 $[M+2H]^{+2}$. Red fragments generated in B correspond to observed MS2 fragments in C.

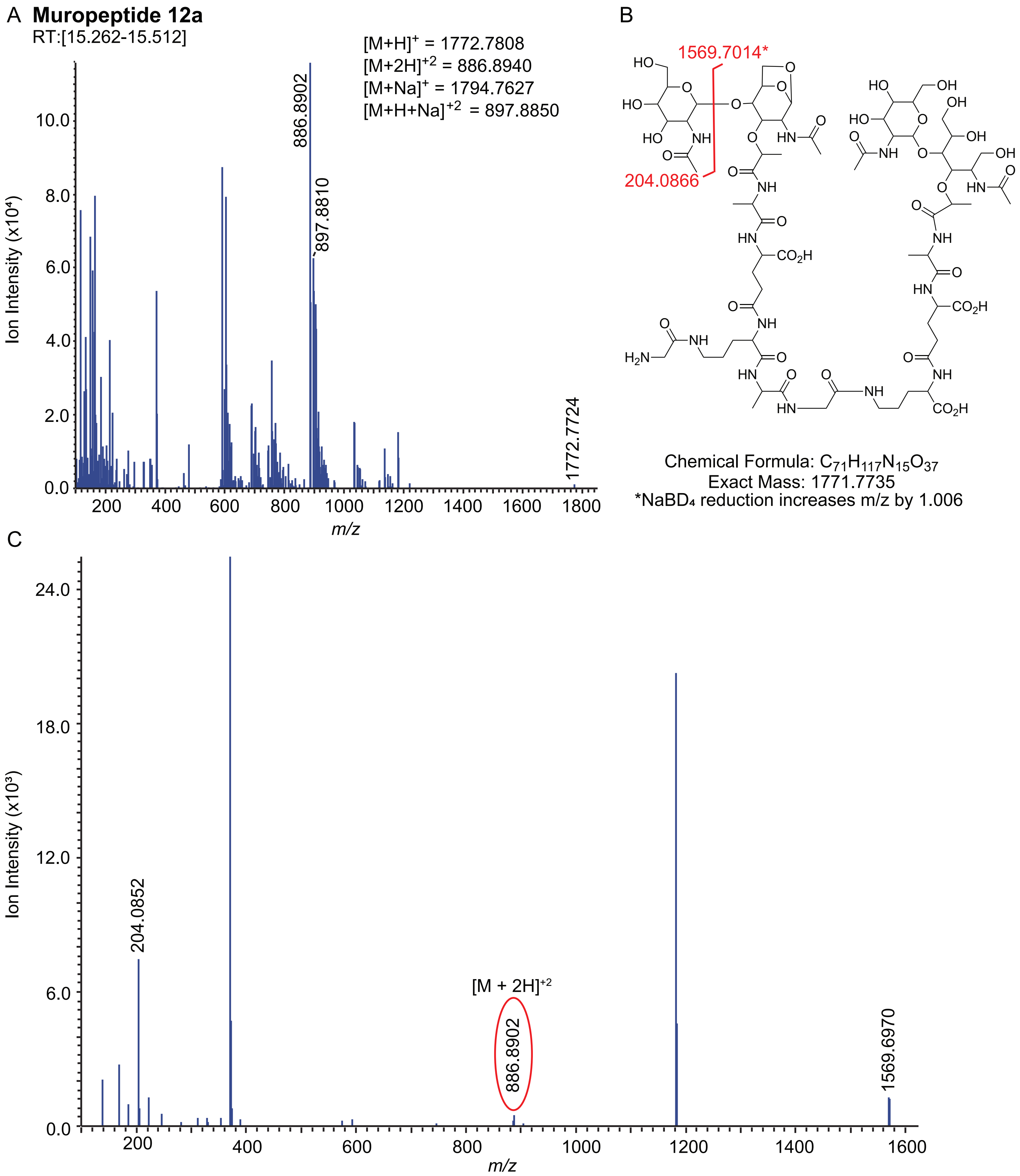

Supplemental Figure 18: (A) MS1 spectra generated from scanning retention times of 15.262-15.512 minutes. (B) The structure of muropeptide 12a. Cleavages with resulting *m/z* fragments are shown in red. (C) MS2 obtained from targeting precursor ion 886.8902 $[M+H]^{+2}$. Red fragments generated in B correspond to observed MS2 fragments in C.

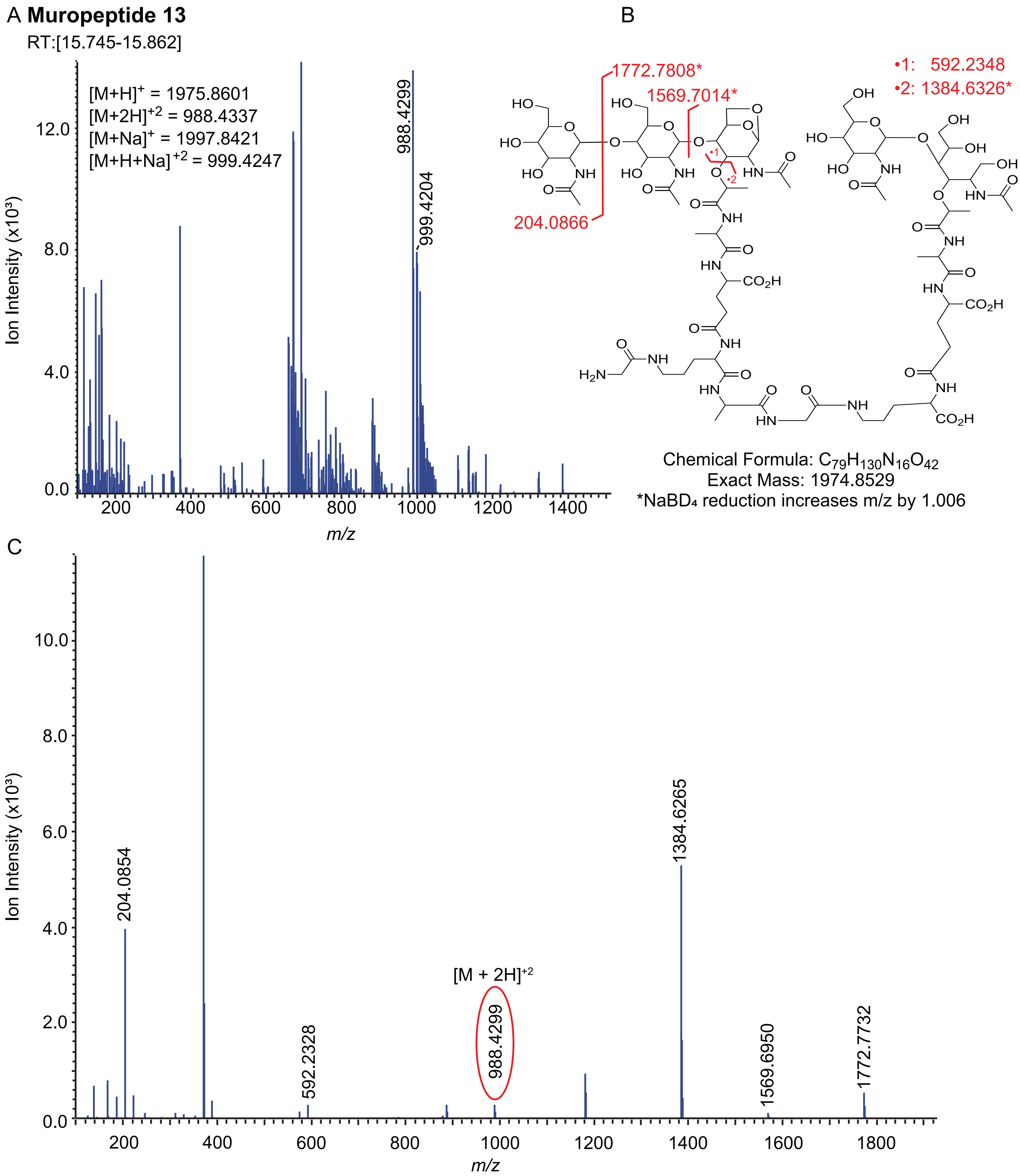

Supplemental Figure 19: (A) MS1 spectra generated from scanning retention times of 15.745-15.862 minutes. (B) The structure of muropeptide 13. Cleavages with resulting *m/z* fragments are shown in red. (C) MS2 obtained from targeting precursor ion 988.4299 $[M+H]^{+2}$. Red fragments generated in B correspond to observed MS2 fragments in C.

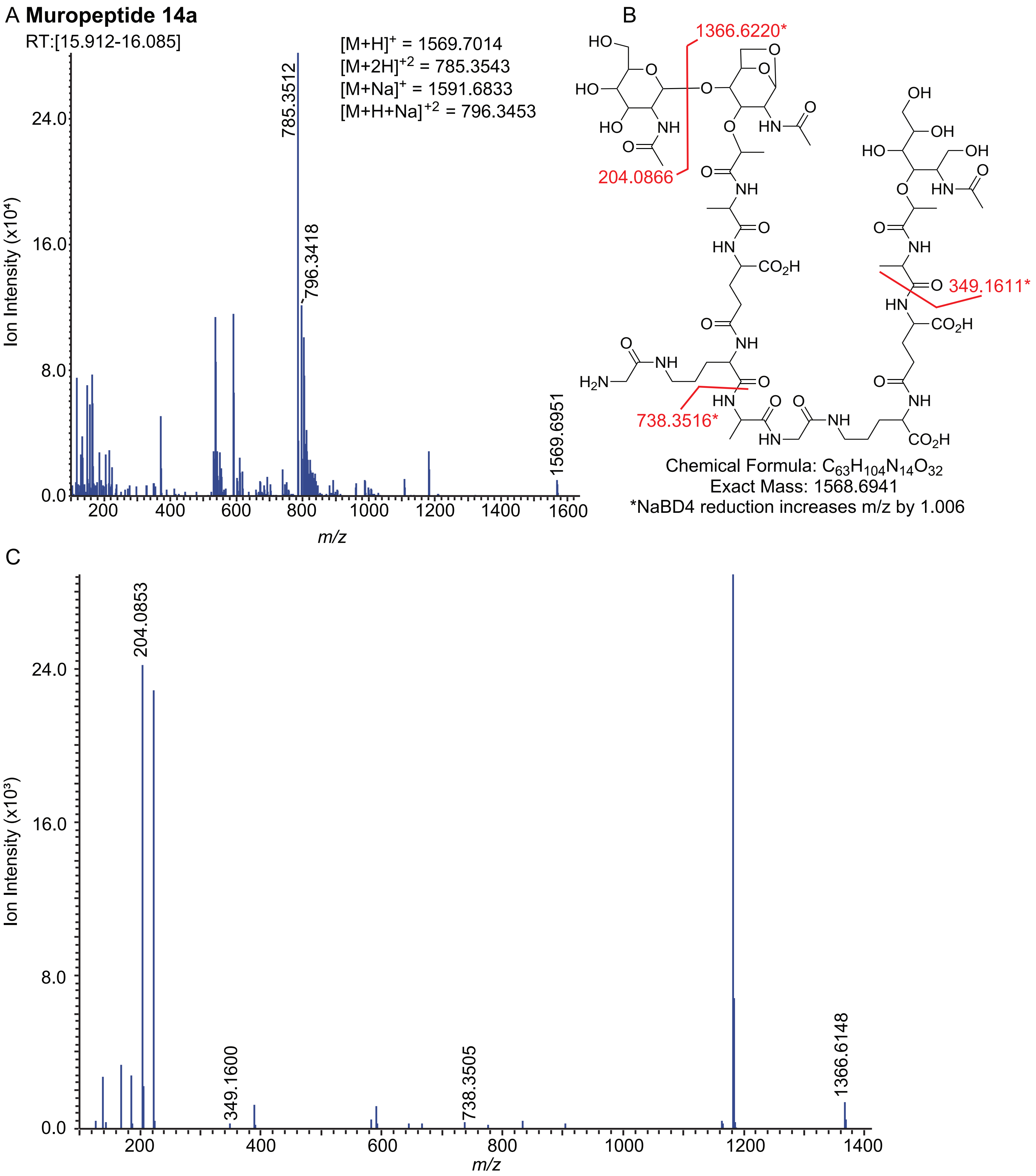

Supplemental Figure 20: (A) MS1 spectra generated from scanning retention times of 15.912-16.085 minutes. (B) The structure of muropeptide 14a. Cleavages with resulting *m/z* fragments are shown in red. (C) MS2 obtained from targeting precursor ion 785.3512 $[M+H]^{+2}$. Red fragments generated in B correspond to observed MS2 fragments in C.

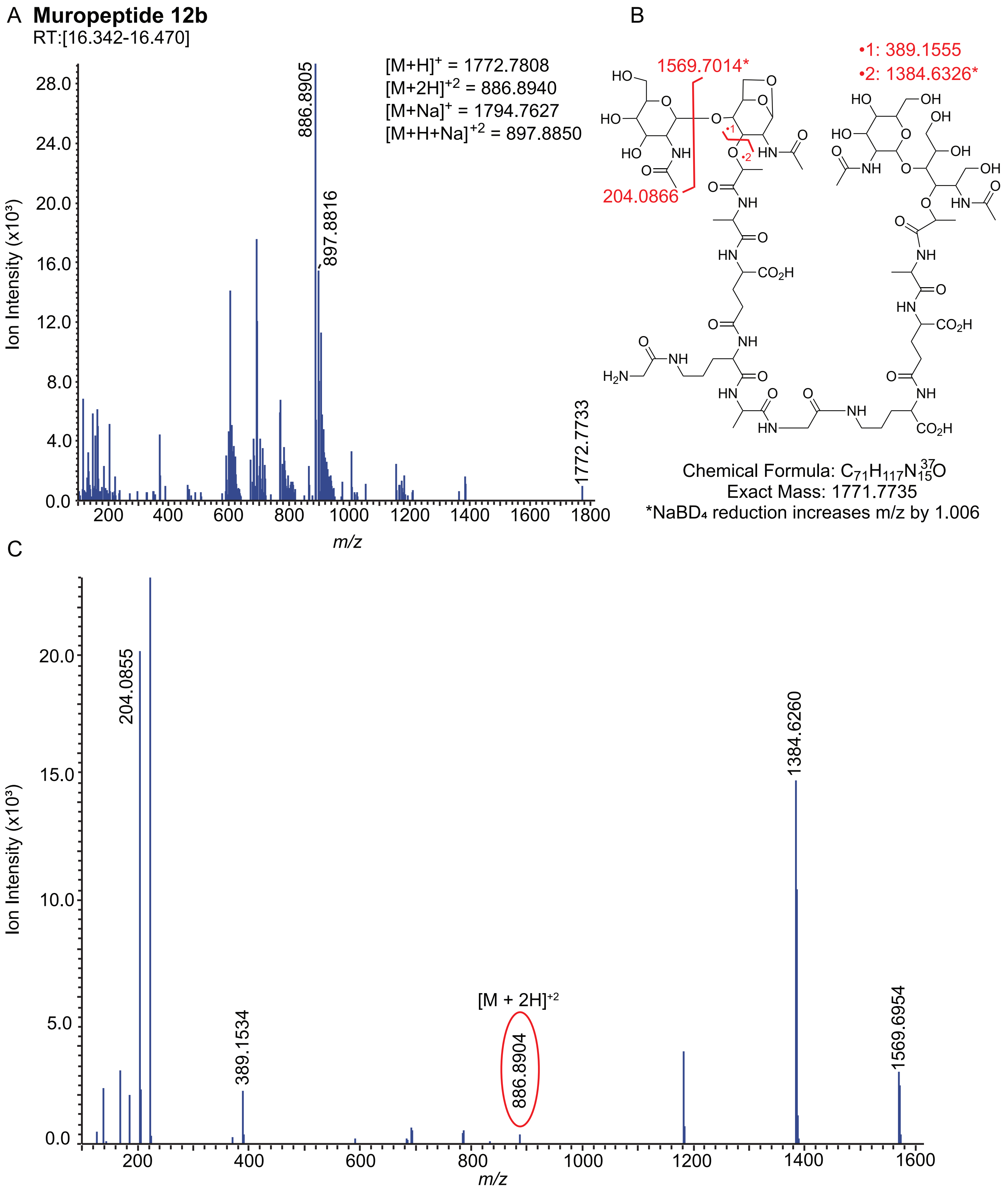

Supplemental Figure 21: (A) MS1 spectra generated from scanning retention times of 16.342-16.470 minutes. (B) The structure of muropeptide 12b. Cleavages with resulting *m/z* fragments are shown in red. (C) MS2 obtained from targeting precursor ion 886.8902 $[M+H]^{+2}$. Red fragments generated in B correspond to observed MS2 fragments in C.

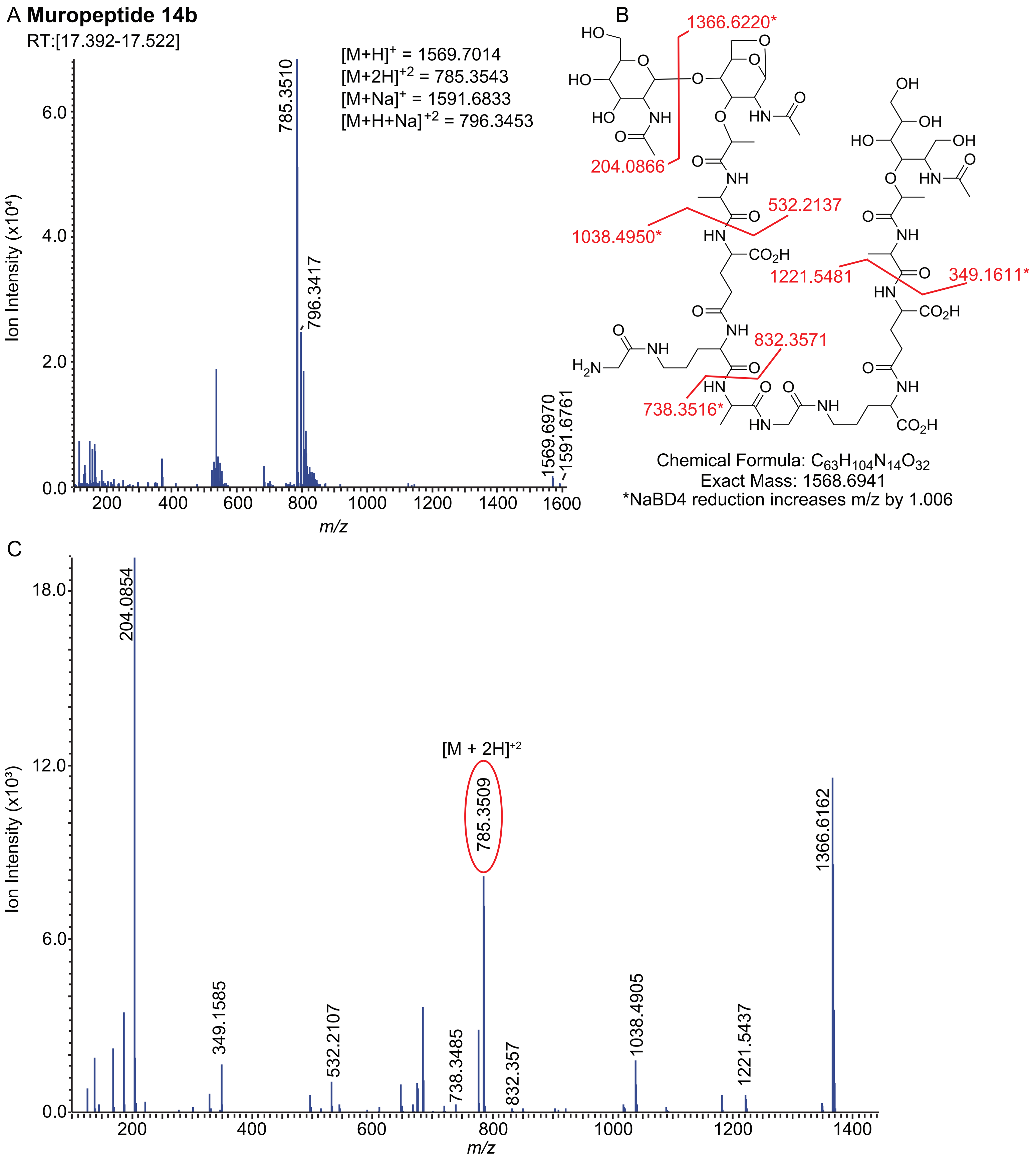

Supplemental Figure 22: (A) MS1 spectra generated from scanning retention times of 17.392-17.522 minutes. (B) The structure of muropeptide 14b. Cleavages with resulting *m/z* fragments are shown in red. (C) MS2 obtained from targeting precursor ion 785.3512 $[M+H]^{+2}$. Red fragments generated in B correspond to observed MS2 fragments in C.

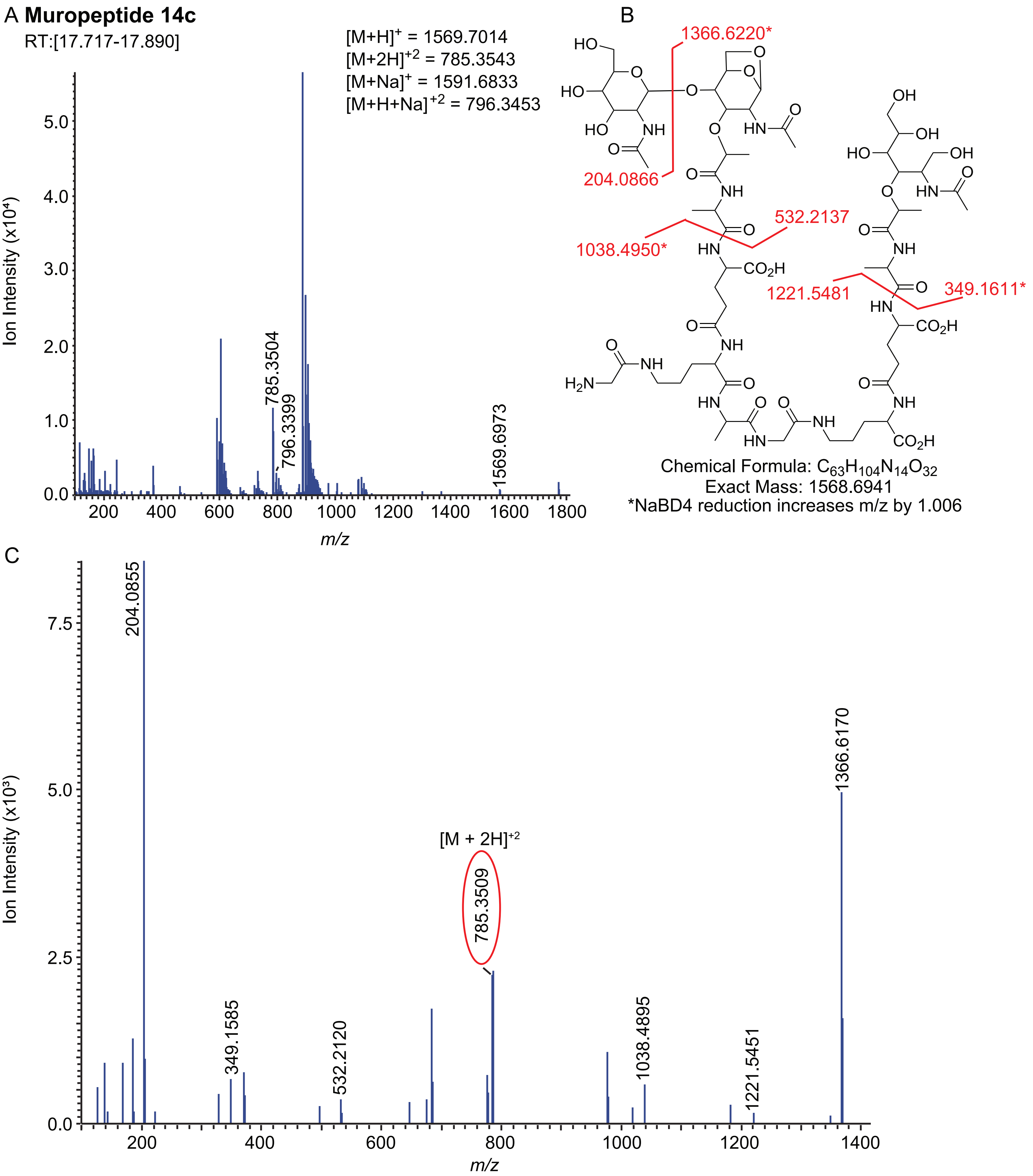

Supplemental Figure 23: (A) MS1 spectra generated from scanning retention times of 17.717-17.890 minutes. (B) The structure of muropeptide 14c. Cleavages with resulting *m/z* fragments are shown in red. (C) MS2 obtained from targeting precursor ion 785.3512 $[M+H]^{+2}$. Red fragments generated in B correspond to observed MS2 fragments in C.

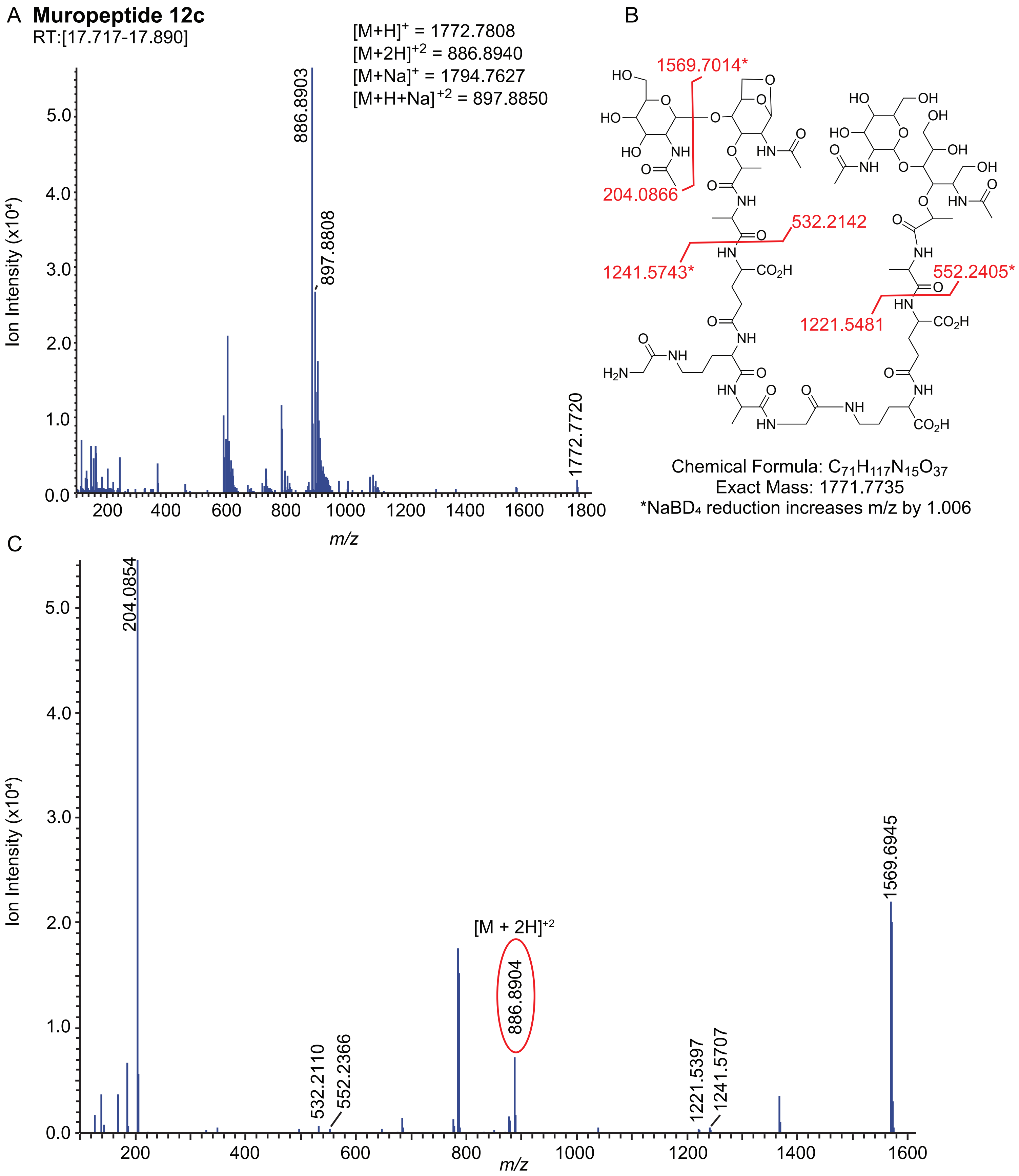

Supplemental Figure 24: (A) MS1 spectra generated from scanning retention times of 17.717-17.890 minutes. (B) The structure of muropeptide 12c. Cleavages with resulting *m/z* fragments are shown in red. (C) MS2 obtained from targeting precursor ion 886.8902 $[M+H]^{+2}$. Red fragments generated in B correspond to observed MS2 fragments in C.

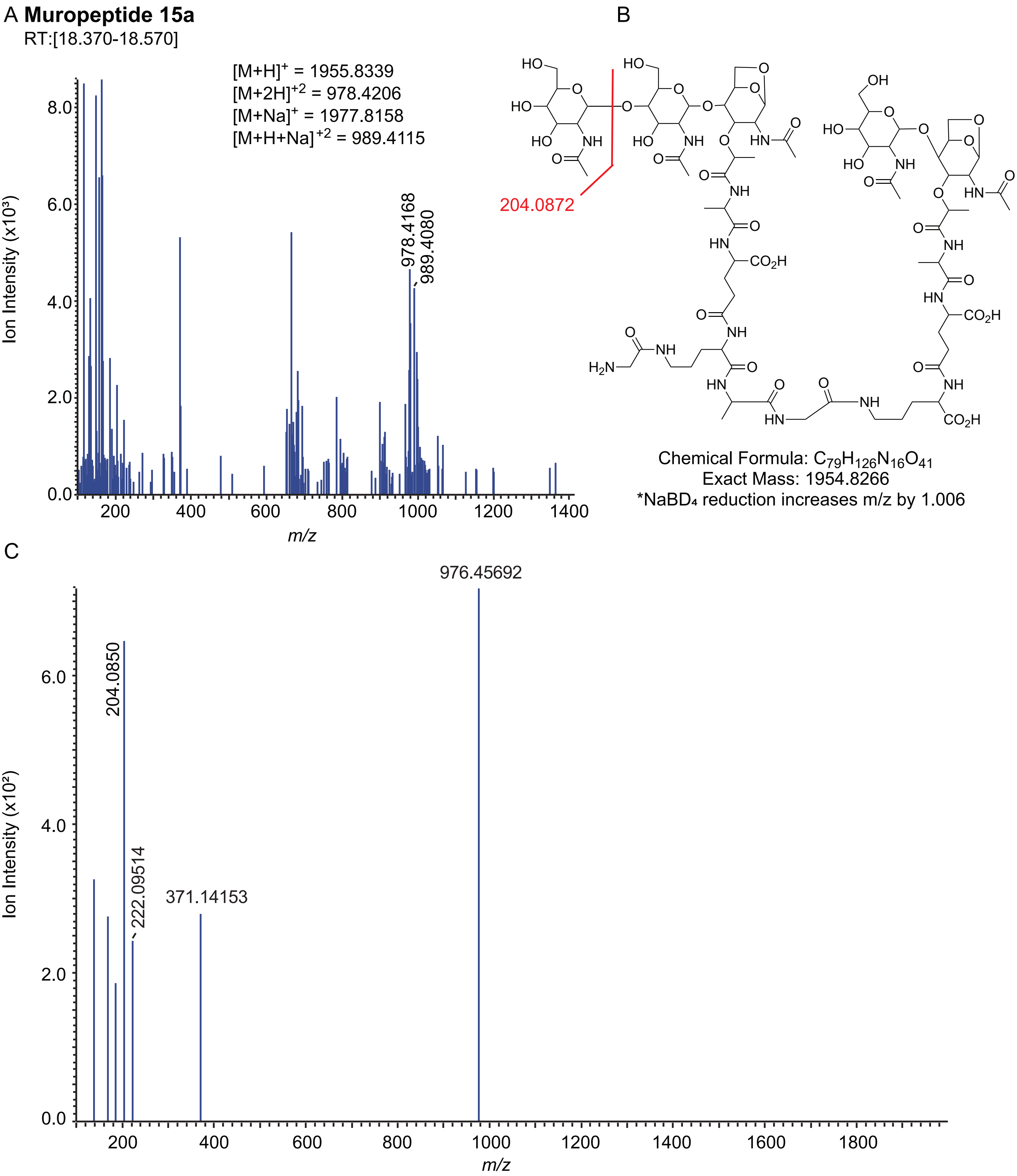

Supplemental Figure 25: (A) MS1 spectra generated from scanning retention times of 18.370-18.570 minutes. (B) The structure of muropeptide 15a. Cleavages with resulting *m/z* fragments are shown in red. (C) MS2 obtained from targeting precursor ion 978.417 $[M+H]^{+2}$. Red fragments generated in B correspond to observed MS2 fragments in C.

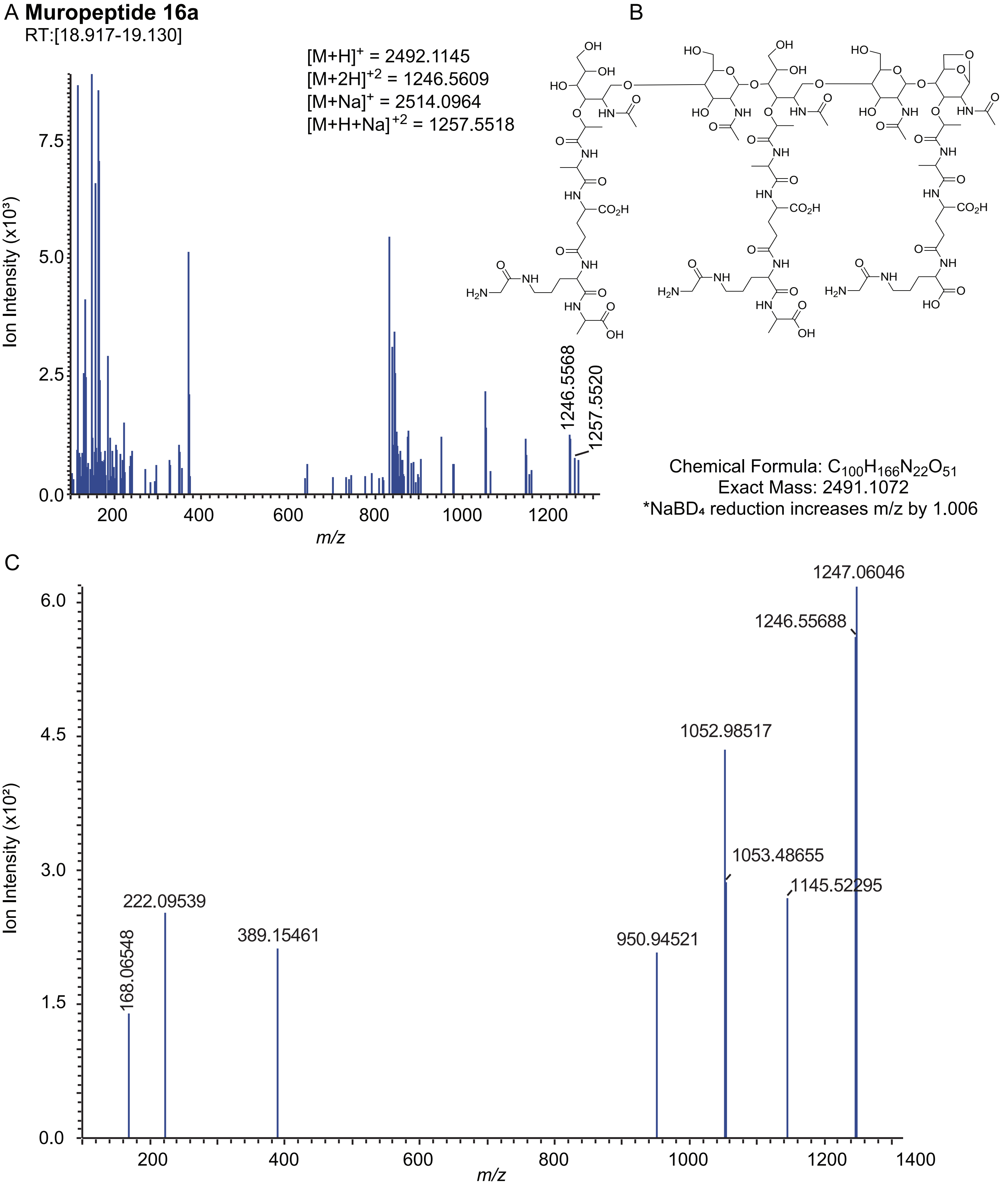

Supplemental Figure 26: (A) MS1 spectra generated from scanning retention times of 18.917-19.130 minutes. (B) The structure of muropeptide 16a. (C) MS2 obtained from targeting precursor ion 1246.5609 $[M+H]^{+2}$.

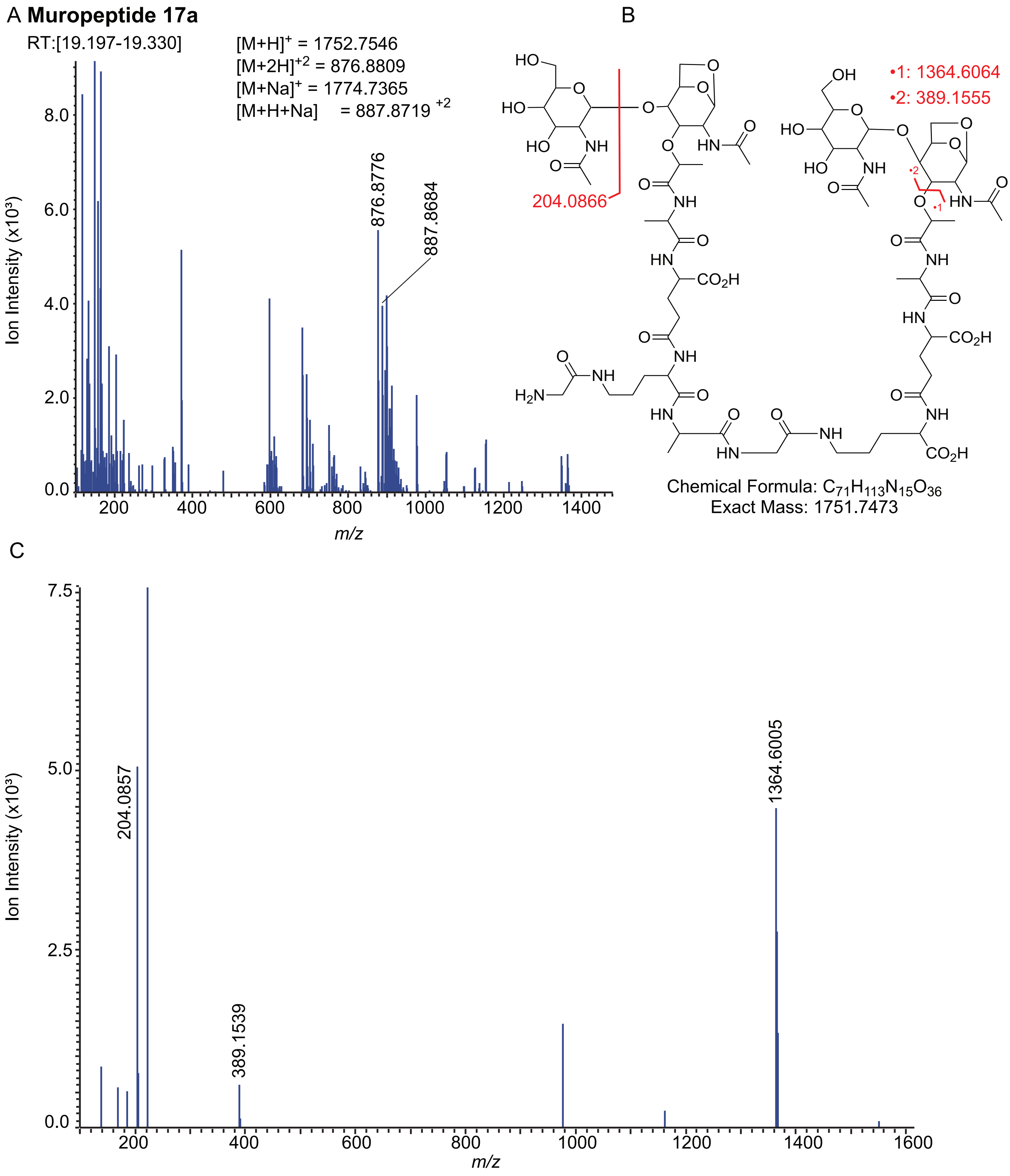

Supplemental Figure 27: (A) MS1 spectra generated from scanning retention times of 19.197-19.330 minutes. (B) The structure of muropeptide 17a. Cleavages with resulting *m/z* fragments are shown in red. (C) MS2 obtained from targeting precursor ion 876.87755 $[M+H]^{+2}$. Red fragments generated in B correspond to observed MS2 fragments in C.

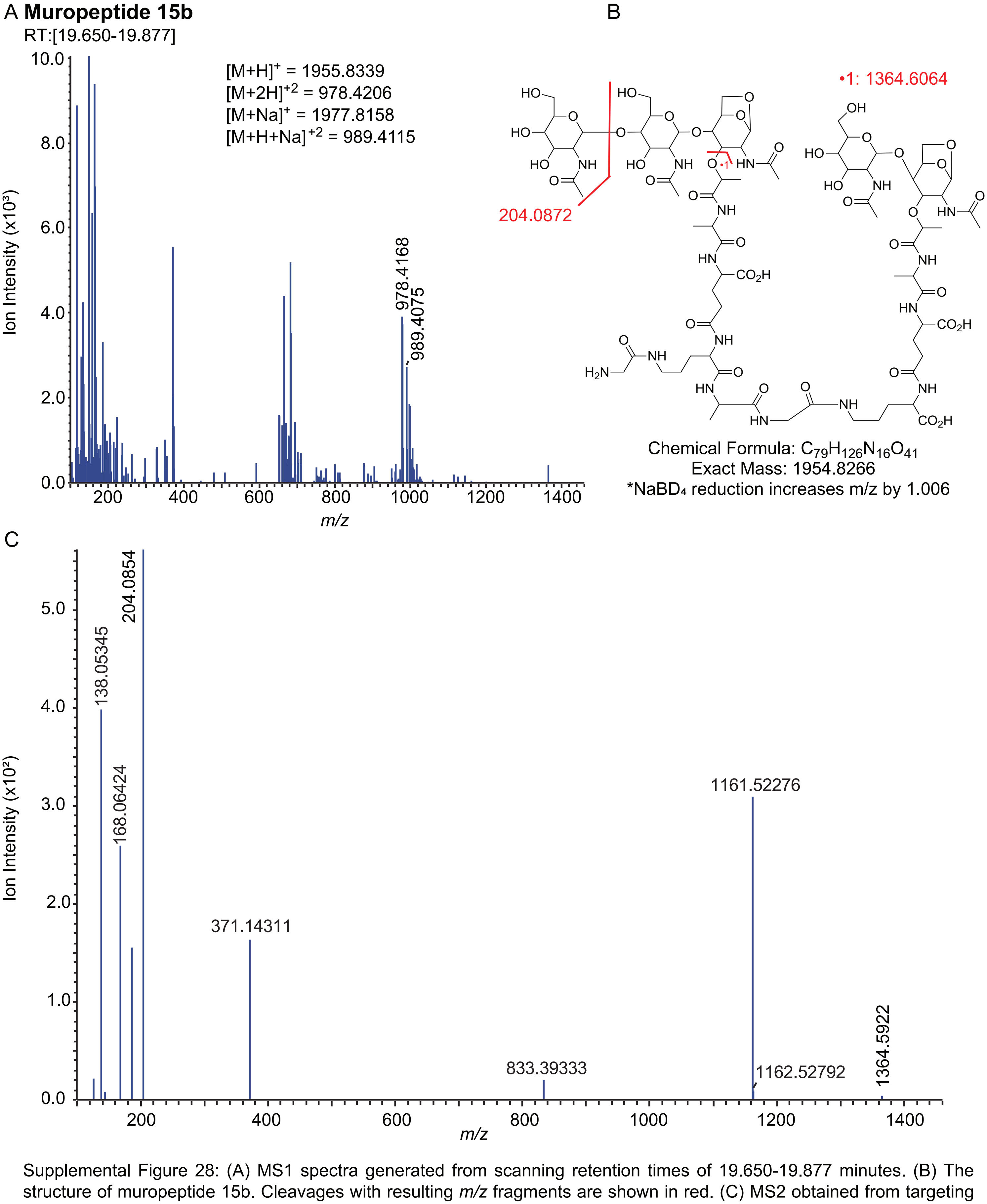

Supplemental Figure 28: (A) MS1 spectra generated from scanning retention times of 19.650-19.877 minutes. (B) The structure of muropeptide 15b. Cleavages with resulting *m/z* fragments are shown in red. (C) MS2 obtained from targeting precursor ion 978.4168 $[M+H]^{+2}$. Red fragments generated in B correspond to observed MS2 fragments in C.

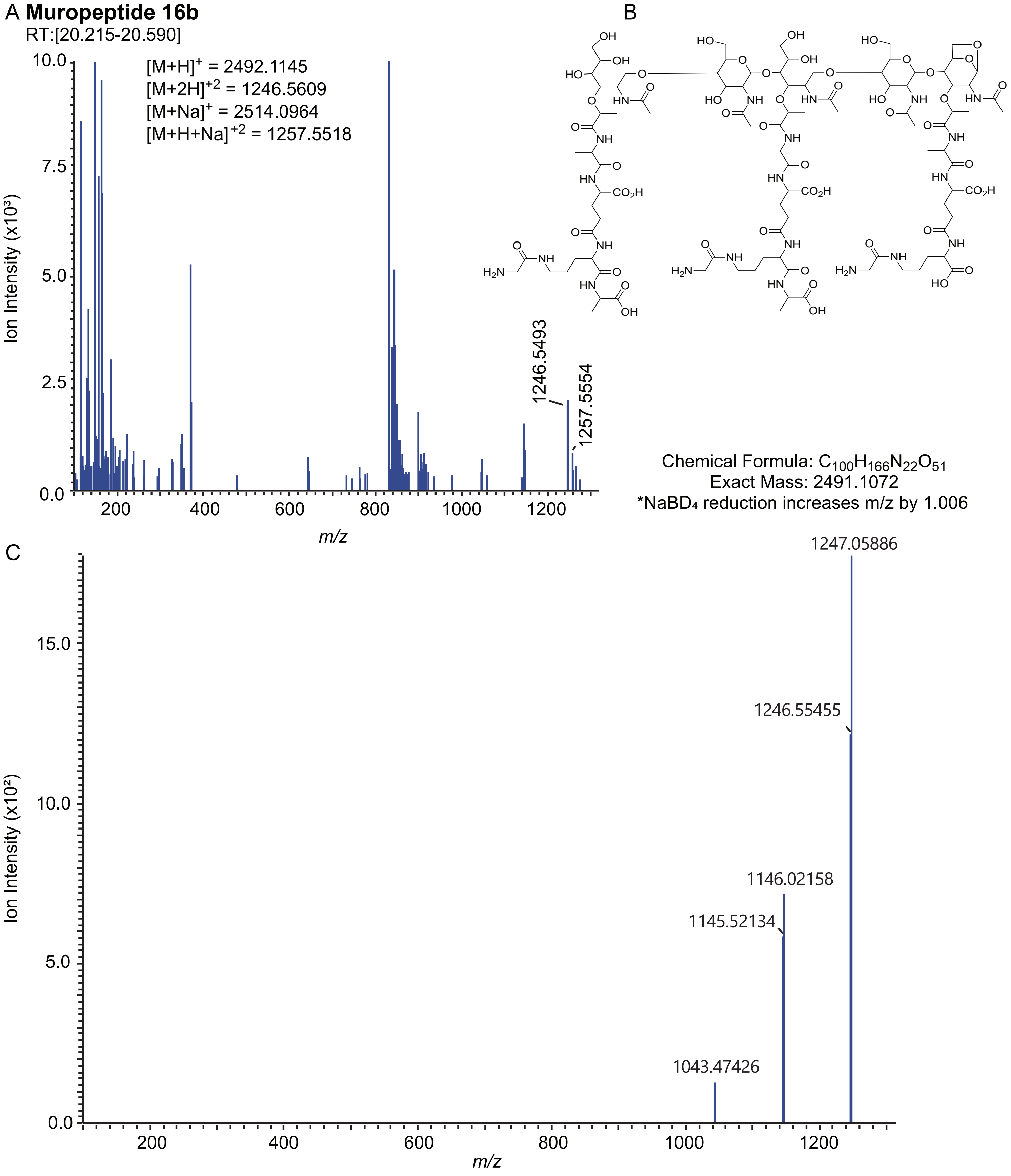

Supplemental Figure 29: (A) MS1 spectra generated from scanning retention times of 20.215-20.590 minutes. (B) The structure of muropeptide 16b. (C) MS2 obtained from targeting precursor ion 1246.5609 $[M+H]^{+2}$.

A **Muropeptide 17b**

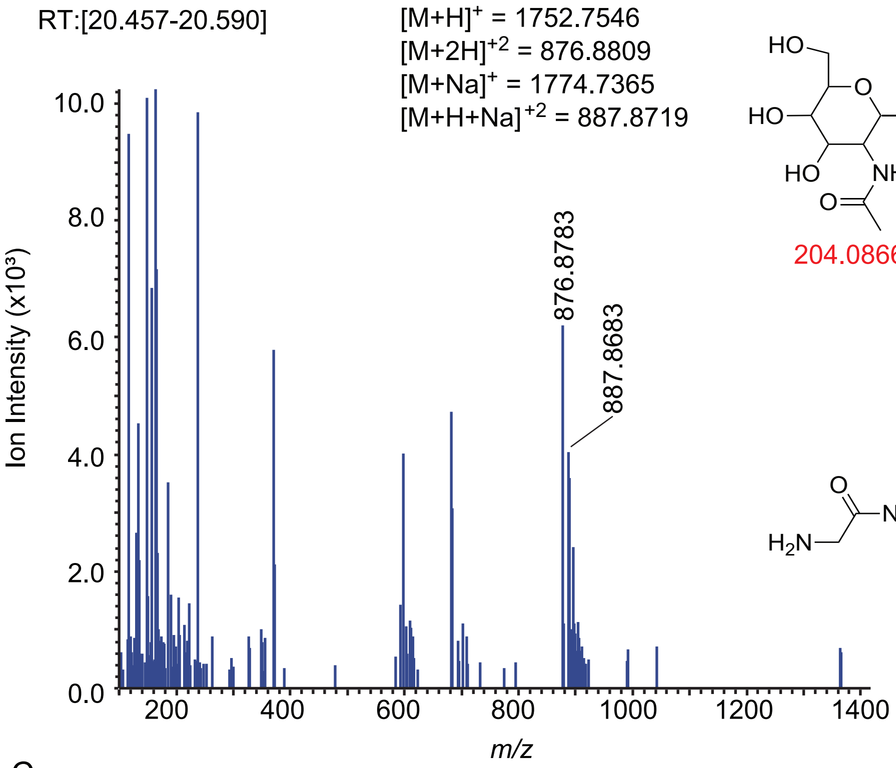

B

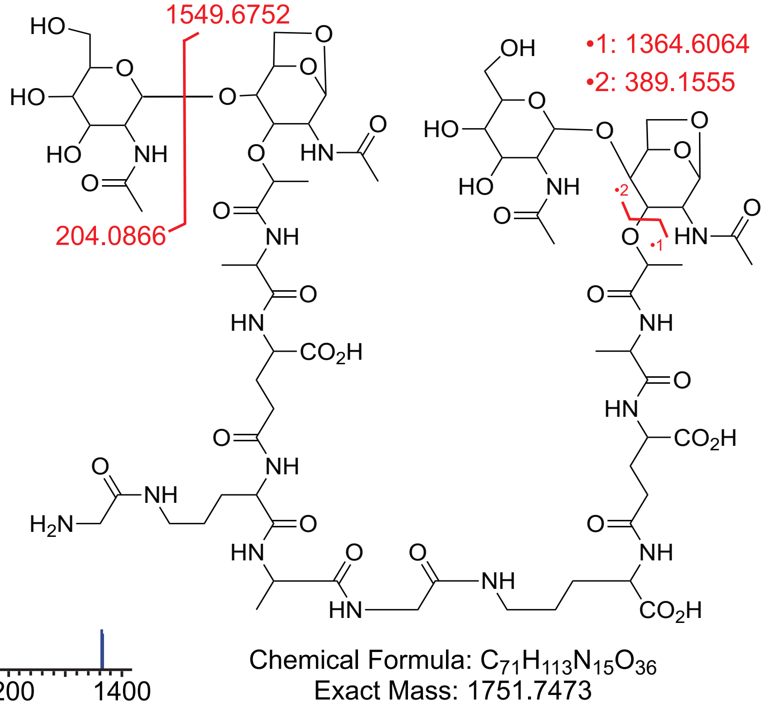

C

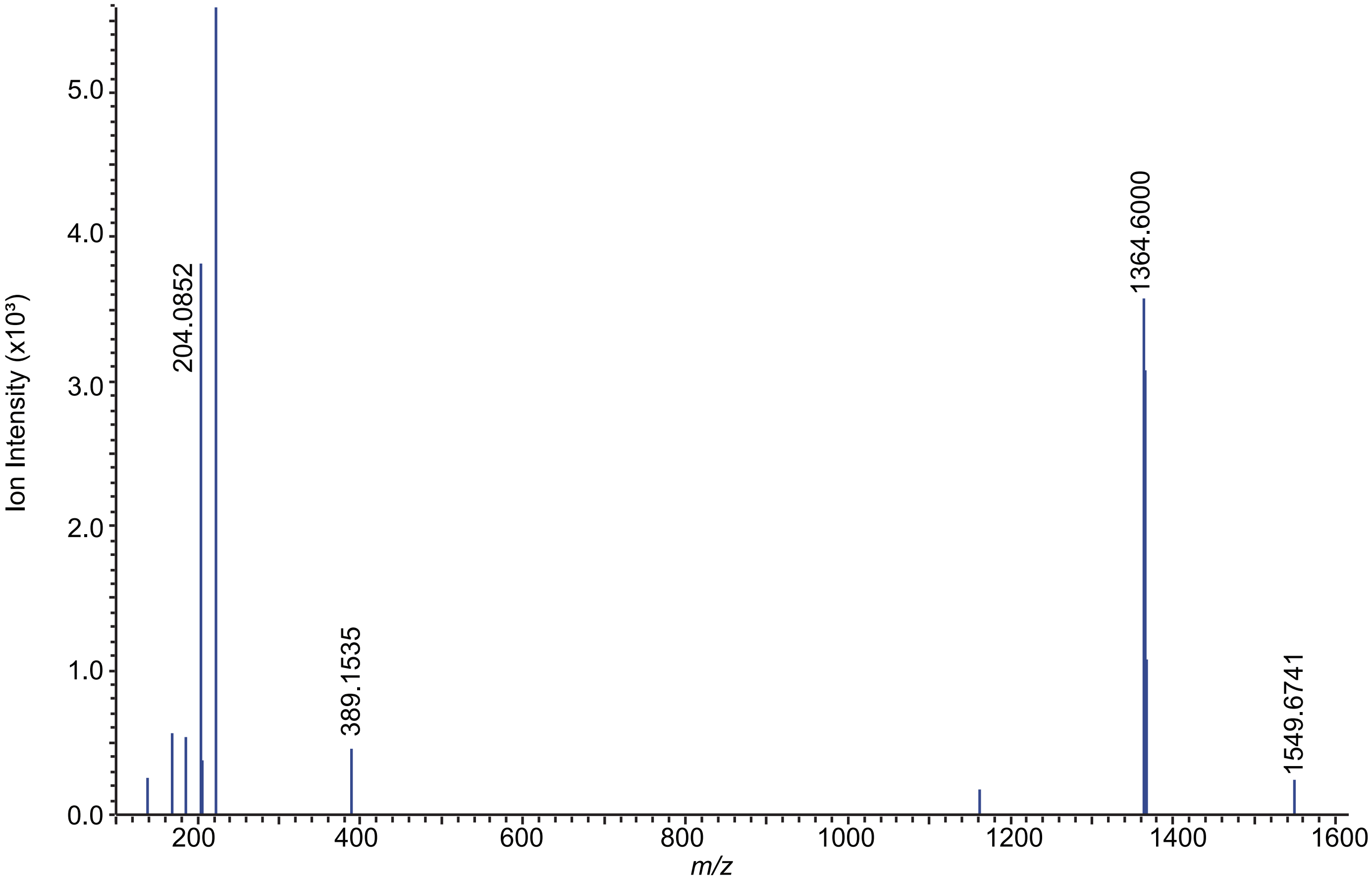

Supplemental Figure 30: (A) MS1 spectra generated from scanning retention times of 20.457-20.590 minutes. (B) The structure of muropeptide 17b. Cleavages with resulting *m/z* fragments are shown in red. (C) MS2 obtained from targeting precursor ion 876.87755 $[M+H]^{+2}$. Red fragments generated in B correspond to observed MS2 fragments in C.

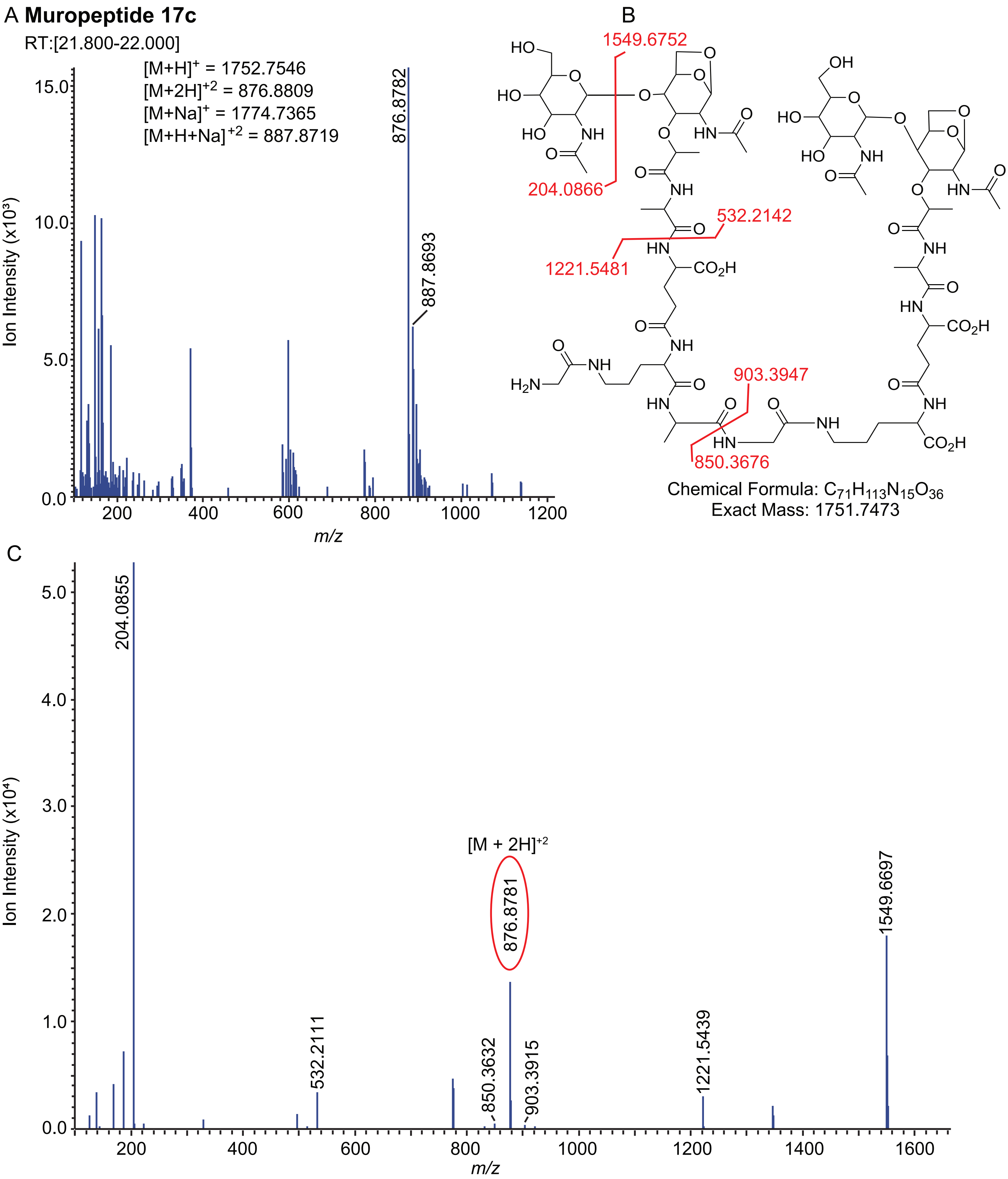

Supplemental Figure 31: (A) MS1 spectra generated from scanning retention times of 21.800-22.000 minutes. (B) The structure of muropeptide 17c. Cleavages with resulting *m/z* fragments are shown in red. (C) MS2 obtained from targeting precursor ion 876.87755 $[M+H]^{+2}$. Red fragments generated in B correspond to observed MS2 fragments in C.

Supplemental Table 4: A comparison of plasmid content in 5A11, 5A3, 5A3 N.I., and 5A3/*chbC B. burgdorferi* strains.

Plasmid	5A11	5A3	5A3 N. I.	5A3/*chbC*
cp9	+	-	-	-
cp26	+	+	+	+
cp32-1	+	+	+	+
cp32-3	+	+	+	+
cp32-4	+	+	+	+
cp32-6	+	+	+	+
cp32-7	+	+	+	+
cp32-8	+	+	+	+
cp32-9	+	+	+	+
lp5	+	-	-	-
lp17	+	+	+	+
lp21	+	+	-	+
lp25	+	+	-	+
lp28-1	+	+	-	+
lp28-2	+	+	+	+
lp28-3	+	+	+	+
lp28-4	+	+	+	+
lp36	+	+	-	+
lp38	+	+	+	+
lp54	+	+	+	+
lp56	+	+	+	+

Supplemental Table 5: In addition to the clean deletion of *chbC*, 5A3/*chbC* had the following single nucleotide polymorphisms, relative to 5A3.

Location	Position	Mutation	Annotation	Gene
lp28-1	27,425	S141S (AG<u>T</u>→AG<u>C</u>)	variable surface antigen VlsE	BB_RS05840
lp28-1	37,433	A144D (G<u>C</u>T→G<u>A</u>T)	variable surface antigen VlsE	BB_RS05840
lp28-1	27,897	D299N (<u>G</u>AT→<u>A</u>AT)	variable surface antigen VlsE	BB_RS05840
lp28-1	27,901	G300D (G<u>G</u>T→G<u>A</u>T)	variable surface antigen VlsE	BB_RS05840
lp28-1	27,910	E303G (G<u>A</u>G→G<u>G</u>G)	variable surface antigen VlsE	BB_RS05840

A

Feature Name	Retention Time (min)	# of Peaks
C01	13.34	31
C02	17.01	23
C03*°	9.08	17
C04	11.61	13
C05	17.83	12
C06	3.10	12
C09	3.10	9
C10	12.71	8
C11*	15.86	7
C12	16.45	7
C13	13.34	6
C14	13.35	6
C15	13.35	6
C16	3.10	6
C17	1.82	6
C18	16.45	5
C19	17.01	5
C20	17.82	3
C21	13.34	3
C22	13.34	3
C23	12.80	3
C24	5.74	3
C25	20.38	2
C26	19.15	2
C27	16.02	2
C28	13.34	2
C29	12.71	2
C30	13.03	2
C31	12.78	2
C36	3.09	2
C37	3.11	2

Supplementary Table 6: A) Features generated i RamCluster used for the comparison of G-G-anhM content in peptidoglycan from A3 and A3/*chbC* *B. burgdorferi*. Each feature is composed of a retention time and at least two peaks that correspond to an individual *m/z*. All features listed were used in the analysis. Features designated with an asterisk contain peaks corresponding to G-G-anhM. Features not listed (feature c07, c08, and c32-c35) were manually omitted — they represented adducts and redundancies that were not filtered out by RamClustR. A further breakdown of the peaks that compose each feature can be found in Supplemental Dataset 1. B) Relative comparison of G-G-anhM intensity in 5A3 and 5A3/*chbC* *B. burgdorferi*. All features were divided into two groups: G-G-anhM (features C03 & C11, designated by asterisk) or G-M (all other features).

°Feature C03 contained peaks corresponding to two separate muropeptides that had nearly identical retention times — one G-G-anhM and one G-M. Peaks within C03 were manually assigned as G-G-anhM or G-M depending on the muropeptide they correspond to. A further breakdown of which peaks in C03 correspond to G-G-anhM can be found in Source Data.

B

Strain	Total G-G-anhM Intensity	Total G-M Intensity	% G-G-anhM in Total PG	% G-G-anhM Reduction Relative to 5A3
5A3	68977	4813717	1.43	–
5A3/*chbC*	47423	5249974	0.90	37.1

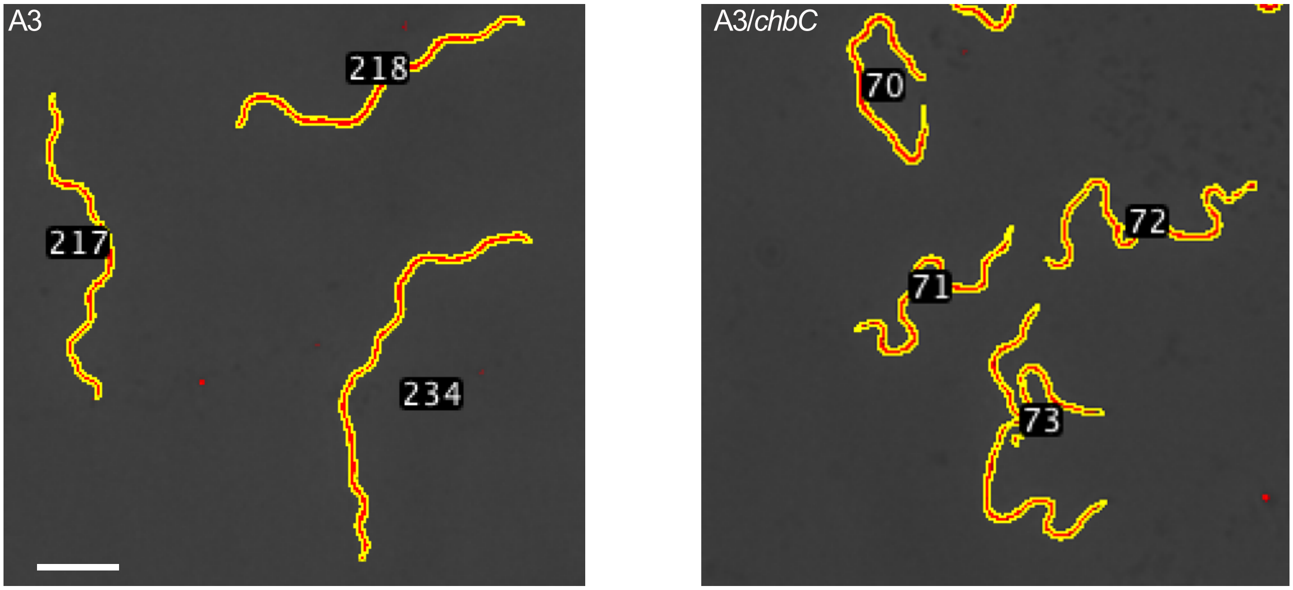

Supplemental Figure 32: Thresholds applied to phase-contrast micrographs containing *B. burgdorferi* A3 (left) and A3/*chbC* (right). Thresholds were generated using Fiji. Scale bars are 5 μm.
