# Supplementary material for: The unusual cell wall of the Lyme disease spirochaete Borrelia burgdorferi is shaped by a tick sugar
Source: Nat Microbiol. 2021 Nov 24;6(12):1583–92. doi: 10.1038/s41564-021-01003-w (PMC8612929; doi:10.1038/s41564-021-01003-w)
Supplement: Supplementary file 4 — Peer Review Information [file 41564_2021_1003_MOESM4_ESM.pdf]

Peer Review Information

Journal: Nature Microbiology
Manuscript Title: NMICROBIOL-21020523D
Corresponding author name(s): The unusual cell wall of the Lyme disease spirochaete Borrelia burgdorferi is shaped by a tick sugar

Reviewer Comments & Decisions:

Decision Letter, initial version:

Dear Professor Jutras

Thank you for submitting your Brief Communication entitled "A tick-derived sugar shapes the unusual peptidoglycan cell-wall of the Lyme disease spirochete" for consideration. Although I found the manuscript interesting, I do not think it is suitable for external peer review, at least in its present form.

In short, to be suitable for peer review for our journal, we would encourage you to revise your manuscript before we reconsider it.

1. The Brief communication format is not helpful in reading your paper -- so my recommendation is to reformat using the Article format as a template. This means adding back in the methods to the main text, ensuring there are 6-8 main text figures that underpin the main claims (I couldn't see figure 2 for example in my version) and adding in some subheadings. I hope these cosmetic alterations won't take you very long.

2. You carried out WGS on three different clones: but were any of the strain you sequenced the one you actually did your experiments on? Wasn't quite clear to me and needs to be clear in the paper itself. Didn't you use parental strain A3 and if so why didn't you sequence the passaged strain you have been using in your experiments (I now this bacterium grows slowly and can see the concern but I felt the absence of this sequence seemed odd -- did I overlook it?).]

3. I felt less convinced by the arguments about where the source of the subunits is and which enzymatic pathway in B. burdorferi was being utilized. Is there only one chitobiose transporter in the genome? What were the growth conditions under which you looked at WT and mutant cell walls? If chitin is the only source of the cell wall components how come the mutation wasn't lethal? More description is required of why you did those experiments and whether there is substantial additional work needed would be helpful.

4. How representative of the genus/spirochete lineage are the three strains for which you provided WGS and cell wall analyses? There are many spirochetes. Similarly what about other insect borne bacteria? Any evidence of a similar type of cell wall?

5. Links to motility: you need to better justify the sample size (300 single cells). This part could be very interesting and could receive more focus but you should ensure the conclusions do not overreach these data.

If you do not want to revise without review, I would recommend that you transfer this manuscript to Nature communications but please note that I have not consulted their editorial team.

If submitting the revised version of your manuscript, please pay close attention to our href="https://www.nature.com/nature-research/editorial-policies/image-integrity">Digital Image Integrity Guidelines. and to the following points below:

-- that unprocessed scans are clearly labelled and match the gels and western blots presented in figures.
-- that control panels for gels and western blots are appropriately described as loading on sample processing controls
-- all images in the paper are checked for duplication of panels and for splicing of gel lanes.

Finally, please ensure that you retain unprocessed data and metadata files after publication, ideally archiving data in perpetuity, as these may be requested during the peer review and production process or after publication if any issues arise.

Please use the link below to submit a revised paper:

{redacted}

Nature Microbiology is committed to improving transparency in authorship. As part of our efforts in this direction, we are now requesting that all authors identified as 'corresponding author' on published papers create and link their Open Researcher and Contributor Identifier (ORCID) with their account on the Manuscript Tracking System (MTS), prior to acceptance. This applies to primary research papers only. ORCID helps the scientific community achieve unambiguous attribution of all scholarly contributions. You can create and link your ORCID from the home page of the MTS by clicking on 'Modify my Springer Nature account'. For more information please visit please visit www.springernature.com/orcid.

Yours sincerely

{redacted}

natureresearch

Decision Letter, first revision:

Dear Professor Jutras,

Thank you for your patience while your manuscript "A tick-derived sugar shapes the unusual peptidoglycan cell-wall of Borrelia burgdorferi, the Lyme disease spirochete" was under peer-review at Nature Microbiology. It has now been seen by 3 referees, whose expertise and comments you will find at the end of this email. Although they find your work of some interest, they have raised a number of concerns that will need to be addressed before we can consider publication of the work in Nature Microbiology.

In particular, you must address the comments of referee 1, who points out the limitations of swarm agar and suggests using time-lapsed darkfield microscopy in conjunction with cell tracking; you must address the comments of referee 2 who has substantive concerns about the AFM work reported in your paper, and you should ensure that it is very clear that all approproate controls have been accounted for in the growth experiments, as mentioned by reviewer 3 and indeed ensure the limitations are clear and that you have indeed proven that the sugar is tick-derived.

Should further experimental data, including the outcomes of the experiments I have highlighted in this letter, allow you to address these criticisms, we would be happy to look at a revised manuscript. You will also need to carefully consider the remaining comments and respond to them in a rebuttal and in the manuscript itself. Please do not concern yourself with length constraints at this point as we can allow up to 4,500 and 7 display items and we only worry about length

We are committed to providing a fair and constructive peer-review process. Please do not hesitate to contact us if there are specific requests from the reviewers that you believe are technically impossible or unlikely to yield a meaningful outcome.

We strongly support public availability of data. Please place the data used in your paper into a public data repository, if one exists, or alternatively, present the data as Source Data or Supplementary Information. If data can only be shared on request, please explain why in your Data Availability Statement, and also in the correspondence with your editor. For some data types, deposition in a public repository is mandatory - more information on our data deposition policies and available repositories can be found at https://www.nature.com/nature-research/editorial-policies/reporting-standards#availability-of-data.

Please include a data availability statement as a separate section after Methods but before references, under the heading "Data Availability". This section should inform readers about the availability of the data used to support the conclusions of your study. This information includes accession codes to public repositories (data banks for protein, DNA or RNA sequences, microarray, proteomics data etc…), references to source data published alongside the paper, unique identifiers such as URLs to data repository entries, or data set DOIs, and any other statement about data availability. At a minimum, you should include the following statement: "The data that support the findings of this study are available from the corresponding author upon request", mentioning any restrictions on availability. If DOIs are provided, we also strongly encourage including these in the Reference list (authors, title, publisher (repository name), identifier, year). For more guidance on how to write this section please

see:
http://www.nature.com/authors/policies/data/data-availability-statements-data-citations.pdf

If revising your manuscript:

* Include a “Response to referees” document detailing, point-by-point, how you addressed each referee comment. If no action was taken to address a point, you must provide a compelling argument. This response will be sent back to the referees along with the revised manuscript.

* If you have not done so already we suggest that you begin to revise your manuscript so that it conforms to our Brief Communication format instructions at http://www.nature.com/nmicrobiol/info/final-submission. Refer also to any guidelines provided in this letter.

* Include a revised version of any required reporting checklist. It will be available to referees (and, potentially, statisticians) to aid in their evaluation if the manuscript goes back for peer review. A revised checklist is essential for re-review of the paper.

When submitting the revised version of your manuscript, please pay close attention to our href="https://www.nature.com/nature-research/editorial-policies/image-integrity">Digital Image Integrity Guidelines. and to the following points below:

-- that unprocessed scans are clearly labelled and match the gels and western blots presented in figures.
-- that control panels for gels and western blots are appropriately described as loading on sample processing controls
-- all images in the paper are checked for duplication of panels and for splicing of gel lanes.

Finally, please ensure that you retain unprocessed data and metadata files after publication, ideally archiving data in perpetuity, as these may be requested during the peer review and production process or after publication if any issues arise.

Please use the link below to submit a revised paper:

{redacted}

Note: This url links to your confidential homepage and associated information about manuscripts you may have submitted or be reviewing for us. If you wish to forward this e-mail to co-authors, please delete this link to your homepage first.

Nature Microbiology is committed to improving transparency in authorship. As part of our efforts in this direction, we are now requesting that all authors identified as ‘corresponding author’ on published papers create and link their Open Researcher and Contributor Identifier (ORCID) with their account on the Manuscript Tracking System (MTS), prior to acceptance. This applies to primary research papers only. ORCID helps the scientific community achieve unambiguous attribution of all scholarly

contributions. You can create and link your ORCID from the home page of the MTS by clicking on ‘Modify my Springer Nature account’. For more information please visit please visit www.springernature.com/orcid.

If you wish to submit a suitably revised manuscript we would hope to receive it within 6 months and ideally sooner given that you mentioned competition (keep me posted). If you cannot send it within this time, please let us know. We will be happy to consider your revision, even if a similar study has been accepted for publication at Nature Microbiology or published elsewhere (up to a maximum of 6 months).

In the meantime we hope that you find our referees' comments helpful.

Yours sincerely,

{redacted}

****
Reviewer Expertise:

Referee #1: Spirochete-tick interaction
Referee #2: Cell shape
Referee #3: Lyme disease and spirochetes

Reviewer Comments:

Reviewer #1 (Remarks to the Author):

In their submitted manuscript, DeHart et al address several fascinating aspects of Lyme disease spirochete physiology, namely, how does the bacteria acquire (or synthesize) the building blocks for its unusual peptidoglycan and, more importantly, how the bacterium alters its PG as it cycles between ticks and mammals. Overall, the paper is well written and the results largely support the authors conclusions. The work will be of substantial interest of the field. However, I have two major concerns, one experimental and one conceptual, both of which are detailed below.

First, Swarm assays on semi-solid medium typically are used to examine chemotaxis rather than motility/flagellar motor functionality. To more accurately assess the relationship between flagellar motor function and PG, the authors are strongly encouraged to compare their WT and mutant strains using time-lapsed darkfield microscopy in conjunction with cell tracking to should expand their analyses of motility (i.e., translational speed, pausing and flexing).

Secondly, the authors need to do more to relate their findings to: (i) prior studies related to physiological and nutritional acquisition/stress during tick feeding; (ii) spirochete physiology and (iii) the enzootic cycle. While these points are touched on in the text, there is not sufficient information for readers to appreciate the significance of the author’s findings. In its current form, a reader might assume that the ratio of GlcNAc-GlcNAc to GlcNAc-MurNAc is a constant.

The manuscript focuses (rightfully and logically) on utilization of chitobiose as a precursor for PG. Many prior studies point to the Chb system as a means of taking advantage of chitobiose as an

alternative carbon source for energy as well as PG. Where the text seems to fall short is putting these data in the context of the enzootic cycle. For instance:

In addition to the Chb system, B burgdorferi encodes transporters (MalX1, MalX2?) with the potential to import GlcNAc. How might the PG vary between tick (abundant Chb) and mammal (no Chb, limited GlcNAc)? Do spirochetes vary their PG in these two milieus in these two niches? What are the implications for motility and dissemination in ticks vs mammals? Prior studies suggest that following (early) hematogenous dissemination, spirochete essentially set up residence in in a given tissue and then remain local. Perhaps this is due to decreased ability (torque) to penetrate intercellular spaces as their PG switches from Tick-PG to Mammal-PG?

The manuscript focuses almost exclusively on the relationship between PG and motility. While this Reviewer is mindful of space limitations, the authors should consider alternative possibilities for why the spirochete may need to alter its PG in ticks. Prior studies by Sze et al and Bontemps-Gallo et al. suggest that starvation for Chitobiose (due to reduced expression of ChbC) leads to weakened PG and an inability to maintain the cell envelope, particular in response to transient increases in osmolality during tick feeding. Transient changes in PG composition may be are necessary to withstand the blood meal; this feature would be separate from motility. The implication is that changes in PG composition are a transient rather than general feature. Once in the mammal, GlcNAc becomes more limiting and the ratio of GlcNAc-GlcNAc to GlcNAc-MurNAc is shifted more towards the latter (providing an advantage with respect to lowering inflammation?).

How does the mOsm levels used in the stress tests perform here compare with those spirochetes encounter in feeding ticks as predicted by Bontemps-Gallo et al.? What was the reasoning for adding lysozyme as well as NaCl? Starvation for chitobiose should be sufficient to induce blebbing/lysis in a ChbC mutant.

Did the authors visualize the "stressed" cells by simple darkfield prior to recovery? Or alternatively, making their WT and ChbC mutant fluorescent and imaging using epifluorescence microscopy? Fixation and/or agarose embedding may introduce artifacts and/or alter morphology compared to liquid (darkfield) imaging of unfixed organism.

Minor comments:
What is the likelihood that this PG alteration occurs in other bacteria genera?

Ln 47: Some (almost certainly) will disagree with characterization of early Lyme symptoms as being "relatively innocuous". Suggest revising.

Ln 77: suggest "time consuming" rather than "long".

Ln 184: In the methods it is clear that they are using a function called Roundness, but the context is not as clear in the Results.

Reviewer #2 (Remarks to the Author):

This manuscript reports the interesting discovery that Borrelia burgdorferi utilizes a unique peptidoglycan component, GlcNAc-GlcNAc, located at the terminus of the glycan strands, which is at least partially derived from chitobiose from its tick host. The authors show, albeit fairly crudely, that mutants that cannot import GlcNAc-GlcNAc, have altered morphology and motility. AFM images show that the PG sacculus is altered in these mutants, which supports the claim that failure to incorporate as much GlcNAc-GlcNAc into the PG is responsible for alterations that impact the motility and morphology. AFM measurements are futher used to suggest that the GlcNAc-GlcNAc reduced mutants have stiffer cell walls than wildtype. As the motility of these bacteria is crucial for virulence and pathogenesis, understanding aspects that impact this is important. Overall, then, I feel that the primary finding is interesting and worthy of publication; however, I feel that in its current state, the manuscript does not sufficiently describe the methods/results to be acceptable for publication.

The authors show use Mass Spec and NMR to analyze the glycan composition. These techniques lie outside my expertise to critically evaluate, so I cannot comment on the likelihood that these procedures were carried out adequately and will leave that to other reviewers to comment on.

Primary Comments

1. The major concern that I have with the methods/results deals with the force measurements from the AFM. There are a number of concerns here, most notably, Fig. 6A labels the bottom panels as force (with units of N/m^2). I believe that what the authors mean is Young's modulus, not force, as this is what is typically measured with AMFM mode. If this is correct, then I am very concerned with the value that is being found. The mutant values are coming out to be around 2.0 GPa, which is close to the Young's modulus of mica that I was able to find (5.4 GPa), while most measurements of the Young's modulus of PG are in the range of 0.1-0.2 GPa. There are a couple possible explanations for this discrepancy. One the PG that is being measured is quite thin, the Hertz model assumes that the elastic layer is semi-infinitely thick. Thereby giving a highly erroneous result. It also could be that drying the PG leads to large discrepancies in the moduli, or some combination of these two. What is being measured needs to be made clear and there also needs to be some control to guarantee that the actual stiffness of the PG is being measured.

2. The AFM "force" measurements also do not seem to be consistent with the AFM images that are shown. The frayed appearance of the PG in the mutant cells would seem to suggest that the PG is weaker (less cohesive) than the smoother PG in the wildtype cells. Nor do the "force" measurements agree with the authors description of what is going on. Stiffer PG should be better able to withstand stresses from the rotating flagella than softer PG. In regard to this, the authors should probably cite Harman, et al. Biophys. J. 112: 746-754 (2017). In this paper, the authors show that vancomycin treatment makes the cell wall softer and reduces motility, which would suggest to me that the GlcNAc-GlcNAc mutant is likely softer, not stiffer, than the wildtype. (And also consistent with the change in morphology that is observed, which may be consistent with the work presented in Ref. 24). In addition, the text on the torque wringing the bacteria out like a wet towel and requiring flexible but strong PG (lines 174-178) is not exactly accurate. More flexible is more akin to softer, but to withstand the torques and forces in order to prevent the cell from being wrung like a towel, requires that the cell wall should be stiffer.

3. Minor point: "our" in line 68 is not needed

3.

Reviewer #3 (Remarks to the Author):

This manuscript characterizes a novel feature of peptidoglycan in B. burgdorferi, which harbors GlcNAc-GlcNAc disaccharides rather than GlcNAc linked to MurNac. Using a B. burgdorferi strain harboring a mutation of the chitobiose transporter gene chbC, the authors show the peptidoglycan from this strain harbors less GlcNAc-GlcNAc disaccharides resulting in defects in morphology, motility, osmotic stress resistance, and cell envelope defects.

Major comments:

1) The authors presume chitobiose is present in BSK II culture medium, however, there is no direct experimental evidence provided in the manuscript to support this claim. Additionally, there is no conclusive evidence from the literature that BSK II contains chitobiose (Tilly K, Elias AF, Errett J, Fischer E, Iyer R, Schwartz I, Bono JL, Rosa P. J Bacteriol. 2001; Rhodes RG, Atoyan JA, Nelson DR. BMC Microbiol. 2010). However, there is evidence that GlcNAc-free BSK II media supplemented with chitobiose is capable of supporting the growth of B. burgdorferi, presumably through transport by the ChbABC transporter.
2) The authors state in the abstract and line 230, that the presence of GlcNAc-GlcNAc disaccharides in the peptidoglycan of B. burgdorferi represents a paradigm shift in our understanding of peptidoglycan architecture. This statement is a bit of a reach. Rather than a paradigm shift, this finding represents yet another example of a surprisingly unique feature of B. burgdorferi that is analogous to its iron and thiamine-free lifestyle. Further experimental evidence showing this unique feature of peptidoglycan is conserved in other Borrelia species (B. hermsii, B. miyamotoi, etc.) or other genera of spirochetes (Treponema, Leptospira), could likely be argued to be a true paradigm shift for our understanding of peptidoglycan structure.

Minor comments:
1) The title of this manuscript is a bit misleading stating that a "tick-derived sugar shapes the unusual peptidoglycan cell-wall of B. burgdorferi . . ." as the experiments presented in this manuscript were carried out in BSK II media.
2) It appears the experiments shown in Figs 4-6 were carried out in BSK II complete media (with GlcNAc) with cells grown to early log phase (~10^7 cells/ml). Based on Tilly K, et. al. 2001, the chbC mutant presumably has the same growth kinetics as wild-type B. burgdorferi. Are the genes encoding the chbABC transporter expressed in early log phase? Were these experiments ever carried out in BSK II media deficient in components that are speculated sources of chitobiose (yeastolate)?

Author Rebuttal, first revision:

Reviewer #1 (Remarks to the Author):

In their submitted manuscript, DeHart et al address several fascinating aspects of Lyme disease spirochete physiology, namely, how does the bacteria acquire (or synthesize) the building blocks for its unusual peptidoglycan and, more importantly, how the bacterium alters its PG as it

cycles between ticks and mammals. Overall, the paper is well written and the results largely support the authors conclusions. The work will be of substantial interest of the field. However, I have two major concerns, one experimental and one conceptual, both of which are detailed below.

First, Swarm assays on semi-solid medium typically are used to examine chemotaxis rather than motility/flagellar motor functionality. To more accurately assess the relationship between flagellar motor function and PG, the authors are strongly encouraged to compare their WT and mutant strains using time-lapsed darkfield microscopy in conjunction with cell tracking to should expand their analyses of motility (i.e., translational speed, pausing and flexing).

RESPONSE: We respectfully disagree with the notion that swarm assays are used to study chemotaxis rather than motility in the spirochete field. We have provided 20 references below [1-20] in which swarm plates assays were used to assess *Borrelia burgdorferi* motility. The references provided were published in highly reputable journals and all of the work come from experts (over 300 Lyme publications collectively) in *Borreliae* and *B. burgdorferi* motility (Nyles Charon, Md Motaleb, Chris Li, Jorge Benach, Steve Norris, etc). We also provide additional citations that support the use of swarm plates to measure motility in other spirochetes[21-24]. Furthermore, dark-field tracking experiments do not truly represent the conditions optimal for motility since there is no substrate available to propel the bacterium relative to a more viscous medium[25]. Finally, we note that the *chbC* mutant is still motile, just impaired, which we have stressed in the text (see new lines 195).

Secondly, the authors need to do more to relate their findings to: (i) prior studies related to physiological and nutritional acquisition/stress during tick feeding; (ii) spirochete physiology and (iii) the enzootic cycle. While these points are touched on in the text, there is not sufficient information for readers to appreciate the significance of the author's findings. In its current form, a reader might assume that the ratio of GlcNAc-GlcNAc to GlcNAc-MurNAc is a constant.

RESPONSE: We were hesitant to speculate too much, but these comments (and those below) are all fantastic points. We have cautiously added many of these points into the revised discussion section. See details as they relate to each point below.

The manuscript focuses (rightfully and logically) on utilization of chitobiose as a precursor for PG. Many prior studies point to the Chb system as a means of taking advantage of chitobiose as an alternative carbon source for energy as well as PG. Where the text seems to fall short is putting these data in the context of the enzootic cycle. For instance:

In addition to the Chb system, B burgdorferi encodes transporters (MalX1, MalX2?) with the

potential to import GlcNAc. How might the PG vary between tick (abundant Chb) and mammal (no Chb, limited GlcNAc)? Do spirochetes vary their PG in these two milieus in these two niches? What are the implications for motility and dissemination in ticks vs mammals? Prior studies suggest that following (early) hematogenous dissemination, spirochete essentially set up residence in in a given tissue and then remain local. Perhaps this is due to decreased ability (torque) to penetrate intercellular spaces as their PG switches from Tick-PG to Mammal-PG?

RESPONSE: At this point, it is impossible to know how PG composition/architecture varies in a vertebrate host since there is not enough material to test. Since there is seemingly no 'pressure' in culture to produce glycan strands with G-G-anhM, and yet *Borrelia* strains and species contain the trisaccharide, we favor a scenario where this is an inherent, programmed feature with a dual mechanism of production: chitobiose import in ticks, G-G-anhM synthesis in vertebrate hosts. We appreciate this intriguing point and have provided new discussion items focused around this idea. Many thanks for the suggestions.

The manuscript focuses almost exclusively on the relationship between PG and motility. While this Reviewer is mindful of space limitations, the authors should consider alternative possibilities for why the spirochete may need to alter its PG in ticks. Prior studies by Sze et al and Bontemps-Gallo et al. suggest that starvation for Chitobiose (due to reduced expression of ChbC) leads to weakened PG and an inability to maintain the cell envelope, particular in response to transient increases in osmolality during tick feeding. Transient changes in PG composition may be are necessary to withstand the blood meal; this feature would be separate from motility. The implication is that changes in PG composition are a transient rather than general feature. Once in the mammal, GlcNAc becomes more limiting and the ratio of GlcNAc-GlcNAc to GlcNAc-MurNAc is shifted more towards the latter (providing an advantage with respect to lowering inflammation?).

RESPONSE: I'm not sure if reduced *chbC* expression (Sze et al., Bontemps-Gallo et al.) constitute chitobiose starvation (see related point below, 384-406 of this document). Further, it seems as though the *chbC* mutant strain is capable of completing the enzootic cycle[26]. Those points notwithstanding, we have added two additional paragraphs into the discussion with these ideas in mind. Thanks for the enthusiastic suggestions, they're great!

How does the mOsm levels used in the stress tests perform here compare with those spirochetes encounter in feeding ticks as predicted by Bontemps-Gallo et al.? What was the reasoning for adding lysozyme as well as NaCl? Starvation for chitobiose should be sufficient to induce blebbing/lysis in a ChbC mutant.

RESPONSE: We have measured the mOsm of our osmotic stress conditions, which we previously optimized and determined in a recently published study[27]. To answer your question, our NaCl stress conditions (544 mOsm) fall within the higher range of what *B. burgdorferi* can tolerate (250-650 mOsM)[28] and very close to tick feeding (600 mOsm)[28]. We have included this information in revised manuscript figure legend (Fig. 4) and the corresponding methods.

Both stress tests were performed independently. Salt tests osmotic pressure, while lysozyme PG continuity. Together, they are good indicators of overall PG function.

Did the authors visualize the “stressed“ cells by simple darkfield prior to recovery? Or alternatively, making their WT and ChbC mutant fluorescent and imaging using epifluorescence microscopy? Fixation and/or agarose embedding may introduce artifacts and/or alter morphology compared to liquid (darkfield) imaging of unfixed organism.

RESPONSE: There might be some confusion. Determining CFU, after stress, is an accurate and robust way to evaluate the functional consequences of having 1) less PG and 2) less G-G-anhM upon cell envelop stress.

This seems to be unrelated to the point above. But assuming you’re referring to our morphological analysis, fixation preserves the morphology of a bacterium. Agarose embedding was not method we considered, nor was it used in our studies. The fixed bacteria were simply visualized on agarose pads. Visualizing swimming cells by darkfield microscopy is not a quantitative assessment of morphology—they are constantly moving and as a result so too is the helical pitch. These types of analysis would be qualitative observations and are performed on a few cells rather than hundreds (see details in Fig. 5 legend).

Minor comments:

What is the likelihood that this PG alteration occurs in other bacteria genera?

RESPONSE: Excellent point. See our response to reviewer 3 comment on this similar topic.

Ln 47: Some (almost certainly) will disagree with characterization of early Lyme

symptoms as being “relatively innocuous”. Suggest revising.

RESPONSE: Good point, we’ve made the appropriate change. Thank you.

Ln 77: suggest “time consuming” rather than “long”.

RESPONSE: Change made, thanks.

Ln 184: In the methods it is clear that they are using a function called Roundness, but the context is not as clear in the Results.

RESPONSE: We have changed the wording of this section and added a sentence to further explain the analysis in the results sections (new lines 188-189). Thanks for pointing this out.

Reviewer #2 (Remarks to the Author):

This manuscript reports the interesting discovery that Borrelia burgdorferi utilizes a unique peptidoglycan component, GlcNAc-GlcNAc, located at the terminus of the glycan strands, which is at least partially derived from chitobiose from its tick host. The authors show, albeit fairly crudely, that mutants that cannot import GlcNAc-GlcNAc, have altered morphology and motility. AFM images show that the PG sacculus is altered in these mutants, which supports the claim that failure to incorporate as much GlcNAc-GlcNAc into the PG is responsible for alterations that impact the motility and morphology. AFM measurements are futher used to suggest that the GlcNAc-GlcNAc reduced mutants have stiffer cell walls than wildtype. As the motility of these bacteria is crucial for virulence and pathogenesis, understanding aspects that impact this is important. Overall, then, I feel that the primary finding is interesting and worthy of publication; however, I feel that in its current state, the manuscript does not sufficiently describe the methods/results to be acceptable for publication.

The authors show use Mass Spec and NMR to analyze the glycan composition. These techniques lie outside my expertise to critically evaluate, so I cannot comment on the likelihood that these procedures were carried out adequately and will leave that to other reviewers to comment on.

Primary Comments

1. The major concern that I have with the methods/results deals with the force measurements from the AFM. There are a number of concerns here, most notably, Fig. 6A labels the bottom panels as force (with units of N/m^2). I believe that what the authors mean is Young's modulus, not force, as this is what is typically measured with AMFM mode.

RESPONSE: You're absolutely correct, what is being measured is Young's modulus and the units being reported is a measure of force. Thank you for bringing this to our attention, a mistake on our part. We have corrected the figure and legend, but the data remain the same.

If this is correct, then I am very concerned with the value that is being found. The mutant values are coming out to be around 2.0 GPa, which is close to the Young's modulus of mica that I was able to find (5.4 GPa), while most measurements of the Young's modulus of PG are in the range of 0.1-0.2 GPa. There are a couple possible explanations for this discrepancy. One the PG that is being measured is quite thin, the Hertz model assumes that the elastic layer is semi-infinitely thick. Thereby giving a highly erroneous result. It also could be that drying the PG leads to large discrepancies in the moduli, or some combination of these two. What is being measured needs to be made clear and there also needs to be some control to guarantee that the actual stiffness of the PG is being measured.

RESPONSE: We appreciate your concerns as it relates to this point and one must always be careful when it comes to measuring materials on mica. Our AFM young's modulus measurements of wild-type *B. burgdorferi* PG are very close to those previously published for *E. coli* sacculi, analyzed under similar conditions[29]. We note, however, that it is difficult to make direct comparisons between different AFM data acquisition setups (imaging platforms, tips, calibration, etc.). This point has been made in the new discussion section.

As for the modulus of mica, we disagree. Recall that the heatmaps shown in Fig 6A are scaled to the relevant data in the image and not the total range of data collected. To address your concern, we went back to our original data sets and collected the raw measurements on mica alone (i.e., where the sacculi were not present and the mica appeared to be free of debris). In Fig. 1X we have graphed this data (n > 1600 points) for the reviewer as a control. Our Young's modulus measurements of mica are on average ~19.7 GPa (or 1.97 X10^9 N/m^2), which is more than 10 X higher than the average value calculated for the A3/*chbC* sacculi.

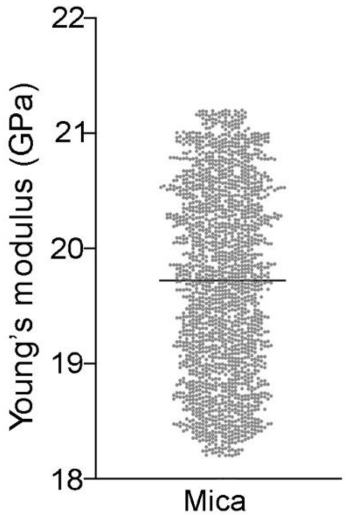

Figure X1. Young's modulus measurements of mica. To confirm that the thickness of PG sacculi were not interfering with Young's modulus measurements, we went back to our original data set and collected measurements from mica alone, or regions where sacculi were not present and the substrate seemed free of debris. Two samples were randomly chosen from each sample preparation, four total, and modulus measurements were compiled for each data point (*n* = 1860, average = 19.71 (+/-0.718)).

2. The AFM "force" measurements also do not seem to be consistent with the AFM images that are shown. The frayed appearance of the PG in the mutant cells would seem to suggest that the PG is weaker (less cohesive) than the smoother PG in the wildtype cells. Nor do the "force" measurements agree with the authors description of what is going on. Stiffer PG should be better able to withstand stresses from the rotating flagella than softer PG. In regard to this, the authors should probably cite Harman, et al. Biophys. J. 112: 746-754 (2017). In this paper, the authors show that vancomycin treatment makes the cell wall softer and reduces motility, which

would suggest to me that the GlcNAc-GlcNAc mutant is likely softer, not stiffer, than the wildtype. (And also consistent with the change in morphology that is observed, which may be consistent with the work presented in Ref. 24). In addition, the text on the torque wringing the bacteria out like a wet towel and requiring flexible but
strong PG is not exactly accurate. More flexible is more akin to softer, but to withstand the torques and forces in order to prevent the cell from being wrung like a towel, requires that the cell wall should be stiffer.

RESPONSE: Excellent points. First, it is difficult to compare the observational image data (Fig. 4A) with true biophysical measurements (Fig. 6), which is why we refrained from commenting in the discussion on this point. But overall continuity may not impart physical properties, one way or another. This is a very interesting observation though and something we are actively pursuing.

We have added the reference, as suggested by the reviewer, to a new section in the discussion involving these concepts, but we also want to make several comments regarding this work.

Harman et al. (Biophysical Journal, 2017)[30] test a mathematical model that suggests the morphology and motility of *Borrelia burgdorferi* are dependent on the flagellar/cell wall stiffness ratio. Their biophysical model predicts that swimming speed should increase with decreasing cell stiffness, i.e., motility increases as cell flexibility increases. Harman et al. also hypothesized that increasing cell wall stiffness would lead to increased rotational drag on periplasmic flagella. Increased rotational drag would, in turn, decrease the motility of *B. burgdorferi*. The authors tested this model by measuring the swimming speed of *B. burgdorferi* exposed to various concentrations of vancomycin — an antibiotic shown to gradually decrease cell wall stiffness over time. Harman et al. observed that in the presence of vancomycin *B. burgdorferi's* morphology suffered minor deviations and swimming speed decreased by ~15%. This data suggests an inverse relationship between motility and flexibility: when cell flexibility increases, motility decreases. These findings conflict with their biophysical model, and agreement is contingent on a new assumption: that periplasmic volume decreases as cell wall stiffness decreases. Contrary to the original hypothesis based on their previous findings and biophysical model, this new assumption suggests decreasing cell wall stiffness will increase flagellar rotational drag due to an accompanying decrease in periplasmic volume. However, the authors state this is a prediction that will need to be tested with Cyro-EM or other techniques capable of measuring the periplasmic space. The authors also reason that vancomycin may have

additional effects on the cell, outside of impacting cell wall stiffness, that could influence the agreement of their results and their model.

We demonstrate that 5A3/*chbC B. burgdorferi* incapable of incorporating Glc*N*Ac-Glc*N*Ac into their peptidoglycan are significantly less motile and less flexible that their wild type counterparts, among other findings. Relative to these 5A3/*chbC*, wild type *B. burgdorferi* are more flexible and more motile. Our findings agree with the biophysical model studied in Harman et al. without the need for additional assumptions: both suggest a direct relationship exists between flexibility and motility.

Good point and a poor analogy. We have removed the wet towel comparison.

3. Minor point: "our" in line 68 is not needed

RESPONSE: Thank you for catching this typo. We have removed 'our'

Reviewer #3 (Remarks to the Author):

This manuscript characterizes a novel feature of peptidoglycan in B. burgdorferi, which harbors GlcNAc-GlcNAc disaccharides rather than GlcNAc linked to MurNac. Using a B. burgdorferi strain harboring a mutation of the chitobiose transporter gene chbC, the authors show the peptidoglycan from this strain harbors less GlcNAc-GlcNAc disaccharides resulting in defects in morphology, motility, osmotic stress resistance, and cell envelope defects.

Major comments:

1) The authors presume chitobiose is present in BSK II culture medium, however, there is no direct experimental evidence provided in the manuscript to support this claim. Additionally, there is no conclusive evidence from the literature that BSK II contains chitobiose (Tilly K, Elias AF, Errett J, Fischer E, Iyer R, Schwartz I, Bono JL, Rosa P. J Bacteriol. 2001; Rhodes RG, Atoyan JA, Nelson DR. BMC Microbiol. 2010). However, there is evidence that GlcNAc-free BSK II

media supplemented with chitobiose is capable of supporting the growth of B. burgdorferi, presumably through transport by the ChbABC transporter.

RESPONSE: Thank you for bringing this back to our attention, it's something we thought about a great deal during this project's infancy. We cited the studies above because they both point out that it is highly likely that yeast extract present in the BSK-II culture medium contain chitobiose which, in our view, is generally accepted but not empirically proven. We have included the references that support this claim in this document[31-34] and added this information in the new text.

2) The authors state in the abstract and line 230, that the presence of GlcNAc-GlcNAc disaccharides in the peptidoglycan of B. burgdorferi represents a paradigm shift in our understanding of peptidoglycan architecture. This statement is a bit of a reach. Rather than a paradigm shift, this finding represents yet another example of a surprisingly unique feature of B. burgdorferi that is analogous to its iron and thiamine-free lifestyle. Further experimental evidence showing this unique feature of peptidoglycan is conserved in other Borrelia species (B. hermsii, B. miyamotoi, etc.) or other genera of spirochetes (Treponema, Leptospira), could likely be argued to be a true paradigm shift for our understanding of peptidoglycan structure.

RESPONSE: Our view is from the entire field of bacteriology, not spirochetes. PG is a universal feature of virtually all bacterial cells. It's organization and composition has been studied for more than 60 years. In fact, there are more than 10,000 papers analyzing the composition of PG from different bacteria. The major unifying theme in all studies is that glycan sugar stoichiometry does not change—every other sugar is a MurNAc with the lactyl moiety necessary for peptide assembly. This statement is made in dozens of reviews. Waldemar Vollmer, the undisputed world expert in this field (179 co-authored PG papers), agrees that this is unprecedented and truly a paradigm shift in PG organization. The latter stems from communications back and forth while we were preparing this manuscript (see acknowledgement section). Together, we feel comfortable making this statement and do not see it as a reach.

B. burgdorferi is, no doubt, weird. We have added some of the other surprising physiological anomalies into the revised discussion, at the request of the reviewer 1 and based on the comment above.

As for the conservation of GlcNAc-GlcNAc-anhMurNAc: this is an excellent point and something we have been actively investigating. **In our revised manuscript we address this question by including new data that provides the first high-resolution analysis of *Borrelia hermsii* PG (Fig 2)**. *B. hermsii* is, of course, a relapsing fever spirochete that is transmitted to humans by soft ticks. Consistent with our hypothesis, *B. hermsii* PG also contains appreciable levels of GlcNAc-GlcNAc-anhMurNAc (Fig. 2D). This was an exciting result, it makes our study more comprehensive, and we appreciate the suggestion!

High quality data has been published in terms of the chemical composition and organization of *Leptospira interrogans* PG[35]. The authors did not find any unexpected alterations to PG glycan sugar stoichiometry. We have carefully reviewed their data and agree. This is consistent with our hypothesis that chitobiose derived from the tick vector is an important component in this system. It further supports peculiarity of the *Borrelia* genus and will certainly be the focus of future studies. Thank you for this and all the comments above.

Minor comments:

1) The title of this manuscript is a bit misleading stating that a “tick-derived sugar shapes the unusual peptidoglycan cell-wall of B. burgdorferi . . .” as the experiments presented in this manuscript were carried out in BSK II media.

RESPONSE: We have discovered that *B. burgdorferi* PG glycans contains GlcNAc-GlcNAc. Ticks contain GlcNAc-GlcNAc[36,37] and when *B. burgdorferi* is unable to import *B. burgdorferi* GlcNAc-GlcNAc, level of PG GlcNAc-GlcNAc decrease. We understand the comment regarding studies being conducted in BSK II media and thus do not actually contain a ‘tick-derived sugar’ in the pure sense. We do not want to mislead the readers, so we have changed the title. Thank you.

2) It appears the experiments shown in Figs 4-6 were carried out in BSK II complete media (with GlcNAc) with cells grown to early log phase (~10^7 cells/ml). Based on Tilly K, et. al. 2001, the chbC mutant presumably has the same growth kinetics as wild-type B. burgdorferi. Are the genes encoding the chbABC transporter expressed in early log phase?

RESPONSE: While we would consider this early-mid, this is a good point. *chbC* production is regulated by various factors[38-41], appreciable levels are present during early-mid log

phase[42] and the transporter is functional when bacteria are grown to late-log[39,41]. Most importantly, Arnold et al.[42] performed the most extensive transcriptomic study in the field—biological triplicate RNA-seq analysis of all genes expressed in early (10^6 cells/mL), mid (10^7 cells/mL), and late (10^8 cells/mL) phase growth[42]. While their definition of cell density relative to growth phase in much more in line with ours, the major take home here is that they detect the highest *chbC* transcript levels at the same density we use to analyze PG (10^7). We extracted *chbC* transcript data from supplemental table 2 of their manuscript and graphed it for the reviewers' benefit (Fig. X2A).

RNA-seq data can be at times misleading and we were a bit troubled by the large variability in values between biological replicates (Fig. X2A).To further address this comment, we have performed RT-PCR as a qualitative assessment of transporter production (Fig. X2B-C, below). We extracted RNA from 5A3 and 5A3/*chbC* cultures at densities of both 10^7 cells/mL (65 mL) and 10^8 (15 mL). After normalizing for total RNA differences between samples, we performed RT-PCR for targets *chbC* and the constitutively express control *flaB.* The relative fold change (Fig. X2B) and final products (Fig. X2C) are shown on a gel. The results below show production of *chbC* under early-mid and late growth, with highest occurring for the early-mid log sample (Fig. X2B-C), consistent with RNA-seq data (Fig. X2A). We have supplied these data for the reviewers benefit but have excluded them for the revise manuscript. We have, however, added this point to the new discussion.

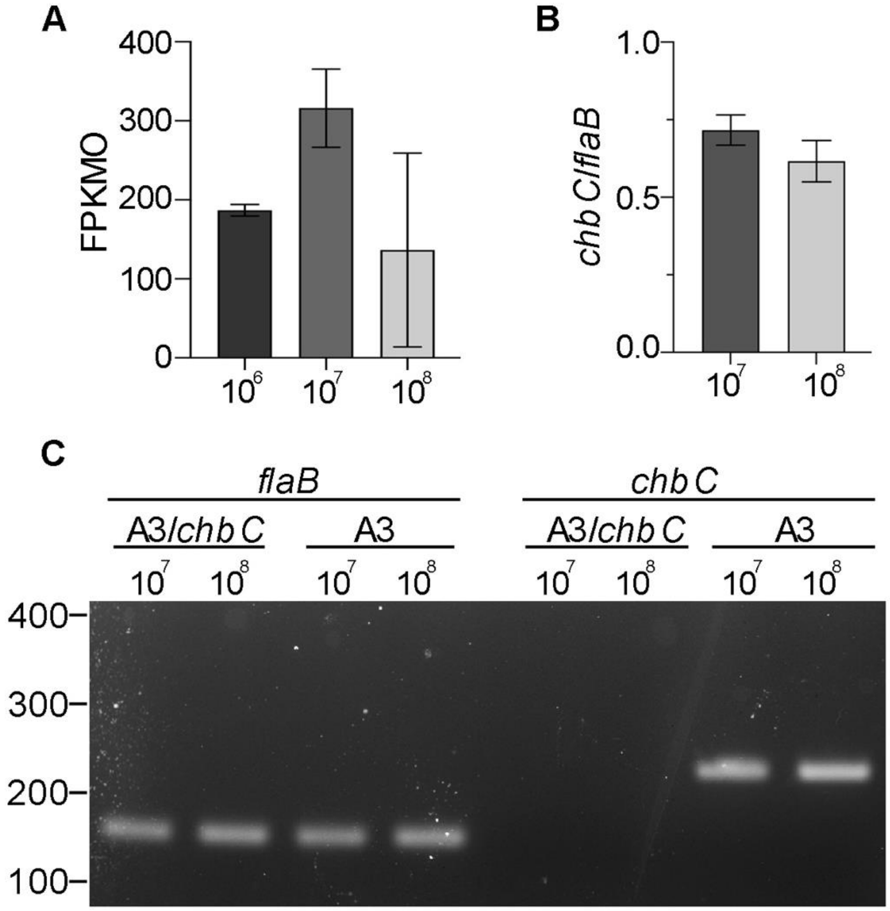
A
FPKMO
400
300
200
100
0
10^6
10^7
10^8
B
chbC/flaB
1.0
0.5
0.0
10^7
10^8
C
flaB
chbC
A3/chbC
A3
A3/chbC
A3
10^7
10^8
10^7
10^8
10^7
10^8
10^7
10^8
400
300
200
100

Figure X2. *B. burgdorferi chbC* production in culture as a function of cell density. (A) RNA-sequencing studies on *B. burgdorferi* collected in early (10^6 cells/mL), mid (10^7 cells/mL), and late (10^8 cells/mL) stages of growth. The relative *chbC* gene expression given in fragments per kilobase of ORF per million reads (FPKMO). The data shown were extracted from previously published studies (Reference 42, Table S2). (B) qRT-PC values of *chbC* production, relative to the constitutively produced *flaB.* After purifying RNA from both *B. burgdorferi* A3 and A3/*chbC* strains, cultured to a final density of 10^7 cell/mL (65mL culture) and 10^8 cell/mL (15mL culture), we added equal amounts of RNA to RT-PCR reactions using primers specific to *chbC* and *flaB.* (C) DNA agarose gel electrophoresis results upon completion of the experiment performed in B. Numbers and dashes (left) correspond to molecular weight ladder Note: We added 15 uL of the *chbC* product and 7.5 uL of *flaB* so as to not saturate the pixel intensity of latter product.

Were these experiments ever carried out in BSK II media deficient in components that are speculated sources of chitobiose (yeastolate)?

> No, they were not performed in BSK lacking yeast extract/chitobiose (see above). This was something we attempted once we determined the relationship between G-G-anhM and *chbC but* could never see culture expansion sufficient to perform analysis. We did notice a slight increase initially, but those cultures decline and number and ultimately died. To confirm, we have repeated the experiment by monitoring growth in culture media with and without autolyzed yeast (Fig. X3A). Even after 14 days we did not observe culture expansion, and cell densities decreased due to death. This is at odds with a single study published in *BMC Microbiology* [43], which suggested that after a substantial delay, some outgrowth can occur in the absence of yeast extract. When we reached out to the authors of that work to acquire their strains and test our culture results more than one year ago, they stated that the strains no longer existed and they had left the field.

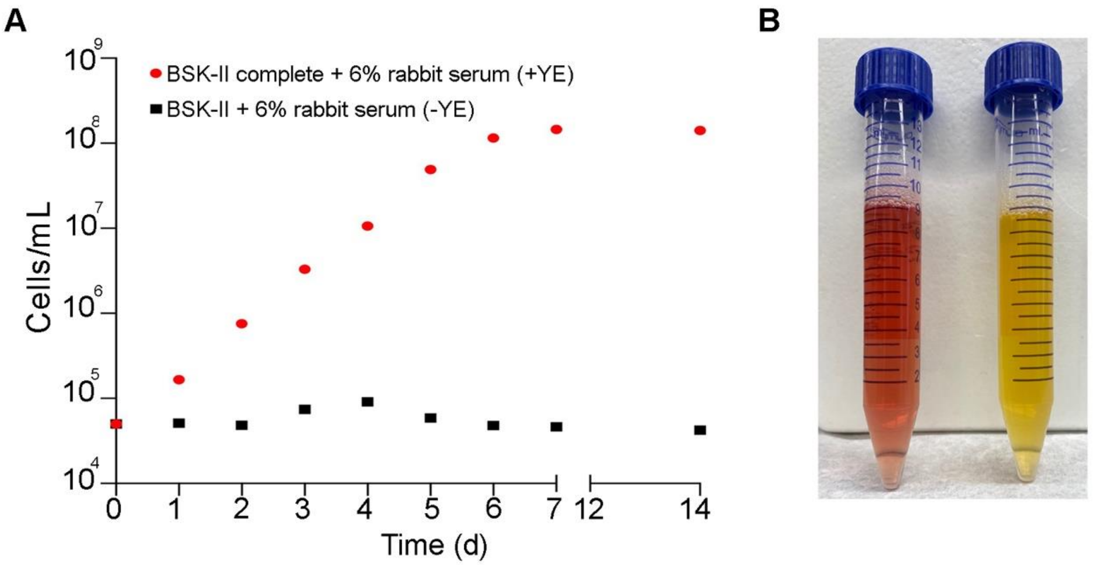

Figure X3. *B. burgdorferi* 5A3 was cultured in BSK-II complete media containing 6% rabbit serum (+ yeast extract, (+YE), red circles)) or in identical culture media lacking yeastolate (- YE, black squares). Each culture was enumerated by hemocytometer daily in triplicate and the mean determined and shown as a single data point. On day 14 a picture was taken of each culture (-YE left, +YE right), which demonstrates the media acidification—an indirect measure of growth—in +YE culture media, but no considerable change was observed in -YE culture media

Decision Letter, second revision:

Dear Professor Jutras

Thank you for submitting the revised version of your Article entitled "An abundant tick sugar helps shape the unusual peptidoglycan cell-wall of Borrelia burgdorferi, the Lyme disease spirochete" for consideration. Please accept my sincere apologies for the lengthy assessment time.

I have now read in full your rebuttal and the revised manuscript, and regret to inform you that after careful and due consideration, we have decided that we will not be sending the manuscript back to our referees and will no longer be able to consider it for publication in Nature Microbiology.

In this case, while we appreciate the revisions that you have made and the additional work included in the latest version of the manuscript, in the absence of experimental proof that the media contained chitobiose and a more convincing link between tick chitobiose and the peptidoglycan of Borrelia burgdorferi, we do not feel that the concerns raised by the referees (and identified by the editors as

being of key importance) have been satisfactorily addressed. We therefore feel that the paper would find a more suitable outlet in another journal.

Please be assured that this editorial decision does not represent a criticism of the quality of your work, nor are we questioning its value to others working in this area. We hope that you will rapidly receive a more favourable response elsewhere.

I am sorry that we cannot respond more positively on this occasion. Should you wish I can consult my colleagues at Nature Communications to determine whether they would return this version of the manuscript back to the original reviewers.

Yours sincerely

{redacted}

Author Rebuttal, second revision:

Reviewer #1 (Remarks to the Author):

I am satisfied with the revised version of the manuscript. I believe the work with be of substantial interest within the field.

RESPONSE: Many thanks for your insightful comments and support.

Reviewer #2 (Remarks to the Author):

Overall, I am satisfied with the authors' response; however, I am still somewhat concerned with the interpretations drawn from the AFM data. While the values for the Young's modulus determined here is similar to that found previously for E. coli, it is important to note that these are on dehydrated sacculi. In the previous work on E. coli, it was found that hydrated sacculi had a Young's modulus that was an order of magnitude smaller than when it is dry. This would suggest that the physiological case (when the bacteria are alive) has a vastly different Young's modulus than what is measured here, and it may even be likely that when hydrated the mutant PG is softer (rather than stiffer) than the wildtype.

RESPONSE: This is a good point. At this time, AFM measurements in fluid are very challenging for the dimensions of the *B. burgdorferi* sacculi. We have emphasized in the

discussion that we are comparing values “obtained using similar sample processing and data acquisition conditions’ and stated the values of each, so that there is no confusion to the reader.

Reviewer #3 (Remarks to the Author):
The authors have sufficiently addressed the concerns raised by the reviewers. This is excellent work that will be of great general interest to the field of bacteriology.

RESPONSE: Thanks so much!

Decision Letter, third revision:

Dear Dr. Jutras,

Thank you for submitting your revised manuscript "An abundant tick sugar helps shape the unusual peptidoglycan cell-wall of Borrelia burgdorferi, the Lyme disease spirochete" (NMICROBIOL-21020523C-Z). It has now been seen by the original referees and their comments are below. The reviewers find that the paper has improved in revision, and therefore we'll be happy in principle to publish it in Nature Microbiology, pending minor revisions to satisfy the referees' final requests and to comply with our editorial and formatting guidelines.

Reviewer 2 recommends clarification in the text with regards to Youngs modulus. Can you MAKE THE CHANGE and send me a finalized word document with this change incorporated so I can make my final editing suggestions on that more final version?

We are now performing detailed checks on your paper and will send you a checklist detailing our editorial and formatting requirements in about a week. Please do not upload the final materials and make any revisions until you receive this additional information from us.

Thank you again for your interest in Nature Microbiology Please do not hesitate to contact me if you have any questions.

Sincerely,

{redacted}

Reviewer #1 (Remarks to the Author):

I am satisfied with the revised version of the manuscript. I believe the work with be of substantial

interest within the field.

Reviewer #2 (Remarks to the Author):

Overall, I am satisfied with the authors' response; however, I am still somewhat concerned with the interpretations drawn from the AFM data. While the values for the Young's modulus determined here is similar to that found previously for E. coli, it is important to note that these are on dehydrated sacculi. In the previous work on E. coli, it was found that hydrated sacculi had a Young's modulus that was an order of magnitude smaller than when it is dry. This would suggest that the physiological case (when the bacteria are alive) has a vastly different Young's modulus than what is measured here, and it may even be likely that when hydrated the mutant PG is softer (rather than stiffer) than the wildtype.

Reviewer #3 (Remarks to the Author):

The authors have sufficiently addressed the concerns raised by the reviewers. This is excellent work that will be of great general interest to the field of bacteriology.

Decision Letter, final checks:

Dear Brandon,

Thank you for your patience as we've prepared the guidelines for final submission of your Nature Microbiology manuscript, "An abundant tick sugar helps shape the unusual peptidoglycan cell-wall of Borrelia burgdorferi, the Lyme disease spirochete" (NMICROBIOL-21020523C-Z). Please carefully follow the step-by-step instructions provided in the attached file, and add a response in each row of the table to indicate the changes that you have made. Please also check and comment on any additional marked-up edits we have proposed within the text. Ensuring that each point is addressed will help to ensure that your revised manuscript can be swiftly handed over to our production team.

We would like to start working on your revised paper, with all of the requested files and forms, as soon as possible (preferably within two weeks). Please get in contact with us if you anticipate delays.

When you upload your final materials, please include a point-by-point response to any remaining reviewer comments.

If you have not done so already, please alert us to any related manuscripts from your group that are under consideration or in press at other journals, or are being written up for submission to other journals (see: https://www.nature.com/nature-research/editorial-policies/plagiarism#policy-on-duplicate-publication for details).

In recognition of the time and expertise our reviewers provide to Nature Microbiology's editorial process, we would like to formally acknowledge their contribution to the external peer review of your manuscript entitled "An abundant tick sugar helps shape the unusual peptidoglycan cell-wall of

Borrelia burgdorferi, the Lyme disease spirochete". For those reviewers who give their assent, we will be publishing their names alongside the published article.

Nature Microbiology offers a Transparent Peer Review option for new original research manuscripts submitted after December 1st, 2019. As part of this initiative, we encourage our authors to support increased transparency into the peer review process by agreeing to have the reviewer comments, author rebuttal letters, and editorial decision letters published as a Supplementary item. When you submit your final files please clearly state in your cover letter whether or not you would like to participate in this initiative. Please note that failure to state your preference will result in delays in accepting your manuscript for publication.

Cover suggestions

As you prepare your final files we encourage you to consider whether you have any images or illustrations that may be appropriate for use on the cover of Nature Microbiology.

Covers should be both aesthetically appealing and scientifically relevant, and should be supplied at the best quality available. Due to the prominence of these images, we do not generally select images featuring faces, children, text, graphs, schematic drawings, or collages on our covers.

We accept TIFF, JPEG, PNG or PSD file formats (a layered PSD file would be ideal), and the image should be at least 300ppi resolution (preferably 600-1200 ppi), in CMYK colour mode.

If your image is selected, we may also use it on the journal website as a banner image, and may need to make artistic alterations to fit our journal style.

Please submit your suggestions, clearly labeled, along with your final files. We'll be in touch if more information is needed.

Nature Microbiology has now transitioned to a unified Rights Collection system which will allow our Author Services team to quickly and easily collect the rights and permissions required to publish your work. Approximately 10 days after your paper is formally accepted, you will receive an email in providing you with a link to complete the grant of rights. If your paper is eligible for Open Access, our Author Services team will also be in touch regarding any additional information that may be required to arrange payment for your article.

Please note that you will not receive your proofs until the publishing agreement has been received through our system.

Please note that <i>Nature Microbiology</i> is a Transformative Journal (TJ). Authors may publish their research with us through the traditional subscription access route or make their paper immediately open access through payment of an article-processing charge (APC). Authors will not be required to make a final decision about access to their article until it has been accepted. Find out more about Transformative Journals

<B>Authors may need to take specific actions to achieve <a

href="https://www.springernature.com/gp/open-research/funding/policy-compliance-faqs"> compliance with funder and institutional open access mandates. For submissions from January 2021, if your research is supported by a funder that requires immediate open access (e.g. according to Plan S principles) then you should select the gold OA route, and we will direct you to the compliant route where possible. For authors selecting the subscription publication route our standard licensing terms will need to be accepted, including our self-archiving policies. Those standard licensing terms will supersede any other terms that the author or any third party may assert apply to any version of the manuscript.

For information regarding our different publishing models please see our Transformative Journals page. If you have any questions about costs, Open Access requirements, or our legal forms, please contact ASJournals@springernature.com.

Please use the following link for uploading these materials:
{redacted}

If you have any further questions, please feel free to contact me.

Best regards,
{ redacted}

Reviewer #1:
Remarks to the Author:
I am satisfied with the revised version of the manuscript. I believe the work with be of substantial interest within the field.

Reviewer #2:
Remarks to the Author:
Overall, I am satisfied with the authors' response; however, I am still somewhat concerned with the interpretations drawn from the AFM data. While the values for the Young's modulus determined here is similar to that found previously for E. coli, it is important to note that these are on dehydrated sacculi. In the previous work on E. coli, it was found that hydrated sacculi had a Young's modulus that was an order of magnitude smaller than when it is dry. This would suggest that the physiological case (when the bacteria are alive) has a vastly different Young's modulus than what is measured here, and it may even be likely that when hydrated the mutant PG is softer (rather than stiffer) than the wildtype.

Reviewer #3:

Remarks to the Author:
The authors have sufficiently addressed the concerns raised by the reviewers. This is excellent work that will be of great general interest to the field of bacteriology.

Final Decision Letter:

Dear Professor Jutras,

I am pleased to accept your Article "The unusual cell wall of the Lyme disease spirochaete Borrelia burgdorferi is shaped by a tick sugar" for publication in Nature Microbiology. Thank you for having chosen to submit your work to us and many congratulations.

Before your manuscript is typeset, we will edit the text to ensure it conforms to house style.

Once your manuscript is typeset and you have completed the appropriate grant of rights, you will receive a link to your electronic proof via email with a request to make any corrections within 48 hours. If, when you receive your proof, you cannot meet this deadline, please inform us at rjsproduction@springernature.com immediately. Once your paper has been scheduled for online publication, the Nature press office will be in touch to confirm the details.

Acceptance of your manuscript is conditional on all authors' agreement with our publication policies (see www.nature.com/nmicrobiolate/authors/gta/content-type/index.html). In particular your manuscript must not be published elsewhere and there must be no announcement of the work to any media outlet until the publication date (the day on which it is uploaded onto our website).

Please note that <i>Nature Microbiology</i> is a Transformative Journal (TJ). Authors may publish their research with us through the traditional subscription access route or make their paper immediately open access through payment of an article-processing charge (APC). Authors will not be required to make a final decision about access to their article until it has been accepted. Find out more about Transformative Journals

<B>Authors may need to take specific actions to achieve compliance with funder and institutional open access mandates. For submissions from January 2021, if your research is supported by a funder that requires immediate open access (e.g. according to Plan S principles) then you should select the gold OA route, and we will direct you to the compliant route where possible. For authors selecting the subscription publication route our standard licensing terms will need to be accepted, including our self-archiving policies. Those standard licensing terms will supersede any other terms that the author or any third party may assert apply to any version of the manuscript.

In approximately 10 business days you will receive an email with a link to choose the appropriate publishing options for your paper and our Author Services team will be in touch regarding any

additional information that may be required.

You will not receive your proofs until the publishing agreement has been received through our system.

If you have any questions about our publishing options, costs, Open Access requirements, or our legal forms, please contact ASJournals@springernature.com

An online order form for reprints of your paper is available at https://www.nature.com/reprints/author-reprints.html. All co-authors, authors' institutions and authors' funding agencies can order reprints using the form appropriate to their geographical region.

We welcome the submission of potential cover material (including a short caption of around 40 words) related to your manuscript; suggestions should be sent to Nature Microbiology as electronic files (the image should be 300 dpi at 210 x 297 mm in either TIFF or JPEG format). Please note that such pictures should be selected more for their aesthetic appeal than for their scientific content, and that colour images work better than black and white or grayscale images. Please do not try to design a cover with the Nature Microbiology logo etc., and please do not submit composites of images related to your work. I am sure you will understand that we cannot make any promise as to whether any of your suggestions might be selected for the cover of the journal.

You can now use a single sign-on for all your accounts, view the status of all your manuscript submissions and reviews, access usage statistics for your published articles and download a record of your refereeing activity for the Nature journals.

To assist our authors in disseminating their research to the broader community, our SharedIt initiative provides you with a unique shareable link that will allow anyone (with or without a subscription) to read the published article. Recipients of the link with a subscription will also be able to download and print the PDF.

As soon as your article is published, you will receive an automated email with your shareable link.
